# An intrinsic cytoskeletal oscillator establishes neuronal polarity

**DOI:** 10.1038/s41586-026-10755-6

**Published:** 2026-07-08

**Authors:** Tien-chen Lin, Charlotte H. Coles, Eissa Alfadil, Florian Fäßler, Andreas Husch, Sebastian Dupraz, Thorben Pietralla, Akihiro Narita, Max Schelski, Kevin C. Flynn, Sina Stern, Christoph Möhl, Brett J. Hilton, Franz Vauti, Hans-Henning Arnold, Florian K. M. Schur, Frank Bradke

**Affiliations:** 1 Laboratory for Axon Growth and Regeneration, German Center for Neurodegenerative Diseases (DZNE), Bonn, Germany; 2 Institute of Science and Technology Austria (ISTA), Klosterneuburg, Austria; 3 International Max Planck Research School for Brain and Behavior, Bonn, Germany; 4 Division of Biological Science, Graduate School of Science, Nagoya University, Nagoya, Japan; 5 Image and Data Analysis Facility, German Center for Neurodegenerative Diseases (DZNE), Bonn, Germany; 6 International Collaboration on Repair Discoveries (ICORD), Djavad Mowafaghian Centre for Brain Health, and Department of Cellular and Physiological Sciences, Faculty of Medicine, University of British Columbia, Vancouver, British Columbia Canada; 7 Department of Cellular and Molecular Neurobiology, Technische Universität Braunschweig, Braunschweig, Germany; 8 Present Address: Biopharm Discovery, GlaxoSmithKline, Stevenage, UK; 9 Present Address: Department of Integrated Structural Biology, Institut de Génétique et de Biologie Moléculaire et Cellulaire (IGBMC), Illkirch, France; 10 Present Address: CaseBioscience, Woodbury, MN USA

**Keywords:** Cell polarity, Neuronal development

## Abstract

Neurons acquire polarity by specifying one neurite as the axon, whereas the others become dendrites. But how this fundamental asymmetry is established remains unclear^1^. Neuronal polarization has been thought to rely primarily on growth cones that sense external cues^2^. Here we show that growth cones alone do not direct this process and that the soma acts as a central organizer of neuronal polarization. Using live imaging and genetic loss-of-function approaches in vivo, combined with optogenetic control and local cytoskeletal perturbations in cultured neurons, we uncover a soma-initiated oscillatory program that primes axon selection. Periodic actin branching that depends on the actin-related protein 2/3 (ARP2/3) complex at the soma remodels a global actomyosin network, thereby generating an actin wave that retracts neurites before propagating into a single neurite tip. Exposure to this wave relaxes local actomyosin contractility, which drives a transient microtubule-based protrusion and biases this neurite towards axon fate. As the cell exits this oscillatory stage, this neurite can overcome global inhibition and extend independently of ARP2/3, whereas actomyosin activity suppresses axon formation in the remaining neurites so that they subsequently become dendrites. This soma-driven mechanism ensures the emergence of a single axon independent of environmental cues and underpins the unidirectional information flow in neuronal circuits.

## Abstract (web summary)

The ARP2/3 complex works together with actomyosin in an oscillatory program at the soma to govern neuronal polarization.

## Main

The polarity of neurons governs information flow across neuronal circuits. Cortical pyramidal neurons establish polarity by breaking cellular symmetry to form a single axon and multiple dendrites^1^. Before a single axon forms, multiple neurites emanate from the soma in vitro and in vivo^3,4^. Although all of these neurites can become the axon, only one realizes this fate^5–7^. How one neurite is singled out to become the axon remains unclear.

Extrinsic signals guide neuronal polarization, including extracellular matrix components and the growth factors neurotrophin 3 (NT3) and transforming growth factor-β (TGFβ)^8–10^. As growth cones detect and respond to such cues^1,2^, they have long been thought to regulate polarization through ligand–receptor signalling pathways involving effectors such as glycogen synthase kinase 3 (GSK3)^11,12^. In this growth-cone-centred view, both neuronal polarity and axon formation would be dictated by the distribution of external guidance molecules, which raises the possibility that multiple neurites could adopt axon identity under uniform exposure. However, this model is difficult to reconcile with the fact that neurons reliably form only a single axon in the developing brain, an environment rich in growth-promoting factors. Moreover, the initial site of axon emergence is not dictated by the eventual direction of guided axon extension^4^. Even when two neurites of a neuron simultaneously contact growth-promoting substrates, only one becomes the axon and the other is restrained^8^. How coordination among neurites is achieved is unclear.

**Fig. 1:**
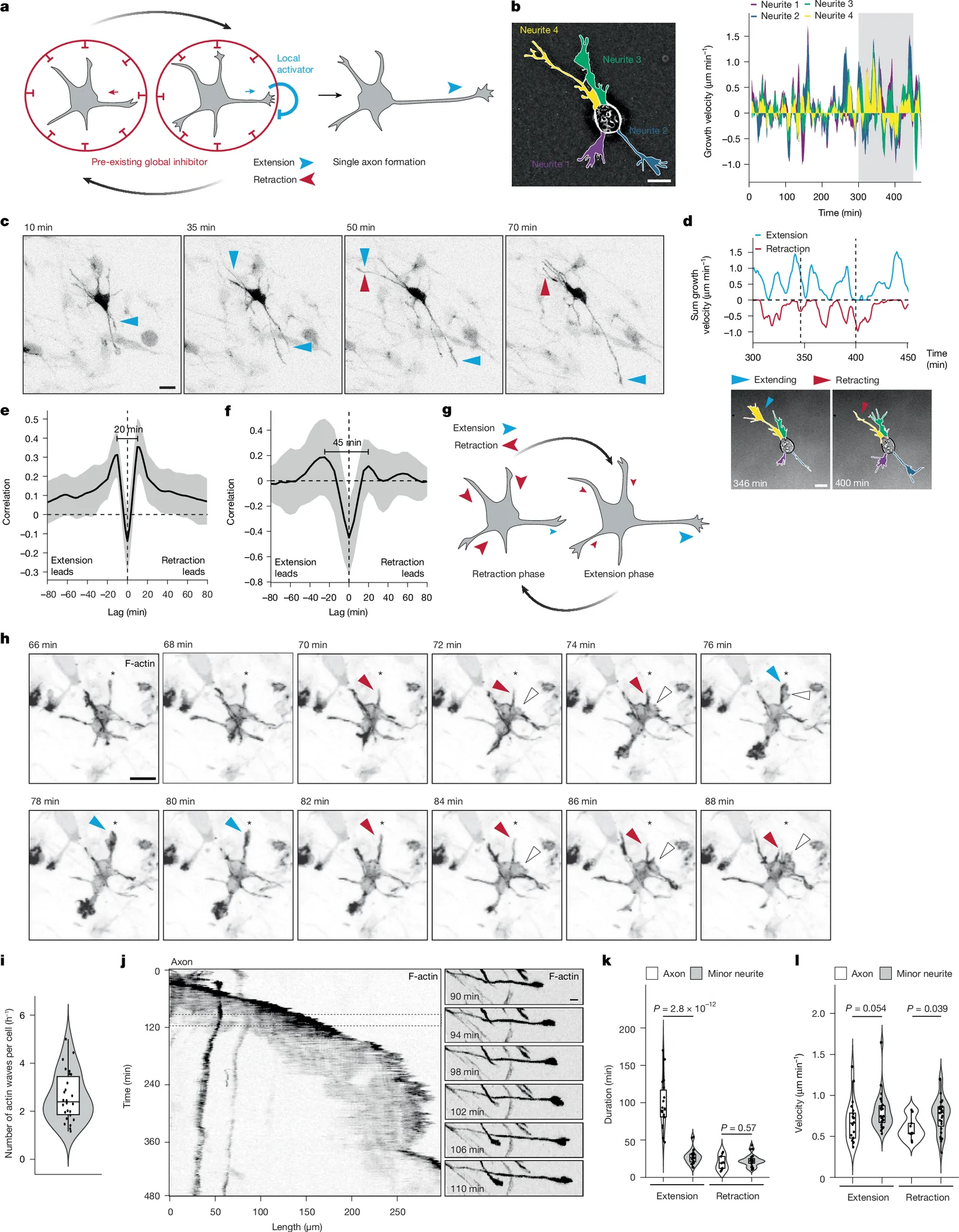
Oscillatory neurite growth, but not persistent axonal growth, is associated with actin waves. **a**, Activator–inhibitor model of neuronal polarization. Global inhibition pre-exists by default. Local activation that antagonizes global inhibition and enables transient extension of a single neurite arises stochastically. **b**, Left, brightfield and segmented image of a DIV-1 unpolarized neuron in 2D culture. Neurons of four independent experiments recapitulated the results. Right, stacked area plot of the velocity profiles derived from Extended Data Fig. 1a, representing the summed growth activities. The grey shaded area marks the time window zoomed in **d**. **c**, Serial images of a neuron expressing the membrane-targeting motif of LYN (LYN(TM)) fused with mNeonGreen in an embryonic day 15.5 (E15.5) acute cortical slice. Blue and red arrowheads point to the extending and retracting neurites, respectively. Neurons of three independent experiments recapitulated the results. **d**, Time profiles of the summed neurite extension and retraction velocities in 2D cultured neurons at the selected time window in **b**. The representative frames at the time points indicated by the dashed vertical lines are shown below. **e**, Neurite extension–retraction cross-correlation in 2D cultured neurons. Values are plotted as the mean ± s.d. *n* = 101 neurons from 4 experiments. **f**, Neurite extension–retraction cross-correlation in an acute cortical slice. Values are plotted as the mean ± s.d. *n* = 49 neurons from 3 experiments. **g**, Illustration of neurite extension–retraction oscillation. **h**, Serial snapshots of unpolarized neurons expressing Lifeact–mNeonGreen (to label F-actin) in an E15.5 acute cortical slice. White arrowheads point to the emerging somatic actin wave; asterisks mark the starting position of the tip; blue and red arrowheads point to the adjacent extending and retracting neurites, respectively. Neurons of three independent experiments recapitulated the results. **i**, Frequency of somatic actin waves in unpolarized neurons of an E15.5 acute cortical slice. *n* = 26 neurons from 3 experiments. **j**, Left, kymograph of axonal growth in a cortical slice visualized with Lifeact–mNeonGreen. Right, selected serial snapshots of the axon. The dashed lines in the kymographs mark the period of the serial snapshots. **k**, Quantification of extension and retraction duration of axon and minor neurites in an E15.5 cortical slice. *n* = 28 unpolarized neurons and 18 axons from 3 experiments. Two-tailed Mann–Whitney test. **l**, Quantification of extension and retraction velocities of axon and minor neurites. The data source is the same as in **k**. Two-tailed Mann–Whitney test. For the box-violin plots (**i**,**k**,**l**), the centre line is the median, the box shows 25th–75th percentiles and whiskers are 1.5× the interquartile range (IQR). The violin spans minimum to maximum values. Exact *P* values are indicated. Scale bars, 10 μm (**b**,**c**,**d**,**h**,**j**). Source data

Here, drawing on the ability of cytoskeletal networks to oscillate^13^ and incorporating this into models of spontaneous cell polarization^14–16^, we propose that actin dynamics generate oscillatory neurite behaviour to establish an intrinsic bias for axon selection (Fig. 1a). Using cortical pyramidal neurons, we show that the antagonistic interplay between ARP2/3 and actomyosin drives oscillatory neurite growth before polarization. Periodic ARP2/3 activity at the soma produces actin waves that trigger myosin-II-dependent retraction of all neurites, followed by selective growth of the neurite into which the wave propagates. When the wave reaches the tip of that neurite, ARP2/3 locally opposes actomyosin contractility, which facilitates microtubule protrusion and transient extension. The nascent axon, once stabilized by microtubules, becomes resistant to retraction and no longer requires ARP2/3 for continued growth. This cytoskeleton-driven, cell-intrinsic mechanism resolves the longstanding question of how neurons reliably form a single axon in the growth-promoting environment of the developing brain.

## Neurons extend and retract before polarization

**Extended Data Fig. 1:**
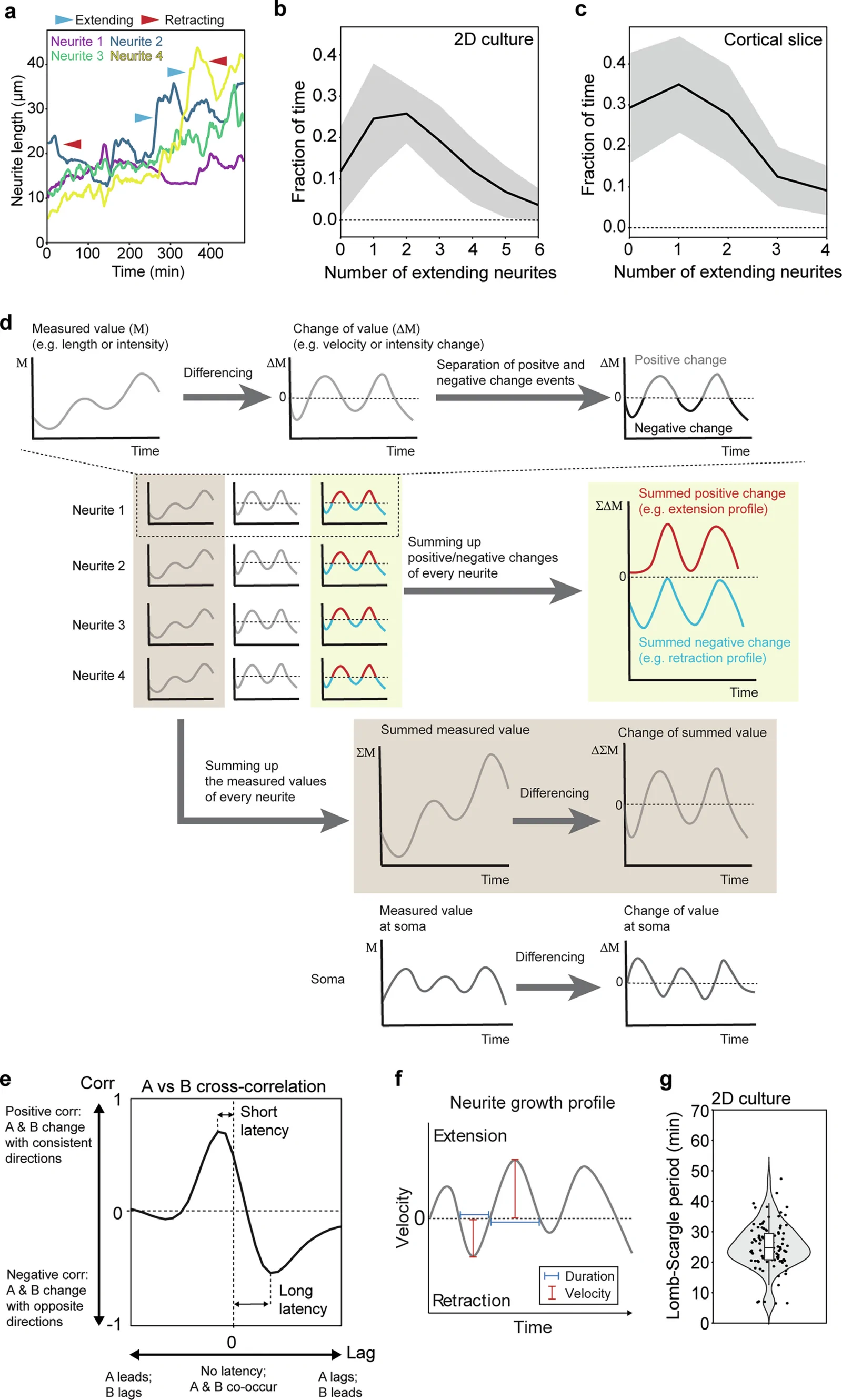
Neurite growth profiling and cross-correlation analysis. a. Length profiles of individual neurites. The blue and red arrowheads point to the extending and retracting neurites, respectively. b. Relative occurrence of neurons in 2D culture having the indicated number of extending neurites over the observation time. Values are plotted as mean ± s.d. *n* = 101 neurons from 4 independent experiments. c. Relative occurrence of neurons in acute cortical slice culture having the indicated number of extending neurites over the observation time. Values are plotted as mean ± s.d. *n* = 59 neurons from 3 independent experiments. d. Schematic of processing measurements and data transformation for cross-correlation analysis. Detail procedure is described in Materials and Methods. e. Interpretation guide for cross-correlation. f. Scheme of quantification of neurite growth velocities and durations. g. Period of neurite growth oscillation determined by Lomb-Scargle periodogram analysis. *n* = 98 neurons from 4 independent experiments. Data are shown as box-violin plot (center line, median; box, 25th–75th percentiles; whiskers, 1.5 × IQR; violin spans min to max).

Oscillatory processes can orchestrate polarity in non-neuronal cells^17,18^. We therefore asked whether neurites exhibit oscillatory growth before neurons polarize. Consistent with previous observations^3,4,19^, neurite growth alternated between extension and retraction in both cultured unpolarized hippocampal neurons and embryonic cortical slices (Fig. 1b,c and Supplementary Videos 1 and 2). Machine-learning-assisted segmentation followed by growth-profiling analysis showed that only one neurite extended at a time before subsequently retracting (Fig. 1b and Extended Data Fig. 1a–c). Correlation analyses of extension and retraction dynamics further showed that neurites oscillated between alternating phases of extension and retraction in both culture and slice preparations (Fig. 1d–f and Extended Data Fig. 1d,e). During the extension phase, defined by acceleration of the extending neurite, the other neurites slowed their retraction. Conversely, during the retraction phase, marked by increased retraction speed in retracting neurites, the extending neurite decelerated (Fig. 1g). Together, these results show that neurites undergo coordinated oscillatory growth before polarization.

## Actin fluctuations oscillate with neurite growth

Such oscillatory behaviour of transient polarization could be driven by an activator–inhibitor system^14,15,20^. That is, a neurite that becomes locally activated extends as the transient axon, and then it is captured and retracted through global inhibition. As motile non-neuronal cells polarize by exiting from actin-driven oscillatory protrusions to directional movement^17,18,21^, we speculated that an actin-based system drives oscillatory growth.

To test the involvement of actin networks, we performed in utero electroporation (IUE) of cortical neurons with a plasmid encoding Lifeact fused to a fluorescent protein as a marker for filamentous actin (F-actin)^22^. We then performed time-lapse imaging of these neurons in acute cortical slices. Actin fluctuations in the soma were associated with oscillatory neurite growth (Fig. 1h and Supplementary Video 3). Consistent with studies showing that actin waves occur in cultured neurons^23,24^, unpolarized neurons in cortical slices repeatedly formed actin waves at the perisomatic region (Fig. 1i). Notably, wave formation at the soma was associated with retraction of the neurite adjacent to the wave-forming site, and the wave-receiving neurite then regrew (Fig. 1h and Supplementary Video 3). By contrast, in polarized neurons of cortical slices, axons did not acquire waves but elongated persistently, with extension times fourfold longer than retraction times (Fig. 1j–l, Extended Data Fig. 1f and Supplementary Video 4).

**Fig. 2:**
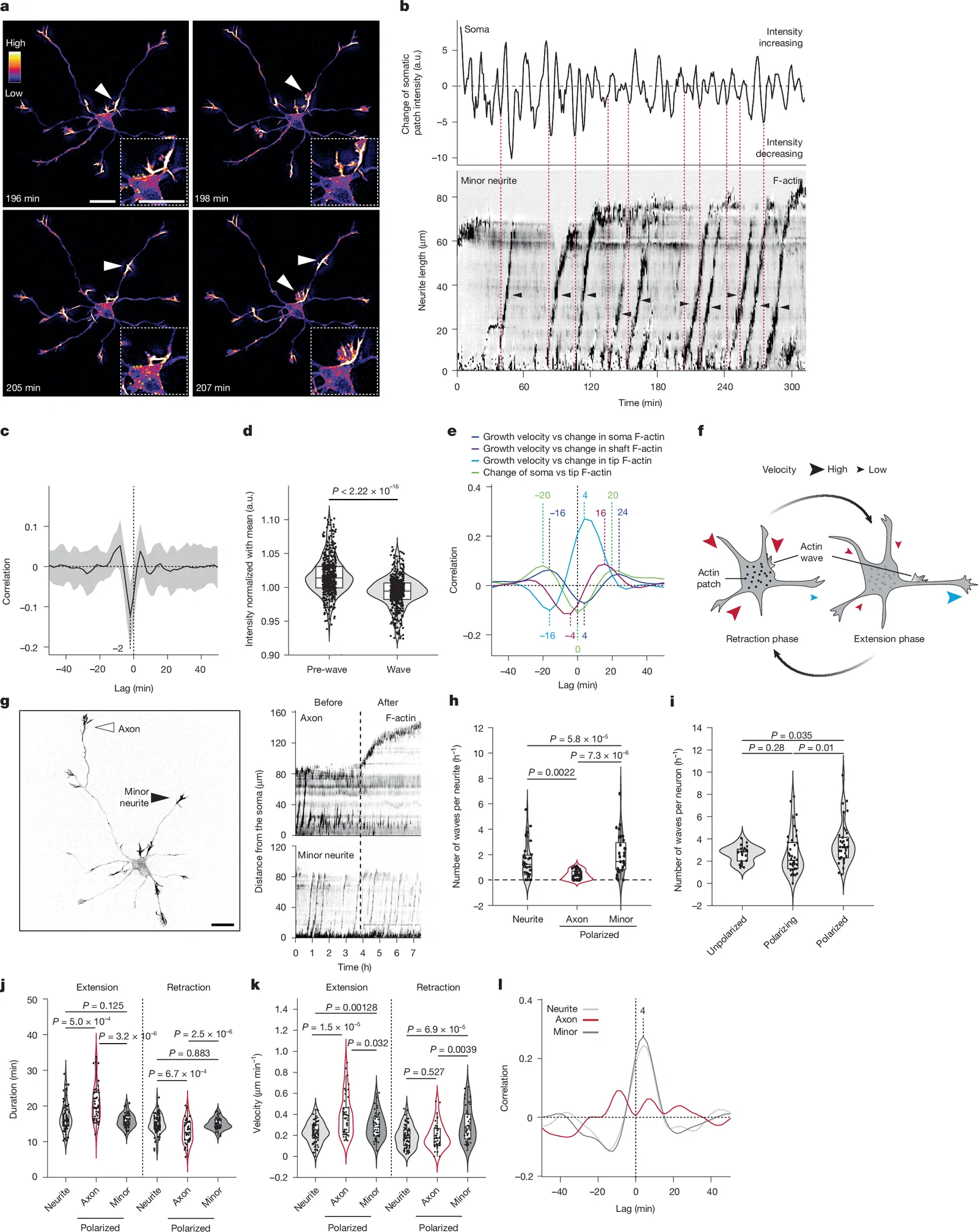
Oscillatory neurite growth is coupled with actin fluctuations originating from somatic actin patches. **a**, Images of a DIV-1 neuron expressing Lifeact–mScarlet (F-actin). White arrowheads mark the actin wave that emerged at the soma and moved to the adjacent neurite. Insets, the magnified soma area. Asterisks mark the actin patches. Neurons of four independent experiments recapitulated the results. **b**, Profile of somatic actin-patch intensity over time (top) of the neuron in **a** and growth kymograph of the selected neurite (bottom). Dashed vertical lines mark the time points at which actin waves emerged. Black arrowheads mark the traces of moving actin waves. **c**, Cross-correlation between the changes in soma actin-patch intensity and the binarized occurrence of actin waves. Values are plotted as the mean ± s.d. *n* = 23 neurons from 3 experiments. **d**, Quantification of somatic actin intensity (in arbitrary units (a.u.)) at the time points of wave emergence and before. *n* = 514 wave events of 23 neurons from 4 experiments. Two-tailed paired *t*-tests. **e**, Cross-correlation of the indicated pairs of temporal profiles. Correlation peaks and valleys are labelled with lag time. Values are plotted as the mean of correlation functions of *n* = 146 neurons from 5 experiments. The s.d. area is shown in Extended Data Fig. 2c. **f**, Schematic of neurite growth oscillation and sequential actin fluctuations in WT neurons. The increase in somatic actin intensity is associated with a retraction phase, whereas the translocation of a wave from the soma to a single neurite is associated with an extension phase. **g**, Left, same neuron as in **a**, but at the transition to polarization. Right, kymographs of the growth of the axon and the selected minor neurite before and after polarization. The dashed line marks the time point at which the axon showed persistent growth. **h**, Frequency of actin waves per neurite before polarization and per axon and minor neurite (minor) after polarization. *n* = 31 neurons from 5 experiments. Two-tailed paired Wilcoxon test. **i**, Frequency of actin waves per cell. *n* = 23 (unpolarized), 38 (polarizing) and 37 (polarized) neurons from 3 to 5 experiments. Two-tailed Mann–Whitney test. **j**, Extension and retraction duration of neurites before polarization, and of axon and minor neurites after polarization. *n* = 48 neurons from 8 experiments. Two-tailed paired Wilcoxon test. **k**, Extension and retraction velocities of neurites before polarization, and of axon and minor neurites after polarization. The data source is the same as in **j**. Two-tailed paired Wilcoxon test. **l**, Cross-correlation between neurite growth velocity and the change in actin intensity at neurite tips in neurons at the transition to polarization. The data source is the same as in **j**. For the box-violin plots (**d**,**h**–**k**), the centre line is the median, the box shows 25th–75th percentiles and the whiskers are 1.5× the IQR. The violin spans minimum to maximum values. Exact *P* values are indicated. Scale bars, 20 μm (**a**,**g**). Source data

**Extended Data Fig. 2:**
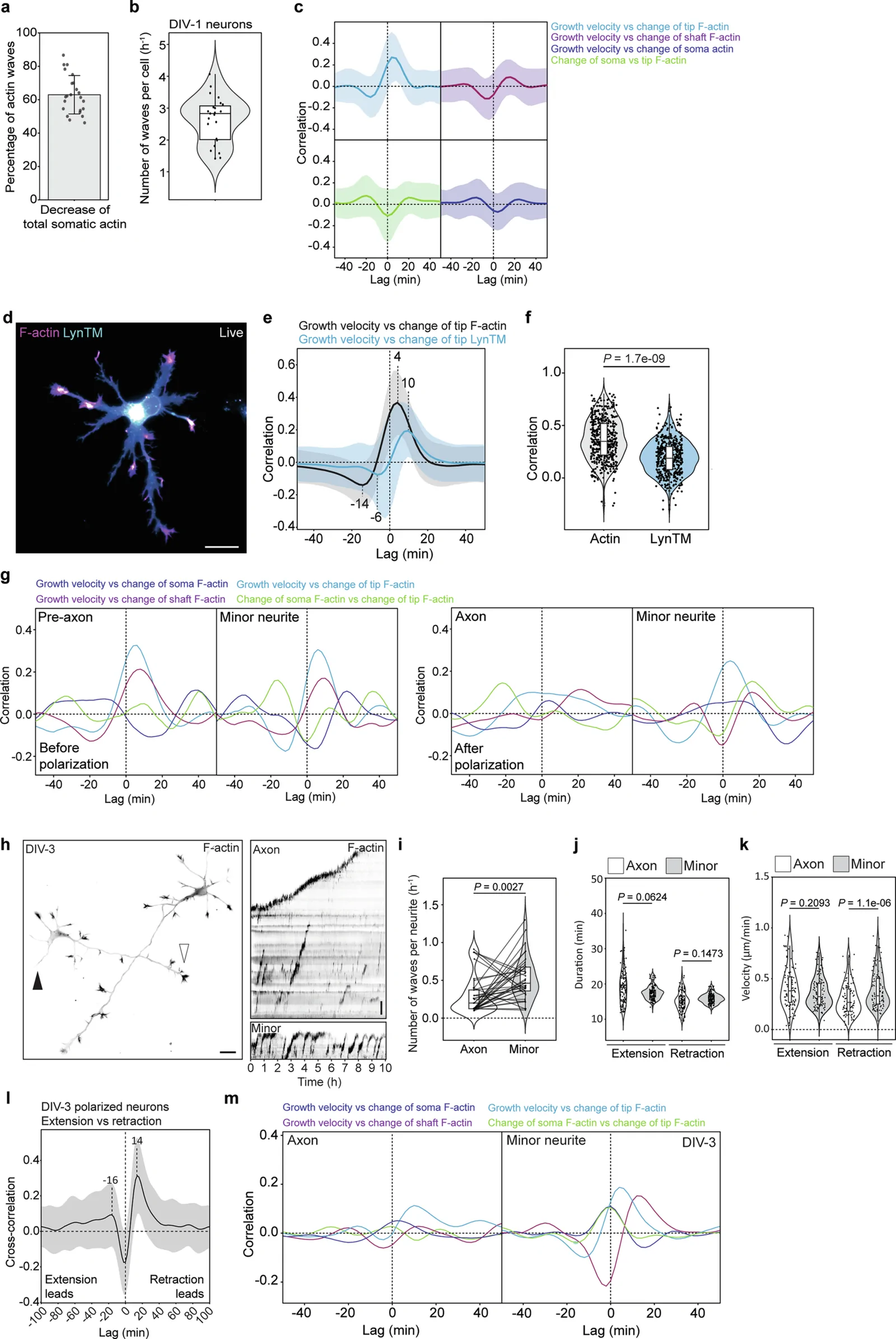
Local actin intensity profiling and cross-correlation analysis of polarizing and polarized neurons. a. Percentage of actin waves co-occurring with decreasing soma actin intensity. *n* = 23 neurons from 3 independent experiments. Values are plotted as mean ± s.d. b. Frequency of actin waves in cultured unpolarized neurons. *n* = 23 neurons from 3 independent experiments. c. Cross-correlation of temporal profiles of DIV-1 and DIV-2 unpolarized neurons. Same in Fig. 2e but with s.d. Values are plotted as mean ± s.d. *n* = 146 neurons from 5 independent experiments. d. Image of a living neuron expressing Lifeact-mScarlet and LYN(TM)-mNeonGreen. 4 independent experiments recapitulated the results. Scale bar, 20 μm. e. Cross-correlation of neurite growth velocity with the changes of actin and membrane marker LynTM at the neurite tips of individual neurites. Values are plotted as mean ± s.d. *n* = 509 neurites of 73 neurons from 4 independent experiments. f. Comparison of the correlation levels of the changes of actin and LYN(TM) with neurite growth velocity. Data points *n* = 509 neurites are of the same source as in **e**. Two-tailed Mann-Whitney test. g. Cross-correlation of temporal profiles of DIV-1 or DIV-2 neurons at the transition of polarization. Values are plotted as mean of correlation functions of *n* = 22 neurons from 5 experiments. h. Left: Two DIV-3 polarized neurons expressing Lifeact-mScarlet. White arrowhead: the axon; black arrowhead: one selected minor neurite. Right: kymographs of the growth of axon and the selected minor neurite. Scale bar, 20 μm. i. Frequency of actin waves per axon and minor neurite before and after polarization. *n* = 37 DIV-3 neurons from 3 experiments. Two-tailed paired Wilcoxon test. j. Extension and retraction duration of axon and minor neurites after polarization. *n* = 107 neurons from 4 experiments. Two-tailed paired Wilcoxon test. k. Extension and retraction velocities of axon and minor neurites after polarization. Data source is the same as in **j**. Two-tailed paired Wilcoxon test. l. Neurite extension-retraction cross-correlation. Values are plotted as mean ± s.d. *n* = 75 neurons from 4 experiments. m. Cross-correlation of temporal profiles of neurons after polarization. Values are plotted as mean of correlation functions. Data source is the same as in **l**. Box-violin plots (**b**, **f**, **i-k**): center line, median; box, 25th–75th percentiles; whiskers, 1.5 × IQR. Violin spans min to max. Exact *P* values are indicated.

Detailed analyses of the somatic actin fluctuations in cultured neurons revealed actin patches in the soma as the origin of the fluctuations (Fig. 2a,b and Supplementary Video 5). As with neurons of cortical slices, neurites retracted during wave formation (Fig. 2b). The actin wave moved into a single neurite (Fig. 2a,b), and after reaching the neurite tip, the growth cone became active and the neurite transiently extended (Fig. 2b). In parallel, actin patches at the soma increased again and initiated another actin wave. In line with theoretical patch-to-wave transformation of actin^25^, 63 ± 12% (mean ± s.d.) of wave-forming events followed a 5 ± 4% (mean ± s.d.) decrease in total somatic actin patch intensity (Fig. 2c,d and Extended Data Fig. 2a). Actin waves formed with a frequency of 2.6 ± 0.7 waves per hour (mean ± s.d.; Extended Data Fig. 2b), which matched the interval of 25 ± 7.5 min between extension and retraction phases (mean ± s.d.; Extended Data Fig. 1g). Cross-correlation analysis validated that oscillatory growth correlated with the cycles of actin fluctuations (Fig. 2e,f and Extended Data Fig. 2c) but not with the negative control (LYN fused with a fluorescent protein; Extended Data Fig. 2d–f).

As in cortical slices, the nascent axon in vitro hardly received waves during polarization (Fig. 2g,h), whereas the overall frequency of wave formation per neuron remained constant (Fig. 2i). Concomitantly, the axon showed increased extension rate and duration (Fig. 2j,k). By contrast, after polarization, minor neurites received more waves (Fig. 2g,h and Supplementary Video 5), and their retraction rate increased (Fig. 2j,k). The axon and the minor neurites kept these different characteristics after polarization (Extended Data Fig. 2h–k and Supplementary Video 6). Hence, after polarization, minor neurites are more restrained. Both growth oscillation (Extended Data Fig. 2l) and growth–actin correlation were substantially reduced in axons (Fig. 2l and Extended Data Fig. 2g,m).

Taken together, consistent with the possibility of an activator–inhibitor system at work, unpolarized neurons transiently polarize when one neurite acquires the wave from the perisomatic region followed by extension, which then retracts again. The cell exits this oscillatory stage to eventually polarize when the activator–inhibitor system is overcome through the decoupling of axonal growth from actin-wave-driven oscillatory growth.

## ARP2/3 distribution correlates with neurite growth

We next asked which actin-regulating mechanism could drive oscillatory growth and serve as an activator–inhibitor module. ARP2/3 nucleate branched actin networks in growth cones and form actin patches across diverse cell types^26–28^. Moreover, ARP2/3 have been implicated in both promoting and inhibiting axon growth in vitro^29,30^. We therefore proposed that local ARP2/3 activity couples actin remodelling to oscillatory growth.

**Fig. 3:**
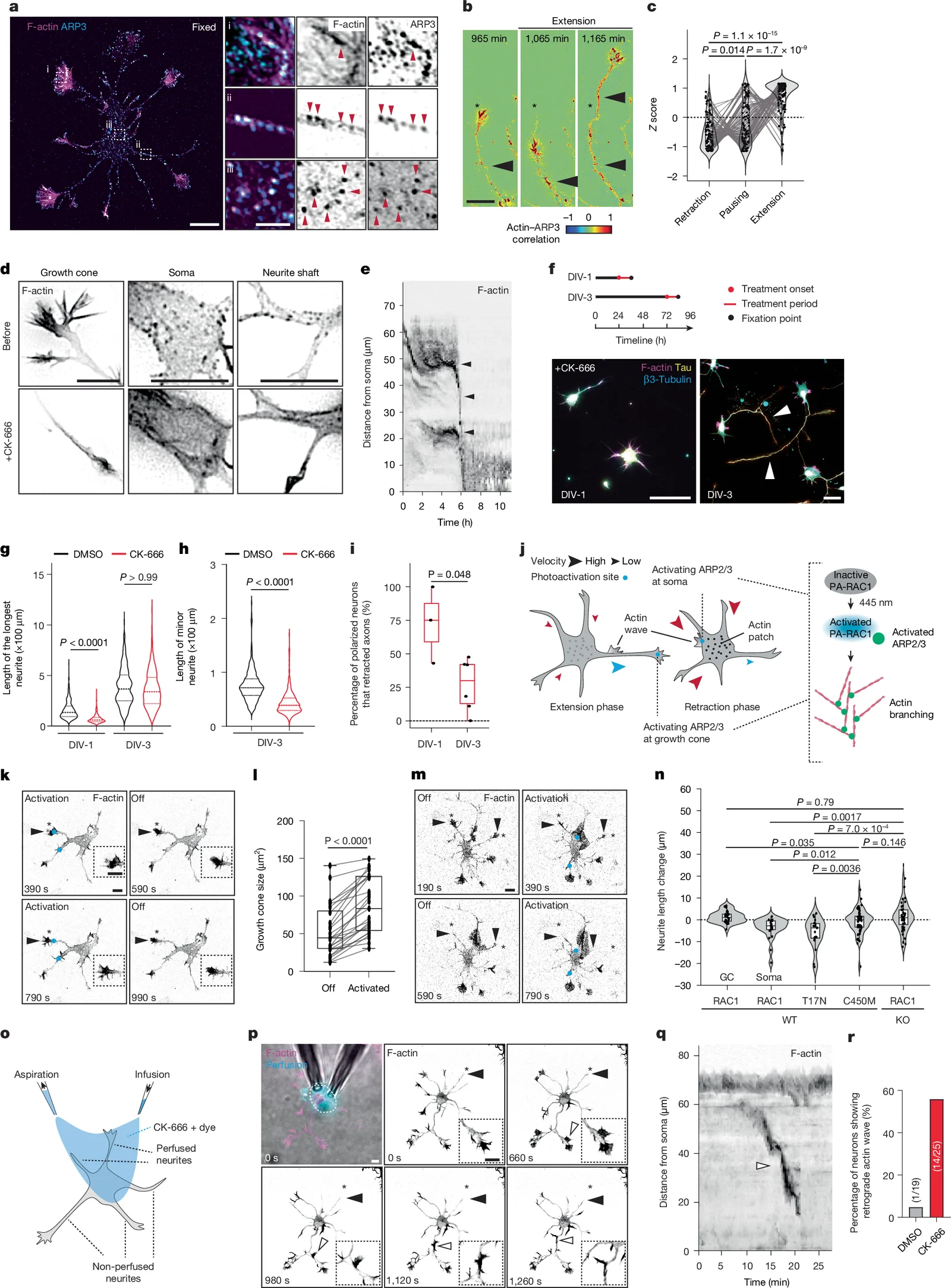
The ARP2/3 complex coordinates oscillatory neurite growth through location-dependent control of growth. **a**, Image of a DIV-1 neuron stained for F-actin (phalloidin, magenta) and ARP3 (cyan). Insets show the growth cone (i), the neurite shaft (ii) and the soma (iii). Red arrowheads point to the ARP3–actin patches. Neurons of three independent experiments recapitulated the results. Scale bar, 10 μm (whole neuron) or 2 μm (insets). **b**, Heatmap of actin–ARP3 colocalization along the extending neurite. Asterisks indicate the starting position of the tip, whereas black arrowheads indicate the sites showing changes in ARP3–actin abundance. Scale bar, 10 μm. **c**, Normalized ARP3 intensities as *Z* scores at extending, pausing and retracting neurites of individual neurons. *n* = 99 neurons from 4 experiments. Two-tailed paired Wilcoxon test with Holm correction for multiple comparisons. **d**, Magnified regions of a polarized DIV-1 neuron before and after 150 µM CK-666 treatment. Neurons of three independent experiments recapitulated the results. Scale bars, 10 μm. **e**, Kymograph of neurite retraction induced by 150 µM CK-666 treatment. Black arrowheads indicate the moving trace of actin condensates from the collapsed growth cone and the branch along the neurite. **f**, Top, schematic of the experimental design. Bottom, images of WT DIV-1 and DIV-3 neurons treated with 200 μM CK-666 for 12 h. Cells were stained for F-actin (phalloidin, magenta), β3-tubulin (cyan) and the axon marker tau (yellow). White arrowheads point to the axons. Neurons of three independent experiments recapitulated the results. Scale bars, 50 μm. **g**, ARP2/3 inhibition via CK-666 retracts axons of DIV-1 neurons but not axons of DIV-3 neurons. *n* = 373 (DMSO-treated) and 322 (CK-666-treated) DIV-1 neurons, and 374 (DMSO-treated) and 322 (CK-666-treated) DIV-3 neurons from 3 experiments. Kruskal–Wallis test followed by Dunn’s post-hoc test with Bonferroni correction. **h**, ARP2/3 inhibition with CK-666 retracts minor neurites of DIV-3 neurons. *n* = 352 (DMSO-treated) and 310 (CK-666-treated) neurons from 3 experiments. Two-tailed Mann–Whitney test. **i**, Percentage of DIV-1 and DIV-3 polarized neurons of which axons were retracted owing to CK-666 treatment. Data are from three independent experiments of DIV-1 neurons and six independent experiments of DIV-3 neurons treated with CK-666. Two-tailed *t*-test. **j**, Schematic of actin-oscillatory growth coupling and the experimental design of local ARP2/3 activation with PA-RAC1. PA-RAC1 photoactivated with a 445-nm laser activates ARP2/3, which nucleates branched actin networks. **k**, Images depicting activation of PA-RAC1 at the growth cone of a DIV-1 neuron. The Lifeact channel (F-actin) of two activation events are shown. Off, removal of light activation. Cyan dots, the activated sites; asterisk, the starting position of the tip; black arrowhead, the extending neurite after activation. Insets, the extending growth cone. Scale bars, 10 μm. **l**, Growth cone enlargement is induced by activation at the growth cone. *n* = 25 activation events on 10 growth cones of 5 neurons, from 4 experiments. Off, before activation. Two-tailed paired Wilcoxon test. **m**, Images depicting activation of PA-RAC1 at the soma cortex of a DIV-1 neuron. Cyan dots, activated sites; asterisk, the starting position of the tips; black arrowhead, retracting neurites adjacent to the emerging actin wave after activation. Scale bar, 10 μm. **n**, Neurite length changes induced by the activation of PA-RAC1 and its variants. For PA-RAC1 in WT neurons, *n* = 31 growth-cone (GC) activation events and 22 soma activation events from 4 experiments; for PA-RAC1(T17N) in WT neurons, *n* = 20 activation events from 3 experiments; for PA-RAC1(C450M) in WT neurons, *n* = 57 activation events from 5 experiments; for PA-RAC1 in *Actr3* KO neurons, *n* = 40 activation events from 3 experiments. Two-tailed Mann–Whitney test. **o**, Schematic of local perfusion with two glass capillaries. The infusion capillary is filled with 150 μM CK-666 mixed with the fluorescent dye FastGreen. The adjacent aspiration capillary restricts the diffusion of CK-666 by removing the infused solution. **p**, Representative serial images of a neuron with local perfusion of 150 μM CK-666 at the soma. White dashed area, the perfusion area; insets, the retrograde moving actin wave; black arrowhead, the retracting neurite; asterisk, the starting position of the neurite tip; white arrowheads, an actin wave that retrogradely translocated from the growth cone towards the soma. A total of 14 out of 25 independent experiments recapitulated the results. Scale bars, 10 μm. **q**, Kymograph of a retrogradely moving actin wave from a growth cone outside the perfusion area. Scale bar, 10 μm. White arrowhead, trace of a retrogradely moving wave. **r**, Percentage of neurons perfused with DMSO or CK-666 showing retrograde actin waves. For the box (**i**,**l**), violin (**g**,**h**) and box-violin plots (**c**,**n**), the centre line is the median, the box and upper and lower lines in the violin show 25th–75th percentiles and whiskers are 1.5× the IQR. The violin spans minimum to maximum values. Exact *P* values are indicated. Source data

**Extended Data Fig. 3:**
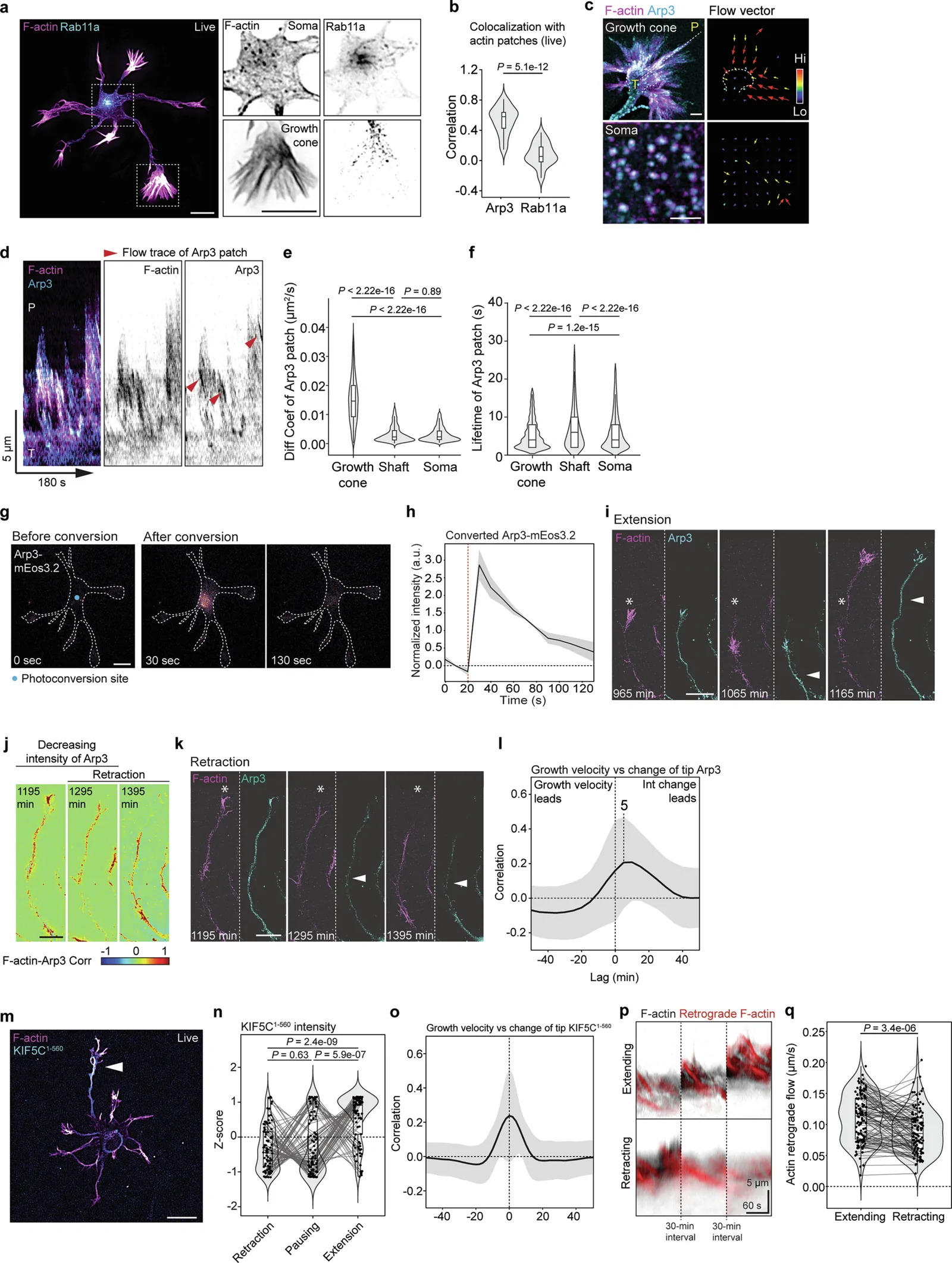
An increase in local ARP2/3-actin patches precedes neurite extension. a. Image of a DIV-1 neuron expressing Lifeact-mScarlet and RAB11A-EGFP. Insets show the soma and the growth cone. Scale bars, 10 μm. b. Colocalization of actin patches with ARP3 and RAB11A. *n* = 41 neurons from 4 independent experiments for ARP3-actin colocalization and 34 neurons from 3 independent experiments for RAB11A-actin colocalization. Two-tailed t-test. c. Left: temporal maximum intensity projection with composite channels. White dashed line: the periphery (P) -transition (T) axis where the kymograph in **d** was derived from. Right: the ARP3 patch flow vector maps. Arrows: local flow direction; color gradient: relative flow rates. Neurons of 3 independent experiments recapitulated the results. Scale bars, 2 μm. d. Kymograph of actin and ARP3 patch flow along the periphery (P) -transition (T) axis at the growth cone shown in **c**. Red arrowheads: the Arp3 patch moving traces. Neurons of 3 independent experiments recapitulated the results. e. Moving velocity of the ARP3 patches. *n* = 10319 growth-cone patches, 3379 neurite-shaft patches and 7312 somatic patches from time-lapsed images of 12 neurons from 3 independent experiments. Kruskal-Wallis test followed by Dunn’s post-hoc test with Bonferroni correction. f. ARP3 patch lifetime. *n* = 2490 growth-cone patches, 1203 neurite-shaft patches and 2206 somatic patches from time-lapsed images of 12 neurons from 3 independent experiments. Kruskal-Wallis test followed by Dunn’s post-hoc test with Bonferroni correction. g. Selected frames of an unpolarized neuron expressing ARP3-mEos3.2 before and after photoconversion. Scale bar, 10 μm. h. Normalized intensity profile of photo-converted ARP3-mEos3.2. *n* = 19 DIV-1 neurons. The red dashed line marks the time point of photoconversion. Values are plotted as mean ± s.e.m. i. The corresponding frames showing an extending neurite in Fig. 3b with separate F-actin and Arp3 channels. Asterisks: the starting position of neurite tip; white arrowheads: the region showing increasing ARP3 along the extending neurite. Scale bar, 10 μm. j. Actin-ARP3 colocalization heatmap along a retracting neurite. Scale bar, 10 μm. k. The corresponding frames in **j** with separated actin and ARP3 channels. Asterisks: the starting position of neurite tip; white arrowheads: the region showing decreasing ARP3 along the retracting neurite. Scale bar, 10 μm. l. Cross-correlation between growth velocity and the change of ARP3 at individual neurite tip. Values are plotted as mean ± s.d. *n* = 213 neurites of 32 neurons from 3 independent experiments. m. Image of a DIV-2 neuron expressing Lifeact-mScarlet and KIF5C^1−560^-mNeonGreen. White arrowhead points to the neurite transiently acquiring KIF5C^1−560^. 3 independent experiments recapitulated the results. Scale bar, 20 μm. n. Normalized KIF5C^1−560^-mNeonGreen intensities as Z scores at the extending, the pausing and the retracting neurites of individual neurons. *n* = 92 neurons from 3 experiments. Two-tailed paired Wilcoxon test with Holm correction. o. Cross-correlation between growth velocity and the change of KIF5C^1−560^ at individual neurite tip. Values are plotted as mean ± s.d. *n* = 550 neurites of 92 neurons from 3 independent experiments. p. Kymographs of actin flow at the extending and the retracting neurite tips. Red channel: traces of retrograde actin flow. The three imaging sessions were spaced by a 30-min interval. q. Retrograde actin flow rates at the neurite tips of extending and retracting neurites. *n* = 119 neurons from 3 experiments. Two-tailed paired t-test. Box-violin plots (**b**, **e-f**, **n**, **q**): center line, median; box, 25th–75th percentiles; whiskers, 1.5 × IQR. Violin spans min to max. Exact *P* values are indicated.

We examined the distribution of ARP2/3 in developing neurons. Immunocytochemistry revealed that ARP3, a subunit of the ARP2/3 complex, was present not only in the growth cone^30,31^ but also in discrete actin-rich patches in the soma and along neurite shafts (Fig. 3a). These ARP3-positive patches did not colocalize with endosomes marked by Ras-associated binding protein 11a (RAB11A)^32^ (Extended Data Fig. 3a,b). Overexpression of fluorescently tagged ARP3 showed that somatic patches were largely stationary, which was in contrast to the retrograde flow of ARP3 patches in growth cones (Extended Data Fig. 3c–e and Supplementary Video 7). Somatic ARP3 patches were short-lived, with lifetimes of less than 10 s (Extended Data Fig. 3f). Consistently, photoconverted somatic ARP3–mEos3.2 rapidly diffused away within 2 min (Extended Data Fig. 3g,h), a result that demonstrates the rapid turnover of ARP3 in these patches.

We then determined how neurite growth correlates with ARP3 distribution. Extending neurites showed elevated ARP3 levels that colocalized with actin (Fig. 3b,c and Extended Data Fig. 3i–k), and increases in ARP3 intensity at the neurite tip preceded acceleration of extension (Extended Data Fig. 3l and Supplementary Video 8). Consistent with previous work^19,33,34^, extending neurites were also enriched in an exogenously expressed truncated kinesin KIF5C construct, a marker of nascent axons^19^ (Extended Data Fig. 3m–o). Moreover, these neurites exhibited faster actin retrograde flow at growth cones than during retraction (Extended Data Fig. 3p,q). Together, these observations support the idea that ARP2/3 activity promotes neurite extension during transient polarization.

## ARP2/3 inhibition causes neurite retraction

**Extended Data Fig. 4:**
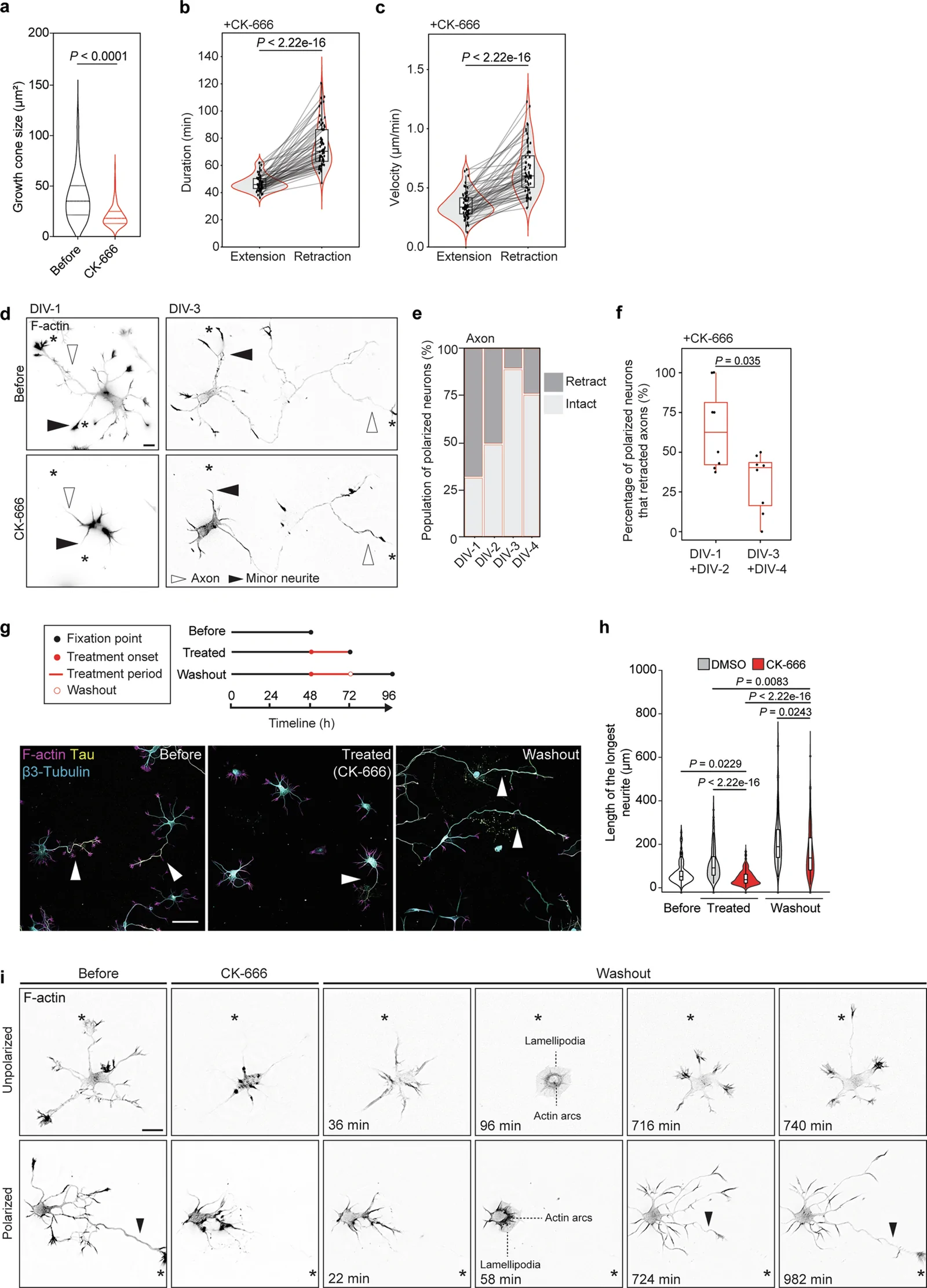
Axons resist CK-666-induced retraction with maturation. a. Growth cone size following 150 μM CK-666 treatment. Before-treatment (Before): *n* = 270 growth cones from 36 neurons; after-treatment (CK-666): 269 growth cones from 47 neurons. Data points are from 3 experiments. Two-tailed t-test. b. Neurite extension and retraction duration following CK-666 treatment. *n* = 60 neurons from 3 independent experiments. Two-tailed paired t-test. c. Neurite extension and retraction velocities following CK-666 treatment. *n* = 63 neurons from 3 independent experiments. Two-tailed paired t-test. d. Selected frames of WT DIV-1 and DIV-3 polarized neurons expressing Lifeact-mScarlet (F-actin) before and during 150 μM CK-666 treatment. The last frame before the treatment (Before), and the last frames after the treatment are shown. White arrowhead: axon; black arrowhead: selected minor neurite; asterisk: the beginning position of the tip. Scale bar, 10 μm. e. Stacked bar plot summarizing the occurrence of retracted axon and neuronal maturation. *n* = 127 polarized neurons from 6 experiments. Two-tailed chi-square test: χ² = 21.783 (df = 3), *P* = 7.24e-05. f. The percentage of polarized neurons of which axons were retracted by CK-666. *n* = 8 batches of DIV-1 or DIV-2 and 9 batches of DIV-3 or DIV-4 neurons treated with CK-666. Two-tailed t-test. g. Top: scheme of experimental design; bottom: Images of DIV-2 WT neurons before, and after 24 h of 150 μM CK-666, and 24 h after washout of CK-666. Fixed neurons were stained for F-actin (phalloidin, magenta), β3-tubulin (cyan), and tau (yellow). White arrowheads: the axons. Scale bar, 10 μm. h. The longest neurite length of neurons treated with CK-666 or control (DMSO) and after washout. Data point number *n* represents the number of neurons from 3 independent experiments: before treatment (Before), 98; DMSO treated, 146; CK-666 treated, 161; washout from DMSO, 68; washout from CK666, 124. Kruskal-Wallis test followed by Dunn’s post-hoc test with Bonferroni correction. i. Living DIV-2 unpolarized and polarized neurons before, after 150 μM CK-666 treatment and after washout of CK-666. Asterisks: the beginning position of the tip; black arrowheads: the axon. 3 independent experiments recapitulated the results. Scale bar, 20 μm. Violin (**a**), box-violin (**b**,** c**, **h**) and box plots (**f**): center line, median; upper/lower lines of violin and box, 25th–75th percentiles; whiskers, 1.5 × IQR. Violin spans min to max. Exact *P* values are indicated.

To test the role of ARP2/3 in neurite growth, we treated neurons with CK-666, a pharmacological ARP2/3 inhibitor^35^. CK-666 caused growth cone collapse, and the normally punctate actin patches reorganized into fibrous networks (Fig. 3d and Extended Data Fig. 4a). These networks merged into condensates that moved towards the soma (Fig. 3e and Supplementary Video 9) and neurites underwent severe retraction within hours (Fig. 3e, Extended Data Fig. 4b,c and Supplementary Video 9). By contrast, in polarized neurons, nascent axons, which no longer received actin waves, progressively became resistant to CK-666-induced retraction, with resistance increasing from 37 ± 26% at 1 and 2 days in vitro (DIV-1/2) to 70 ± 18% at DIV-3/4 (mean ± s.d.; Fig. 3f–i, Extended Data Fig. 4d–f and Supplementary Video 10). Notably, minor neurites of polarized neurons, which continued to receive waves, remained sensitive to CK-666 and retracted after treatment (Fig. 3h).

To test whether CK-666-treated neurons could regrow their neurites and repolarize after recovering actin branching, we evaluated outgrowth following drug wash out. Recovery of ARP2/3 activity triggered robust neurite regrowth (Extended Data Fig. 4g,h). Time-lapse imaging showed that before regrowth, the actin condensates reorganized into lamellipodia and actin arcs at the soma periphery (Extended Data Fig. 4i and Supplementary Video 11). This reorganization was accompanied by a transient retraction of the remnant neurites followed by their regrowth in most cells (81%; Extended Data Fig. 4i and Supplementary Video 11). Notably, the original polarity was largely preserved after recovery (Extended Data Fig. 4i and Supplementary Video 12). That is, among polarized neurons that had retracted their axons, 58% regrew the original axon, 10% formed a new axon and 32% did not regrow an axon. Together, these findings indicate that ARP2/3 activity is essential to counteract a contractile, global inhibitory force that otherwise retracts neurites and suppresses polarization. Once the polarity is established, the maintenance of polarity is independent of ARP2/3.

## ARP2/3 activation affects neurite growth

**Extended Data Fig. 5:**
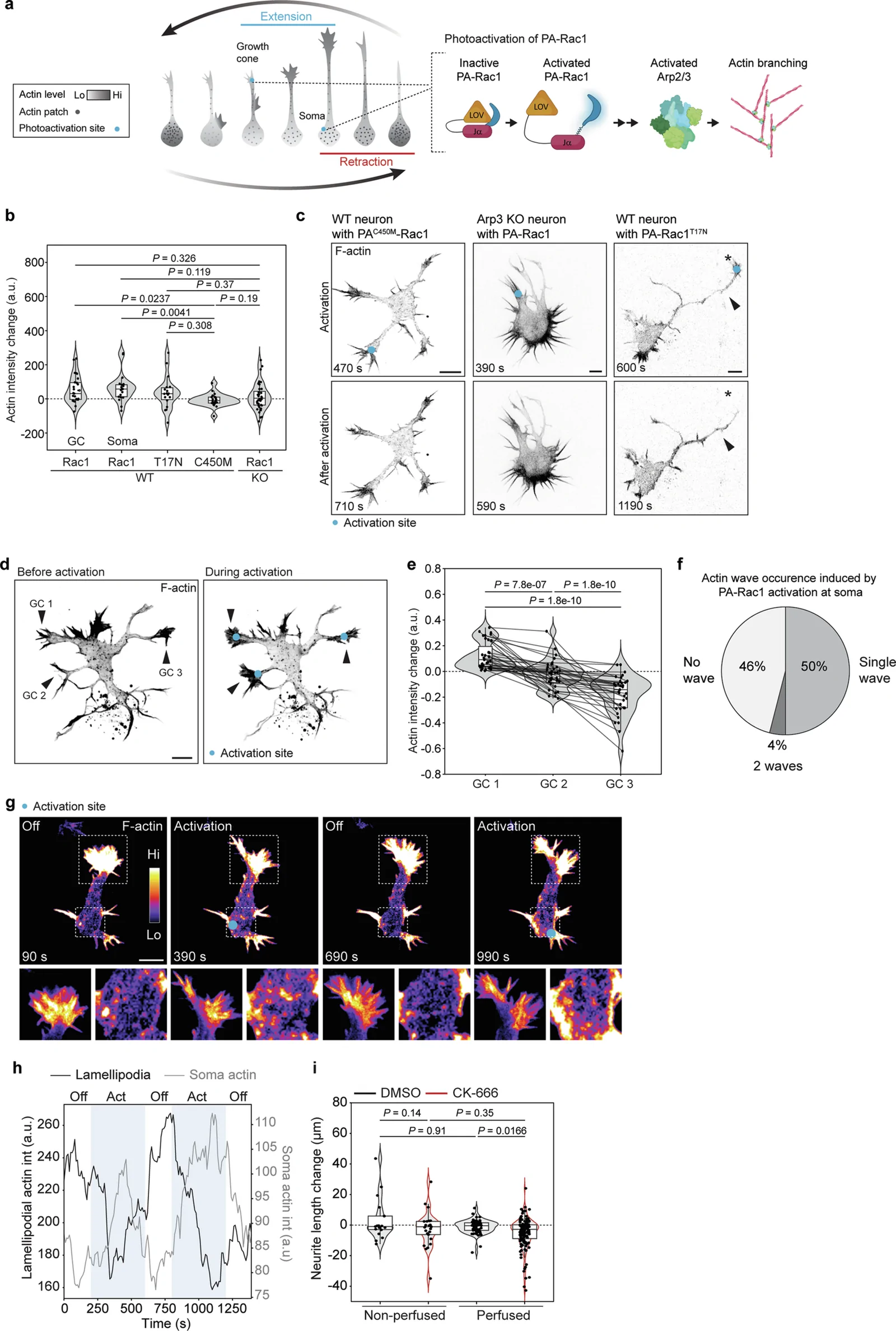
Local activation of ARP2/3 complex remodels actin networks. a. Schematic of local ARP2/3 activation with PA-RAC1. b. Actin intensity difference induced by the activation of PA-RAC1 and the variants. For PA-RAC1 in WT neurons, *n* = 27 activation events at the growth cone (GC) and 21 activation events at the soma; for PA-RAC^T17N^ in WT neurons, *n* = 18 activation events from 3 experiments; for PA-RAC1^C450M^ in WT neurons, *n* = 21 activation events from 5 experiments; for PA-Rac1 in *Actr3* KO neurons, *n* = 40 activation events from 3 experiments. Two-tailed Mann-Whitney test. c. Left: photoactivation of light-insensitive variant PA^C450M^-RAC1 in a WT neuron. Middle: photoactivation of WT PA-RAC1 in an *Actr3* KO neuron. Right: photoactivation of a dominant-negative variant PA-RAC1^T17N^ in a WT neuron. Cyan dots: the activated sites; black arrowhead: the retracting neurite; asterisk: the beginning tip position. Scale bars, 10 μm. d. Images of an unpolarized neuron before and during simultaneous PA-RAC1 activation at multiple growth cones (GCs). Cyan dots: activated sites; black arrowheads: growth cones that were in activation. Scale bar, 10 μm. e. Actin intensity difference in the multiple growth cones (GCs) under PA-RAC1 activation. *n* = 35 neurons from 5 experiments. Two-tailed paired Wilcoxon tests with Holm correction. f. Actin wave occurrence following PA-RAC1 activation at the soma cortex of WT neurons. *n* = 50 activation events of 25 neurons from 12 independent experiments. g. Selected frames of a WT neuron following PA-RAC1 activation. F-actin (Lifeact) is shown. Cyan dots show the activated sites. Note the contrast of zoom-in of soma and lamellipodia was optimized for local actin intensities. 2 independent experiments recapitulated the results. Scale bar, 10 μm. h. Temporal profiles of actin intensity at the soma and at the lamellipodia. The blue shade marks the activation periods. i. Length changes upon micro-perfusion of CK-666 at the soma. Perfused: neurites that were covered by the perfusion area; non-perfused: neurites that sat outside of perfusion area. For DMSO control, *n* = 22 non-perfused neurites and 73 perfused neurites of 15 neurons. For CK-666, *n* = 38 non-perfused neurites and 112 perfused neurites of 23 neurons. Two-tailed Mann-Whitney tests. Box-violin plots (**b**, **e**, **i**): center line, median; box, 25th–75th percentiles; whiskers, 1.5 × IQR. Violin spans min to max. Exact *P* values are indicated. Schematic in **a** created in BioRender; Lin, D. https://biorender.com/pzgxrhg (2026).

Our results showed that lamellipodia and actin waves at the soma are associated with neurite retraction, whereas waves that reach neurite tips are associated with extension. We therefore examined how ARP2/3 activity influences growth in a location-dependent manner. As RAC1 is a physiological upstream regulator of ARP2/3 in developing neurons^36^, we used photoactivatable RAC1 (PA-RAC1)^37^ and mutant variants to locally manipulate ARP2/3 activity (Fig. 3j and Extended Data Fig. 5a). Activation of PA-RAC1 increased local actin assembly, an effect absent in its light-insensitive mutant (PA-RAC1(C450M)) and in neurons in which ARP3 was depleted (Extended Data Fig. 5b,c). Activation of PA-RAC1 at the neurite tip generated an active growth cone that extended the neurite (Fig. 3k,l,n and Supplementary Video 13). Conversely, activation of a dominant-negative PA-RAC1 mutant (PA-RAC1(T17N)) collapsed the growth cone and triggered retraction (Fig. 3n, Extended Data Fig. 5c and Supplementary Video 14).

The cellular response to PA-RAC1 activation was limited to one or a maximum of two sites. Simultaneous activation at three growth cones increased actin assembly in only one or two of the cones, whereas the third showed reduced actin levels (Extended Data Fig. 5d,e and Supplementary Video 15). Notably, activation of PA-RAC1 at the soma generated a lamellipodium that resembled a nascent actin wave (Fig. 3m, Extended Data Fig. 5f and Supplementary Videos 16 and 17). As in native neurons, in which somatic patch intensity decreased when a wave formed (Fig. 2b–d), the PA-RAC1-induced lamellipodium similarly reduced somatic actin patch intensity (Extended Data Fig. 5g,h and Supplementary Video 16). Recapitulating the response in native neurons and the effects of CK-666 washout, the PA-RAC1-induced wave at the soma caused retraction of neighbouring neurites (Fig. 3m,n and Supplementary Video 17). The wave then moved away from the soma, followed by regrowth of the retracted neurite (Supplementary Video 17). Together, these results suggest that ARP2/3 mediates actin-wave-driven oscillatory growth of neurites.

Conversely, locally inactivating ARP2/3 by perfusing CK-666 induced spatially asymmetric effects (Fig. 3o–r and Supplementary Video 18). When CK-666 was perfused over the soma and short neurites, these neurites retracted, whereas the non-perfused neurites or those in vehicle-treated controls did not (Extended Data Fig. 5i). Notably, wave initiation depended on local ARP2/3 activity and was not restricted to the soma. In more than half of the neurons, a neurite located outside the perfused area generated retrograde actin waves originating from its growth cone (14 out of 25 versus 1 out of 19 in dimethyl sulfoxide (DMSO)-treated controls; Fig. 3p–r). These waves propagated towards the soma but dissipated after reaching the CK-666-exposed region.

Taken together, these results demonstrate that ARP2/3 exerts location-dependent control over neurite behaviour. ARP2/3 activity at the soma initiates actin waves and neurite retraction, whereas ARP2/3 activity at neurite tips expands the growth cone and promotes neurite extension.

## ARP2/3 opposes actomyosin in neurite growth

ARP2/3-driven actin waves act as a local activator to promote neurite extension. Therefore, we next examined what provides the global inhibitory mechanism that retracts other neurites after ARP2/3 activation. Because the actin motor myosin II drives neurite retraction^38,39^ and restrains neurite growth^40,41^, we proposed that myosin II mediates this global inhibition. Moreover, interactions between ARP2/3 and myosin II together create the local-activation, global-inhibition system that underlies neuronal polarization.

**Extended Data Fig. 6:**
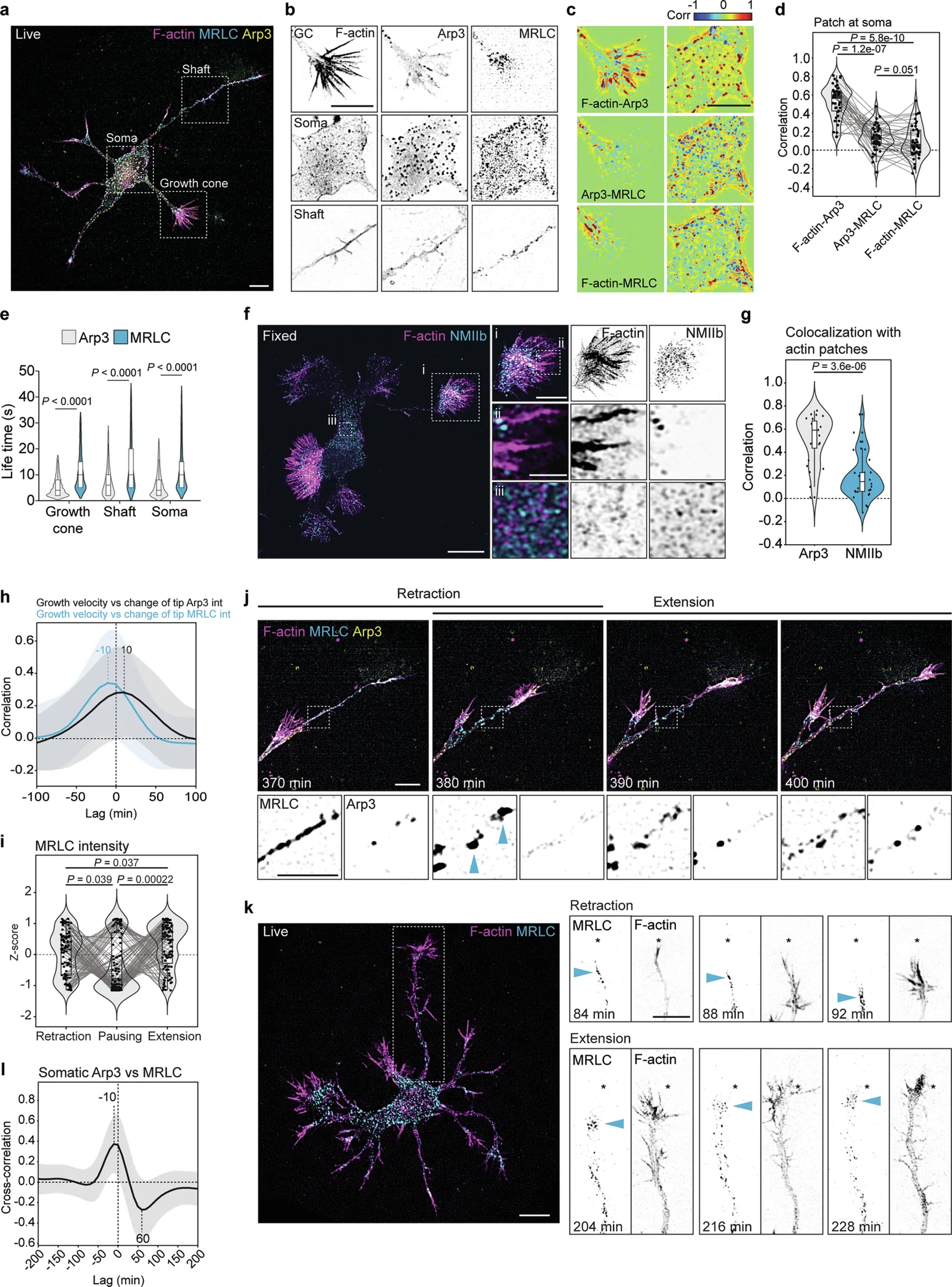
Myosin II and ARP2/3 complex assemble spatially discrete actin networks. a. Selected frame of a DIV-1 neuron expressing Lifeact-mScarlet (F-actin), ARP3-SNAP-SiR (silicon rhodamine) and MRLC-mNeonGreen. Insets mark the regions shown in **b**. Neurons of 4 independent experiments recapitulated the results. Scale bar, 10 μm. b. Lifeact-mScarlet (F-actin), ARP3-SNAP-SiR (ARP3) and MRLC-mNeonGreen (MRLC) in growth cone (GC), soma and neurite shaft from **a**. Scale bar, 10 μm. c. Colocalization heatmaps of the actin-ARP3, ARP3-MRLC and actin-MRLC spatial correlation at the growth cone and the soma from **b**. Scale bar, 10 μm. d. Colocalization of indicated proteins at the soma. *n* = 82 neurons from 4 independent experiments. Two-tailed paired Wilcoxon test with Holm correction. e. ARP3 and MRLC patch lifetime. For Arp3 patches: *n* = 10319 for patch number at the growth cones, 3379 at the neurite shafts and 7312 at the soma from time-lapsed images of 12 neurons from 3 independent experiments. The data source is the same as in Extended Data Fig. 3f. For MRLC patches: *n* = 2995 at the growth cones, 3920 at the neurite shafts and 2703 at the soma from time-lapsed images of 11 neurons from 3 independent experiments. Two-tailed Mann-Whitney test. f. Images of WT neurons stained for F-actin (phalloidin, magenta) and myosin IIb (NMIIb, cyan). Insets show the growth cone (i) and (ii), and the soma (iii). Neurons of 3 independent experiments recapitulated the results. Scale bar of the whole neuron, 10 μm; inset (i), 5 μm; insets (ii) and (iii), 2 μm. g. Colocalization of actin patches with ARP3 and NMIIb patches. *n* = 24 neurons for ARP3 and 31 neurons for NMIIb from 3 independent experiments. Two-tailed Mann-Whitney test. h. Cross-correlation of neurite growth velocity with the changes of ARP3 and MRLC intensities in the individual neurite tips of WT neurons. The peaks are labeled with the lagged times. Values are plotted as mean ± s.d. *n* = 721 neurites of 99 neurons from 4 independent experiments. i. Normalized MRLC intensities as Z scores at the extending, the pausing and the retracting neurites of individual neurons. *n* = 99 neurons from 4 independent experiments. Two-tailed paired Wilcoxon test with Holm correction. j. Selected frames of a neurite in retraction and in extension. The neurite is of the same neuron as in **a**. Scale bar, 10 μm. Insets mark the region of neurite shaft. Blue arrowhead: the MRLC condensates in the retracting neurite. Neurons of 4 independent experiments recapitulated the results. Scale bar, 5 μm. k. Left: image of a neuron expressing Lifeact-mScarlet (F-actin) and MRLC-mNeonGreen. White dashed box: one selected neurite showing extension and retraction. Right: selected frames of the selected neurite in retraction and in extension. Blue arrowheads: the MRLC condensation in the neurite shaft in retraction and MRLC dispersion at the growth cone in extension; asterisk: the beginning position of tip. Neurons of 3 independent experiments recapitulated the results. Scale bars, 10 μm. l. Cross-correlation between changes of ARP3 and MRLC intensities at the soma. The correlation peaks and valleys are labeled with the lagged times. Values are plotted as mean ± s.d. Data points are of the same source as in **h**. Box-violin plots (**d-e**, **g**, **i**): center line, median; box, 25th–75th percentiles; whiskers, 1.5 × IQR. Violin spans min to max. *P* values are indicated.

We first examined myosin II localization. Co-expression of fluorescently tagged myosin regulatory light chain (MRLC) and ARP3 showed that the two proteins form adjacent but non-overlapping patches in the soma and neurite shafts (Extended Data Fig. 6a–d and Supplementary Video 19), with myosin II patches persisting twice as long as ARP3 patches (Extended Data Fig. 6e). Immunocytochemistry confirmed this complementary spatial pattern for endogenous ARP3 and myosin IIb, the prevalent cortical myosin II isoform^42^ (Fig. 3a and Extended Data Fig. 6f,g).

Cross-correlation analysis revealed the temporal relationship between the two networks. MRLC accumulated at the growing neurite tip around 10 min after growth acceleration and about 20 min after ARP3 enrichment (Extended Data Fig. 6h). However, these increases in MRLC levels were subtle, as extending and retracting neurites showed similar overall MRLC intensities (Extended Data Fig. 6i). Instead, neurite retraction was associated with condensation of MRLC patches along the retracting neurites (Extended Data Fig. 6j,k). Conversely, extension was associated with dispersion of MRLC patches at the growth cone (Extended Data Fig. 6k and Supplementary Videos 20 and 21). At the soma, MRLC accumulation also followed ARP3 recruitment (Extended Data Fig. 6l). Together, these findings indicate that myosin II levels follow ARP2/3 activity, and that actomyosin networks are more stable and long-lived than the rapidly turning-over ARP2/3-mediated actin branching process.

**Extended Data Fig. 7:**
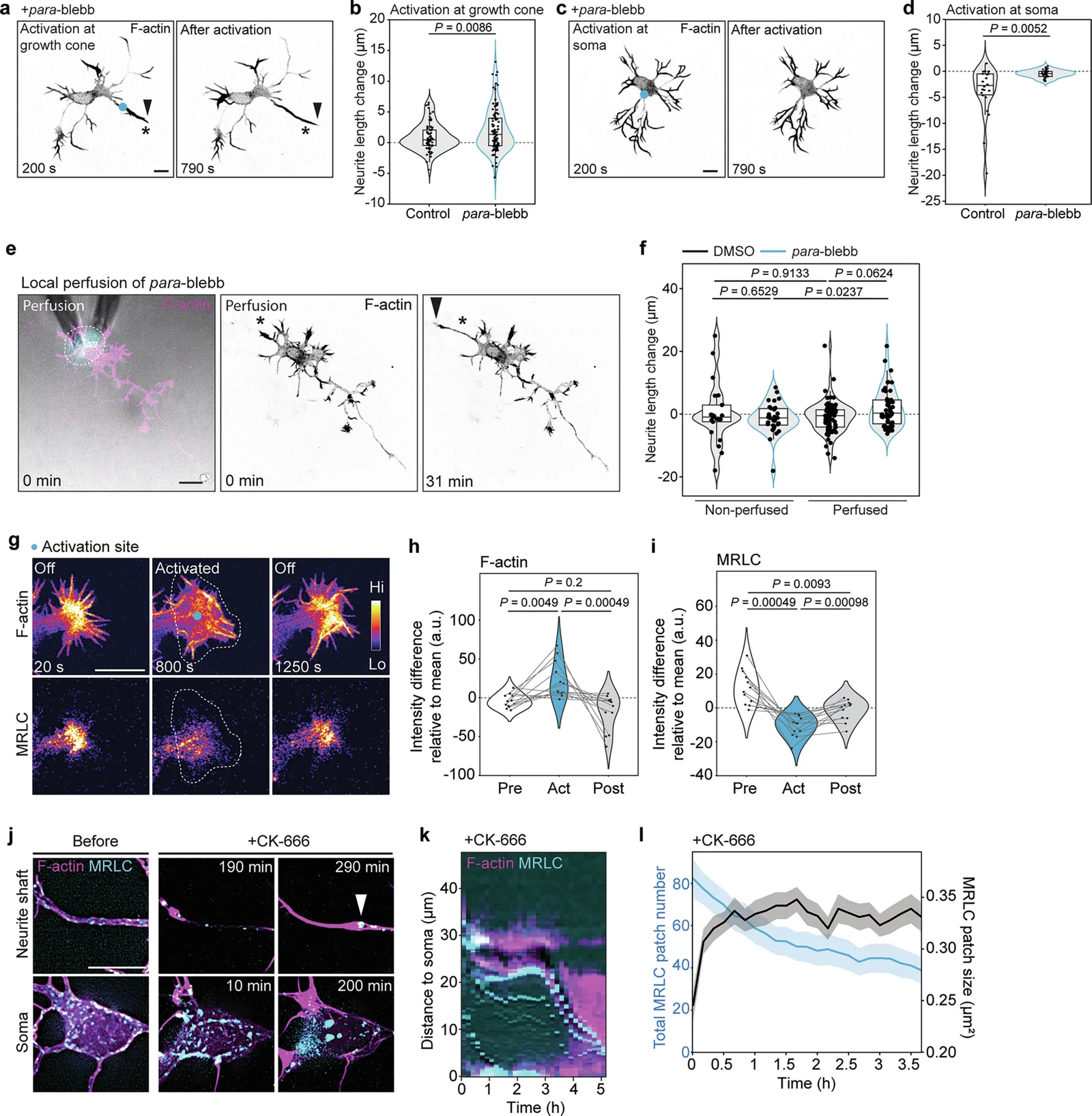
ARP2/3 antagonizes the assembly and contraction of actomyosin networks. a. Photoactivation of PA-RAC1 at the growth cone of a WT neuron treated with 40 μM *para*-aminoblebbistatin. Cyan dot: activated site; black arrowhead: the neurite in extension; asterisk: the beginning position of tip. Scale bar, 10 μm. b. Neurite length changes induced by the activation of PA-RAC1 at the growth cone treated with 40 μM *para*-aminoblebbistatin. *n* = 68 neurites of 15 control cells and 104 neurites of 58 treated cells. Two-tailed Mann-Whitney test. c. Photoactivation of PA-RAC1 at the soma of a WT neuron treated with 40 μM *para*-aminoblebbistatin. Cyan dot: activated site. Scale bar, 10 μm. d. Neurite length changes induced by the activation of PA-RAC1 at the soma treated with 40 μM *para*-aminoblebbistatin. Control: same group of PA-RAC1 at the growth cone as in Fig. 3n. *n* = 22 cells of control and 33 of treated cells. Two-tailed Mann-Whitney test. e. Representative serial images of a neuron with local perfusion of 40 μM *para*-aminoblebbistatin at the soma. White dashed line marks the perfusion area. Asterisk: the beginning position of the tip. Black arrowhead: the extending neurite. Scale bar, 20 μm. f. Neurite length changes upon local perfusion of 40 μM *para*-aminoblebbistatin at the soma. Perfused: neurites that were covered by the perfusion area; non-perfused: neurites that located outside of perfusion area. For DMSO control, *n* = 21 non-perfused neurites and 73 perfused neurites of 15 neurons. For *para*-aminoblebbistatin, *n* = 30 non-perfused neurites and 49 perfused neurites of 16 neurons. Two-tailed Mann-Whitney test. g. Selected frames of a cell expressing Lifeact-mScarlet (F-actin) and MRLC-SNAP-SiR (MRLC) following PA-RAC1 activation. Cyan dot: the activated region. White dashed line: the quantified area. Scale bar, 10 μm. h. Actin intensity difference relative to mean at the growth cones before (Pre), during (Act) and after (Post) PA-RAC1 activation. *n* = 12 activation events from 5 independent experiments. Two-tailed paired Wilcoxon test with Holm correction. i. The same as in **h**, but showing MRLC intensity. j. Selected frames of the soma and the neurite shaft of a WT neuron expressing Lifeact-mScarlet (F-actin, magenta) and MRLC (cyan) before and after 150 μM CK-666 treatment. White arrowhead: actomyosin condensate. 3 independent experiments recapitulated the results. Scale bar, 10 μm. k. Kymograph of F-actin and MRLC along the selected neurite of a CK-666-treated neuron. 62 neurons of 3 independent experiments recapitulated the results. l. MRLC patch size and number following CK-666 treatment. Values are plotted as mean ± s.e.m. *n* = 62 neurons from 3 independent experiments. Box-violin (**b**, **d**, **f**) and violin (**h-i**) plots: center line, median; box, 25th–75th percentiles; whiskers, 1.5 × IQR. Violin spans min to max. Exact *P* values are indicated.

Consistent with the idea that global myosin II activity restrains neurite extension and drives retraction following ARP2/3 activation, inhibiting myosin II with *para*-aminoblebbistatin enhanced the extension induced by PA-RAC1 activation at the neurite tip (Extended Data Fig. 7a,b). Moreover, *para*-aminoblebbistatin treatment abolished the retraction triggered by PA-RAC1 activation at the soma (Extended Data Fig. 7c,d). Consistently, local perfusion of *para*-aminoblebbistatin at the soma and short neurites induced neurite extension (Extended Data Fig. 7e,f).

We then examined how myosin II might be regulated by ARP2/3. Consistent with the view that actin branching disrupts contraction of antiparallel actomyosin networks^43–45^ and competes with actin bundling factors for actin binding^46,47^, activation of ARP2/3 by PA-RAC1 at actomyosin arcs caused the arcs to disassemble (Extended Data Fig. 7g–i and Supplementary Video 22). This effect was transient, with arcs reassembling within 10 min after photoactivation (Extended Data Fig. 7g,i). Conversely, inhibition of ARP2/3 with CK-666 treatment globally across the whole cell led to condensation of MRLC patches that colocalized with actin condensates and moved towards the soma during neurite retraction (Extended Data Fig. 7j–l and Supplementary Video 23). Similarly, neurite retraction correlated with MRLC patch condensation, a result that recapitulated the MRLC condensation found in retracting neurites in native cells (Extended Data Fig. 6j,k). Taken together, the results show that ARP2/3 antagonizes myosin II activity at neurite shafts and tips and prevents neurite retraction.

## ARP2/3 is required for neuronal polarization

**Fig. 4:**
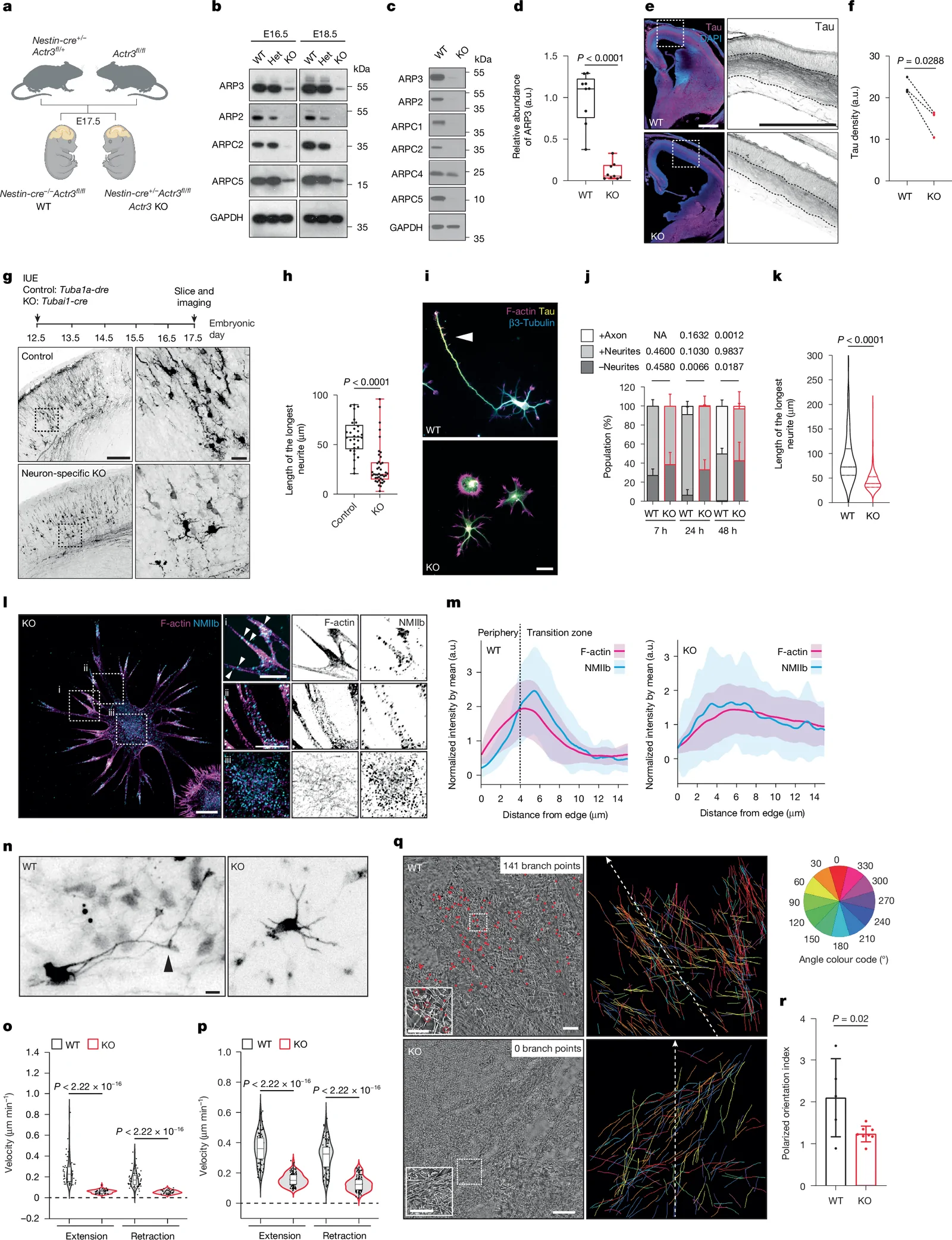
Axonal growth requires the ARP2/3 complex to counteract the dominance of actomyosin. **a**, Breeding scheme for obtaining CNS-specific *Actr3* KO mouse embryos. **b**, Western blot of intact cortex extracts from WT and *Actr3* KO mice for analysis of subunits of the ARP2/3 complex. Het, heterozygous deletion of *Actr3* (*Nestin-cre*^*+/−*^*Actr3*^*fl/WT*^). Loading control, GAPDH. **c**, Western blot of ARP2/3 subunits in cultured cortical neuron extracts. **d**, Quantification of ARP3 levels in **c**. *n* = 9 samples analysed in 3 experiments. Two-tailed *t*-test. **e**, Neocortical coronal transections of paraformaldehyde-fixed E17.5 brains show staining of axons with the marker tau (magenta) and nuclei with DAPI (cyan). Insets, cortical layers showing tau-positive axonal tracts. Dashed area, axon tracts. *n *= 3 WT and 3 *Actr3* KO sections recapitulated the results. Scale bars, 500 μm. **f**, Quantification of tau density. *n* = 3 WT and 3 *Actr3* KO sections from three independent experiments. Two-tailed paired Wilcoxon test. **g**, Top, schematic of the experimental design of IUE for neuron-specific *Actr3* KO. Bottom, images of E17.5 neocortical coronal transections of paraformaldehyde-fixed *Actr3*^*fl/−*^ brain samples with neuron-specific expression of the indicated DNA recombinases. Cre generates *Actr3* KO, whereas Dre is the control. The expression of LYN(TM)–mNeonGreen depended on the recombinase. Scale bars, 100 μm or 20 μm (insets). **h**, Quantification of the length of the longest neurites of neurons in **g**. *n* = 30 control neurons expressing the Dre recombinase and 42 *Actr3* KO neurons expressing the Cre recombinase from 3 acute cortical slices of 3 independent brains. Two-tailed Mann–Whitney test. **i**, Images of WT and *Actr3* KO neurons grown on poly-l-lysine (PLL)-coated surface, stained for F-actin (phalloidin, magenta), β3-tubulin (cyan) and the axon marker tau (yellow) 48 h after plating. Scale bar, 20 μm.** j**, Percentage of neurons with axons (+Axon), with neurites (+Neurites) and without neurites (–Neurites) at 7 h, 24 h and 48 h after plating. *n* = 4 experiments with WT and 6 experiments with *Actr3* KO. Error bars show the mean ± s.e.m. Two-way analysis of variance (ANOVA) on the ‘with axon’ fraction with Tukey post hoc test. NA, not applicable. **k**, Length of the longest neurites. *n* = 181 WT neurons and 209 *Actr3* KO neurons from 3 experiments. Two-tailed Mann–Whitney test. **l**, Images of *Actr3* KO DIV-1 neurons with neurites. Phalloidin-stained F-actin (magenta) and myosin IIb (NMIIb, cyan) are shown. Insets show neurite tips (i and ii) and soma (iii). White arrowheads, NMIIb patches at filopodia. Three independent experiments recapitulated the results. Scale bars. 10 μm (whole neuron) or 5 μm (insets). **m**, Line profiles of the intensities of phalloidin-stained actin and immunostained myosin IIb with respect to the growth cone leading edge. The dashed line separates peripheral and transition regions. *n* = 44 growth cones of 16 WT neurons and 105 neurite tips of 12 *Actr3* KO neurons. Values are plotted as the mean ± s.d. **n**, Live-cell images of WT and *Actr3* KO neurons in acute cortical slices expressing LYN(TM)–mNeonGreen. Black arrowhead, the axon. Scale bar, 10 μm. **o**, Quantification of extension and retraction velocities of WT and *Actr3* KO neurites in acute cortical slices. *n* = 59 WT and 52 *Actr3* KO neurons from 3 experiments. Two-tailed Mann–Whitney test. **p**, Quantification of extension and retraction velocities of WT and *Actr3* KO neurites in 2D in vitro culture. *n* = 100 WT neurons from 4 experiments and 117 *Actr3* KO neurons from 6 experiments. Two-tailed Mann–Whitney test. **q**, Left, selected computational slices through electron tomographs of actin filaments at the neurite tips. Red dots, branch points. Insets, zoom-in of regions marked by the white dashed boxes. Branching points are marked with red circles. Scale bar, 100 nm. Right, projection of 3D filament model. The colours of the filaments specify their angles relative to the leading edge. The white dashed arrow specifies the direction towards the leading edge. Red filaments are with barbed ends pointing to the leading edge (90°). Scale bars, 200 nm. **r**, Polarized level of the actin filament orientation. *n* = 5 WT tomograms of 4 neurons and 8 *Actr3* KO tomograms of 8 neurons. Antiparallel alignment gives an index value close to one. Error bars show the mean ± s.d., two-tailed *t*-test. For the box (**d**,**h**), violin (**k**) and box-violin plots (**o**,**p**), the centre line is the median, the upper and lower lines in violin and box show 25th–75th percentiles and whiskers are 1.5× the IQR. The violin spans minimum to maximum values. Exact *P* values are indicated. Schematic in **a** created in BioRender; Lin, D. https://biorender.com/pzgxrhg (2026). Source data

Paradoxically, ARP2/3 has been reported to both promote and inhibit axon growth in vitro^29,30^. However, these studies relied on molecular manipulations of ARP2/3 that may have altered localization of the complex, which limits insight into the physiological role of ARP2/3. To determine the physiological requirement of ARP2/3 for neuronal polarization, we used a genetic loss-of-function approach. We generated mouse embryos in which the gene encoding ARP3 (*Actr3*) was deleted in the CNS (*Nestin-cre*^*+/−*^*Actr3*^*fl/fl*^)^48^, hereafter referred to as *Actr3* KO mice (Fig. 4a). These mice were compared with wild-type littermates (*Nestin-cre*^*−/−*^*Actr3*^*fl/fl*^) or separate wild-type embryos (*Nestin-cre*^*−/−*^*Actr3*^*WT/WT*^), collectively termed WT mice. Immunoblotting of cortical tissue and isolated neurons from *Actr3* KO mice showed that homozygous *Actr3* deletion depleted ARP3 and concurrently reduced all other ARP2/3 subunits (Fig. 4b–d).

**Extended Data Fig. 8:**
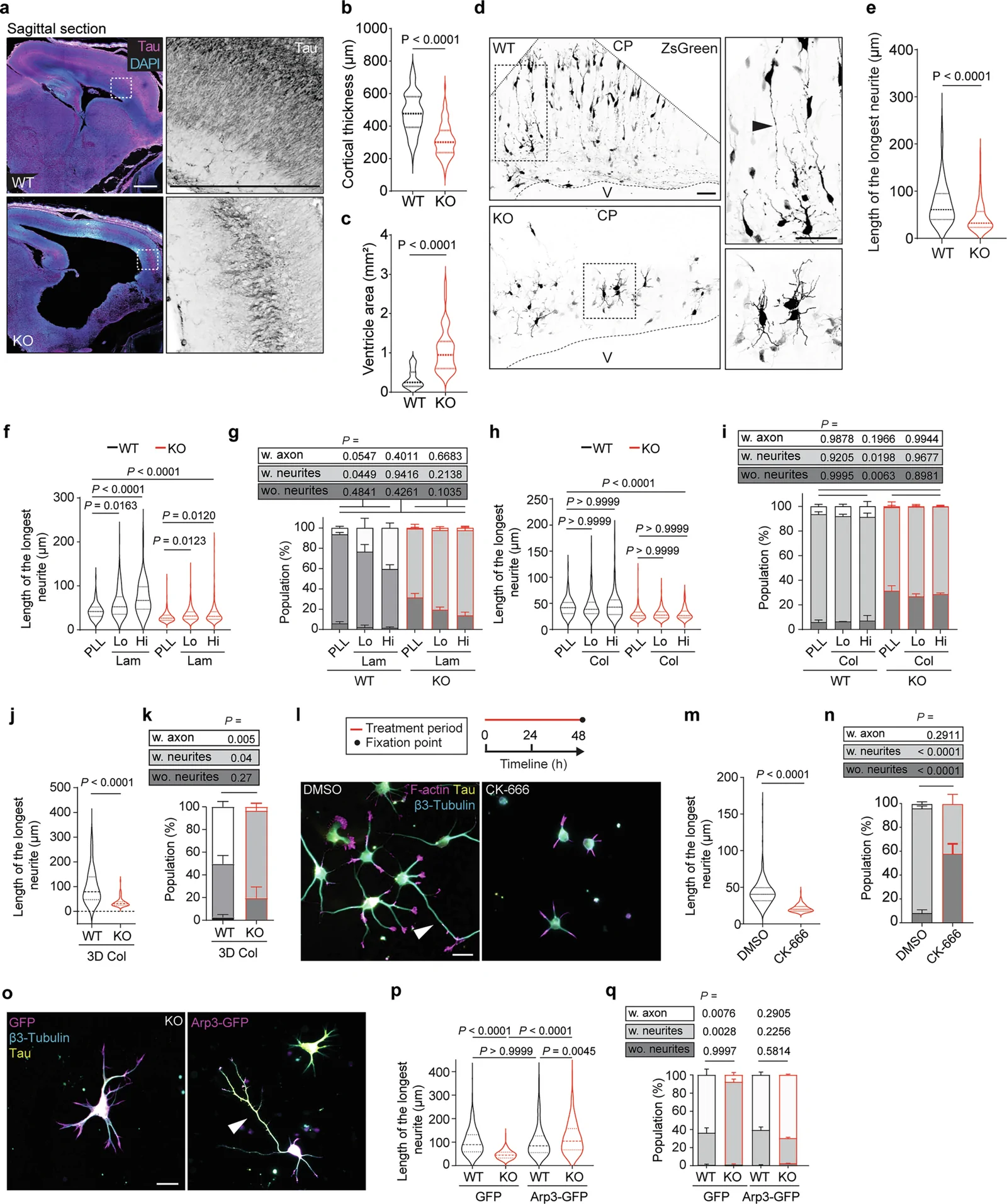
Axon formation requires ARP2/3 complex in vivo and in vitro. a. Left: neocortical sagittal transections of PFA-fixed E17.5 brains show axon marker Tau (magenta) and the nucleus DAPI-staining (cyan). Right: zoom-ins of Tau-positive axonal tracts. 3 independent experiments recapitulated the results. Scale bars, 500 μm. b. Cortical thickness. *n* = 157 regions for WT and 251 regions of interest for *Actr3* KO coronal transections from 3 independent experiments. Two-tailed t-test. c. Ventricle area. *n* = 23 WT and 40 *Actr3* KO coronal sections from 3 independent experiments. Two-tailed t-test. d. Left: images of neocortical coronal transections of WT and *Actr3* KO PFA-fixed E17.5 brains expressing *Zoanthus sp*. green fluorescent protein (ZsGreen). CP, cortical plate; V, ventricle. Right: zoom-ins of selected regions. Black arrowhead: axon. 3 independent experiments recapitulated the results. Scale bars, 50 μm. e. Quantification of length of the longest neurites of the neurons in (d). *n* = 110 WT neurons and 230 *Actr3* KO neurons from 3 slices of 3 independent brains. Dashed lines mark the quantiles and the median. Two-tailed Mann-Whitney test. f. Length of the longest neurites of individual neurons grown on polylysine (PLL) and laminin (Lam). Low (Lo): 5 μg/ml laminin; high (Hi): 25 μg/ml laminin. *n* = 229, 178, 233, 220, 206, 266 neurons for the indicated conditions from 4 (PLL) or 3 (Hi- and Lo-Lam) independent experiments. Kruskal-Wallis test followed by Dunn’s post-hoc test with Bonferroni correction. g. Percentage of neurons with axon (w. axon), with and without neurites (w. and wo. neurites) on PLL or laminin. *n* = 3 (WT) or 4 (*Actr3* KO) independent experiments. Error bar shows mean ± s.e.m. Two-way ANOVA on the “with axon” fraction with Tukey post-hoc test. h. Length of the longest neurites of individual neurons grown on 2D collagen-coated (Col) surface. Lo: 5 μg/ml collagen; Hi: 25 μg/ml collagen. *n* = 229, 173, 169, 220, 188, 196 neurons for the indicated conditions from 3-4 independent experiments. Kruskal-Wallis test followed by Dunn’s post-hoc test with Bonferroni correction. i. Percentage of neurons with axons on 2D PLL- or collagen-coated (Col) surface. *n* = 3 (WT) or 4 (*Actr3* KO) independent experiments. Error bar shows mean ± s.e.m. Two-way ANOVA on the “with axon” fraction with Tukey post-hoc test. j. Length of the longest neurite of individual neurons grown in 3D collagen (3D Col). *n* = 85 WT and 66 *Actr3* KO neurons from 3 independent experiments. Two-tailed Mann-Whitney test. k. Percentage of neurons with axons in 3D collagen (3D Col). *n* = 3 independent experiments. Error bars show mean ± s.e.m. Two-tailed t-test. l. Top: scheme of experimental design; bottom: images of WT neurons treated with DMSO or CK-666 and stained for F-actin (phalloidin, magenta), β3-tubulin (cyan), and tau (yellow). White arrowhead: axon. Scale bar, 20 μm. m. Length of the longest neurite. *n* = 246 DMSO treated neurons and 113 CK-666 treated neurons from 4 independent experiments. Two-tailed Mann-Whitney test. n. Percentage of neurons with axons. *n* = 4 independent experiments. Error bar shows mean ± s.e.m. Two-tailed t-test. o. Images of *Actr3* KO neurons following GFP or ARP3-EGFP transfection and immunostained for GFP (magenta), β3-tubulin (cyan) and tau (yellow). White arrowhead: the axon. Of note, the non-rescued one at the upper right corner is GFP-negative. Scale bar, 20 μm. p. Length of the longest neurites of individual neurons expressing GFP or ARP3-GFP. *n* = 255, 243, 242 and 261 neurons for the indicated conditions from 4 independent experiments. Kruskal-Wallis test followed by Dunn’s post-hoc test with Bonferroni correction. q. Percentage of neurons with axons. Error bar shows mean ± s.e.m of *n* = 4 independent experiments. Two-tailed t-test. Violin plots (**b-c**, **e-f**, **h**, **j**, **m**, **p**): center line, median; upper/lower lines, 25th–75th percentiles. Violin spans min to max. Exact *P* values are indicated.

In addition to corticogenesis defects previously attributed to ARP2/3 loss^49^ (Fig. 4e and Extended Data Fig. 8a–c), we observed a marked reduction in axonal tracts in brain slices from *Actr3* KO mice (Fig. 4e,f and Extended Data Fig. 8a). Individual *Actr3* KO neurons labelled by IUE exhibited impaired axon formation (Extended Data Fig. 8d,e). To test whether such a defect reflects impaired cell-intrinsic polarization, we used the promoter of tubulin-α1 (*Tuba1a*) to drive neuron-specific expression of Cre through IUE. As the *Tuba1a* promotor starts to express later than the Nestin promoter^50,51^, we used brain samples from *Actr3*^*fl/−*^ mice to ensure efficient ARP2/3 depletion. The results consistently showed abrogated axon formation (Fig. 4g,h). Cultured *Actr3* KO neurons also did not develop axons irrespective of 2D or 3D culture environments or substrates (Fig. 4i–k and Extended Data Fig. 8f–k). WT neurons treated with CK-666 immediately after plating recapitulated the *Actr3* KO phenotypes (Extended Data Fig. 8l–n). Conversely, re-expression of ARP3 in *Actr3* KO neurons restored axonal growth to WT levels (Extended Data Fig. 8o–q). Notably, neurons lacking actin depolymerizing factor (ADF) and cofilin did not form neurites at all^52^, a phenotype distinct from *Actr3* KO neurons. Thus, ARP2/3 is specifically required for polarization and not neurite initiation.

## ARP2/3 suppresses actomyosin network dominance

**Extended Data Fig. 9:**
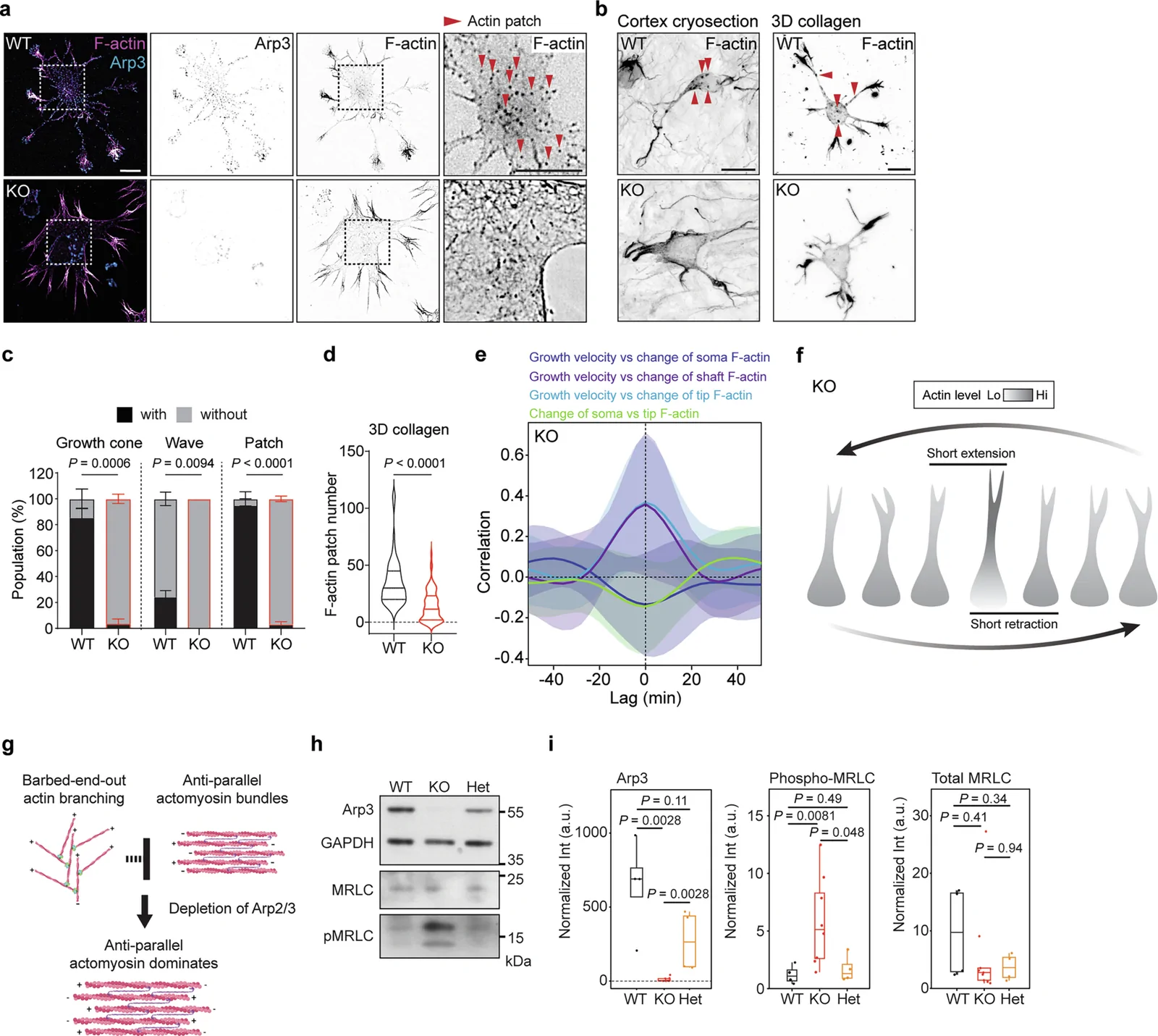
*Actr3* KO abolishes the actin organizations that are associated with axon growth and changes the actin-neurite growth temporal coupling. a. Images of WT and *Actr3* KO neurons stained for F-actin (phalloidin, magenta) and ARP3 (cyan). Red arrowheads: actin patches. Insets: actin organization in the soma. Neurons of 3 independent experiments recapitulated the results. Scale bars, 10 μm. b. Images of WT and *Actr3* KO neurons in E17.5 cortical sections and 3D collagen matrix. Cortical sections were labeled with Lifeact-mScarlet (F-actin) via in utero electroporation at E14.5. The neurons in 3D collagen matrix were stained for F-actin with phalloidin. Red arrowheads: actin patches. Neurons of 3 independent experiments recapitulated the results. Scale bars, 10 μm. c. Percentage of WT and *Actr3* KO cultured neurons with growth cones, actin waves and actin patches. *n* = 3 independent experiments. Error bar shows mean ± s.e.m. Two-tailed t-test. d. Cortical actin patch number in the somas of neurons in 3D collagen. *n* = 75 WT and 74 *Actr3* KO neurons from 3 independent experiments. Data are shown as violin plot spanning the minimum to maximum values (center line, median; the upper and lower lines, 25th–75th percentiles). Two-tailed t-test. e. Cross-correlation of the indicated pairs of temporal profiles. Values are plotted as mean ± s.d. of correlation functions of *n* = 62 neurons from 3 independent experiments. f. Scheme for actin-neurite growth coupling in *Actr3* KO neurons. Shaded arrows indicate the cycling of events. g. Mechanistic model of *Actr3* KO neuron phenotypes. ARP2/3-nucleated actin branching antagonizes the assembly and contraction of antiparallel actomyosin. Thus, the depletion of actin branching increases the dominance of actomyosin. h. Western blot of phospho-MRLC (pMRLC), total MRLC (MRLC) and ARP3 in cultured cortical neuron extracts. Het, heterozygous deletion of *Actr3* (*Nestin-cre*^*+/−*^* Actr3*^*fl/wt*^). Loading control: GAPDH. i. Quantification of ARP3, phospho-MRLC and total MRLC intensities in western blot analysis. *n* = 3 samples per genotype in each of 4 independent experiments. Data are shown as box plot (center line, median; box, 25th–75th percentiles; whiskers, 1.5 × IQR). Two-tailed Mann-Whitney test. Exact *P* values are indicated. Schematic in **g** created in BioRender; Lin, D. https://biorender.com/pzgxrhg (2026).

To understand how the loss of ARP2/3 constrains axon formation, we examined how ARP2/3 depletion reorganizes the actin cytoskeleton. As observed with pharmacological ARP2/3 inhibition (Fig. 3d), growth cones, actin waves and patches were absent in *Actr3* KO neurons (Extended Data Fig. 9a–d and Supplementary Video 24). Consistent with the idea that actin branching drives oscillatory growth, the loss of these ARP2/3-dependent structures was accompanied by a loss of sequential coupling between actin and oscillatory growth. Neurite growth velocity changed instantly with the change of actin intensities (Extended Data Fig. 9e,f). Moreover, both *Actr3* KO cortical slices and cultured *Actr3* KO neurons exhibited a reduction in growth dynamics (Fig. 4n–p and Supplementary Videos 24 and 25).

**Extended Data Fig. 10:**
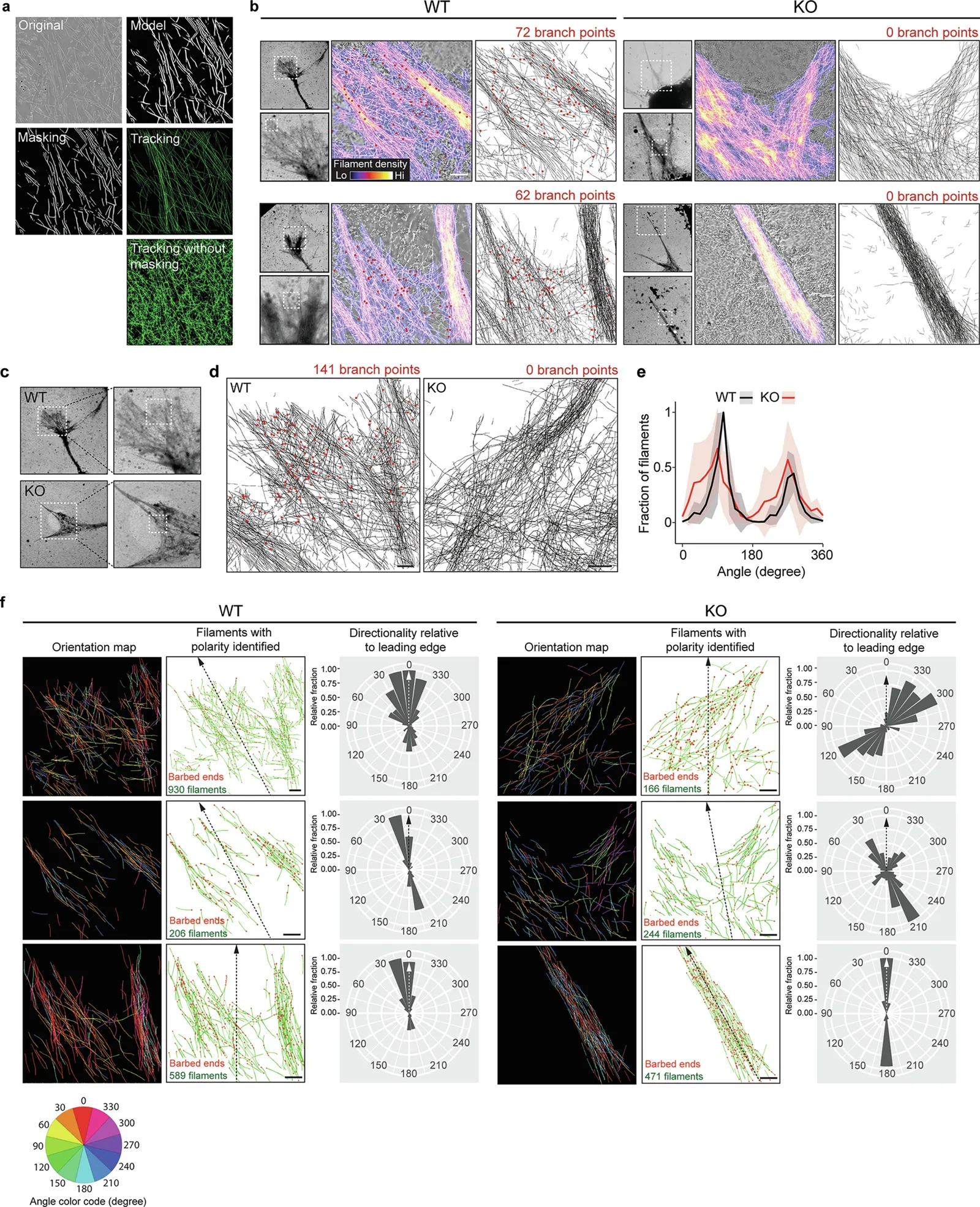
*Actr3* KO abrogates actin branch formation and alters actin geometry. a. Representative images of each step of machine-learning assisted segmentation and automatic tracking of negative-stained EM tomogram of actin filaments. The same steps were applied in all 5 WT and 8 *Actr3* KO tomograms. b. Selected computational slices through electron tomograms of actin filaments. Left: the micrographs of the investigated neurite tips. Middle: computational slices through electron tomograms of actin filaments, overlaid with filament density heatmap and branch points (red dots). Right: the projection of the 3D model of actin filaments (black) with branching points (red dots). Scale bar, 200 nm. c. Electron micrographs of extracted actin at neurite tips, of which tomograms are shown in Fig. 4q. d. Tracked actin filaments (grey) from the tomograms in Fig. 4q are overlaid with branch points (red). Scale bars, 200 nm. Results recapitulating **b-d** were observed in all 5 WT and 8 *Actr3* KO tomograms. e. Orientation distribution of actin filaments. *n* = 5 WT tomograms of 4 neurons and 8 *Actr3* KO tomograms of 8 neurons. Values are plotted as mean ± s.d. f. Left: color-coded orientation map of actin filaments. Red filaments are with barbed ends pointing to the leading edge (0 degree). Middle: model of actin filaments with polarity determined (green). Red dots: barbed ends. The orientation reference line is depicted as the dashed line perpendicular to the leading edge, with the arrowhead pointing to the leading edge. Right: rose plots showing actin filament orientation distribution. The concentric white circles mark the relative filament fraction, with the maximum fraction normalized as one. The orientation reference lines are defined as in the middle panel. Similar results were observed in all 5 WT and 8 *Actr3* KO tomograms. Scale bars, 200 nm.

We proposed that these growth defects arise because without ARP2/3, contractile actomyosin networks take over the cell. If ARP2/3-driven branching counteracts actomyosin networks^45,46,53^, then loss of ARP2/3 should increase myosin II activity (Extended Data Fig. 9g). Indeed, myosin IIb patches in *Actr3* KO neurons expanded into distal areas of neurites (Fig. 4l,m), whereas they were excluded in WT neurons, such as the peripheral domain of the growth cone (Extended Data Fig. 6f). Moreover, myosin II occupied unstructured, entangled fibrous actin networks at the neurite shaft and soma (Fig. 4l). Consistently, analyses of biochemical extracts of *Actr3* KO neurons showed an increase in phospho-MRLC (Extended Data Fig. 9h,i), which reflects higher myosin II activity. Electron tomograms displayed a bipolar orientation at filopodial shafts and interfilopodia junctions in *Actr3* KO neurons, results characteristic of an antiparallel actomyosin network (Fig. 4q,r, Extended Data Fig. 10 and Supplementary Video 26). This architecture contrasted sharply with the unipolar orientation, whereby barbed ends pointed towards the leading edge of WT growth cones. Together, these results suggest that the depletion of ARP2/3 leads to overdominant actomyosin networks that block axon formation.

## ARP2/3 counteracts the actomyosin network

In non-neuronal motile cells, microtubules control the retraction of protrusions^54^. Similarly, microtubules oppose the actomyosin-mediated constraints on axon formation and regeneration^55,56^, and their stability correlates with axon development^56^. Thus, we speculated that the antagonism between ARP2/3 and actomyosin influences microtubule integrity, which in turn determines whether axons and minor neurites resist retraction.

**Extended Data Fig. 11:**
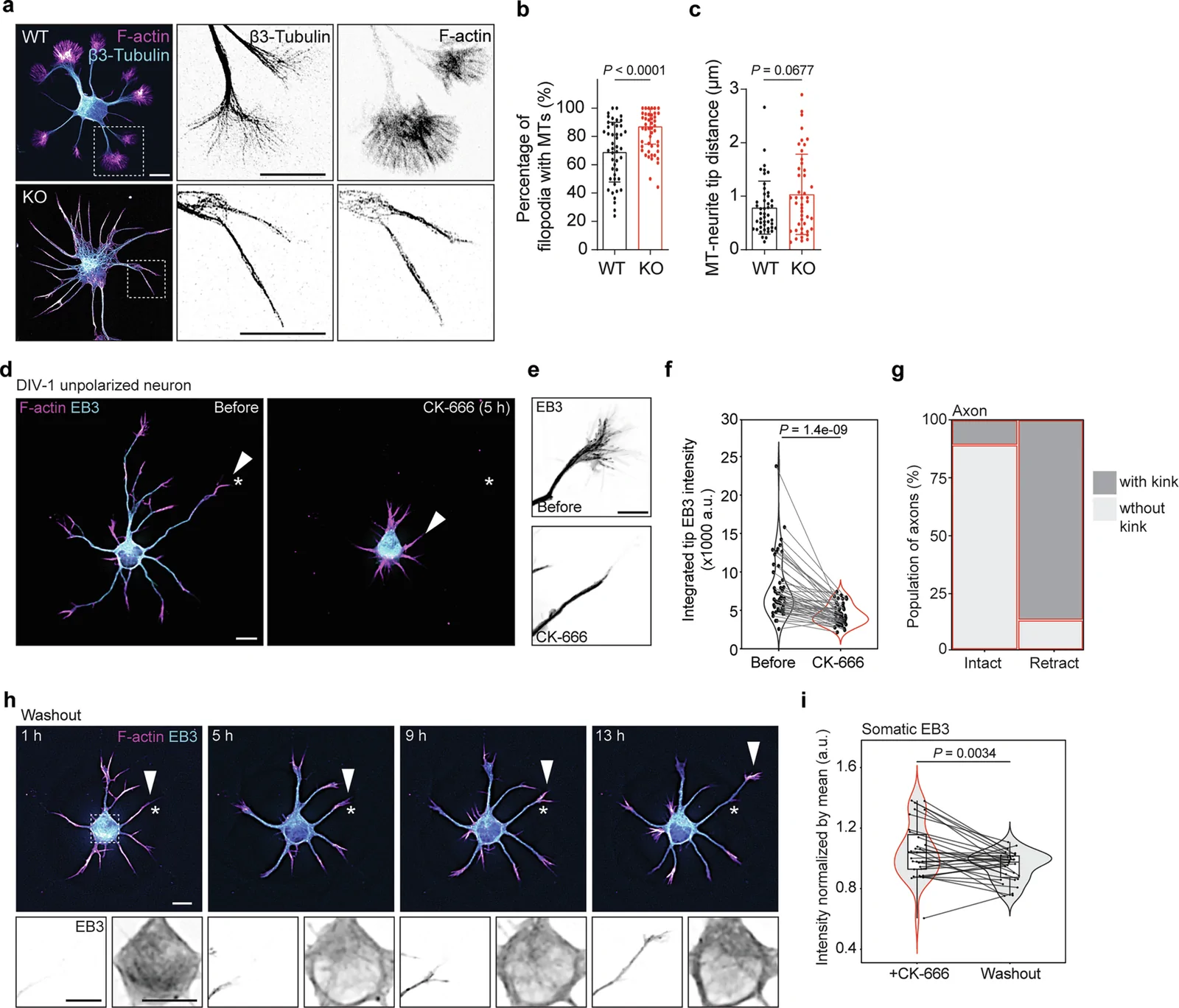
Acute ARP2/3 inhibition with CK-666 induces actomyosin contraction that compresses microtubules and retracts neurites. a. Images of WT and *Actr3* KO neurons at 24 h post-plating, stained for F-actin (phalloidin, magenta) and β3-Tubulin (cyan). Scale bars, 10 μm. b. Percentage of filopodial actin bundles aligned with microtubules (MTs) at the neurite tips. *n* = 46 WT and 46 *Actr3* KO neurons pooled from 3 experiments. Error bar shows mean ± s.d. Two-tailed t-test. c. Distance between microtubule ends to neurite tips. *n* = 46 WT and 45 *Actr3* KO neurons from 3 independent experiments. Error bar shows mean ± s.d. Two-tailed t-test. d. Selected frames of a WT neuron expressing Lifeact-mScarlet (F-actin, magenta) and EB3-mNeonGreen (EB3, cyan) before and after 150 μM CK-666 treatment. White arrowhead: one selected retracting neurite; asterisk: the starting position of the neurite tip. Scale bar, 10 μm. e. Temporal maximum intensity projection of EB3 in the selected retracting neurite tip. 25 frames over 2 min were projected for each condition. Scale bar, 10 μm. f. Integrated EB3 intensity at the neurite tips before and after 150 μM CK-666 treatment. The neurite tip region is defined by the actin protrusion. *n* = 52 neurons from 3 experiments. Two-tailed paired Wilcoxon test. g. Stacked bar plot summarizing the occurrence of microtubule kinks in retracted and non-retracted axons. The tile size corresponds to the frequency of data points within each combination of categories. *n* = 127 polarized neurons from 6 experiments. Two-tailed chi-square test: χ² = 72.024 (df = 1), *P* = 2.13e-17. h. Selected frame of the same WT neuron as in **d** expressing Lifeact-mScarlet (F-actin, magenta) and EB3-mNeonGreen (EB3, cyan) after washout of CK-666. White arrowhead: the selected regrowing neurite; asterisk: the starting position of the neurite tip. Insets show EB3 channel at the soma and at the selected regrowing neurite. Scale bars, 10 μm. i. EB3 intensities at the soma before and after removal of CK-666. *n* = 31 neurons from 3 experiments. Two-tailed paired Wilcoxon test. Violin (**f**) and box-violin (**i**) plots: center line, median; box, 25th–75th percentiles; whiskers, 1.5 × IQR. Violin spans min to max. Exact *P* values are indicated.

We first investigated microtubule organization in *Actr3* KO neurons. Despite the substantial increase in actomyosin networks, microtubules advanced into neurite tips and even aligned with filopodial actin bundles to a greater extent than in WT neurons (Extended Data Fig. 11a–c). These observations suggest that simply advancing microtubules into neurite ends is insufficient to overcome the strong contractile constraint present in *Actr3* KO neurons.

**Fig. 5:**
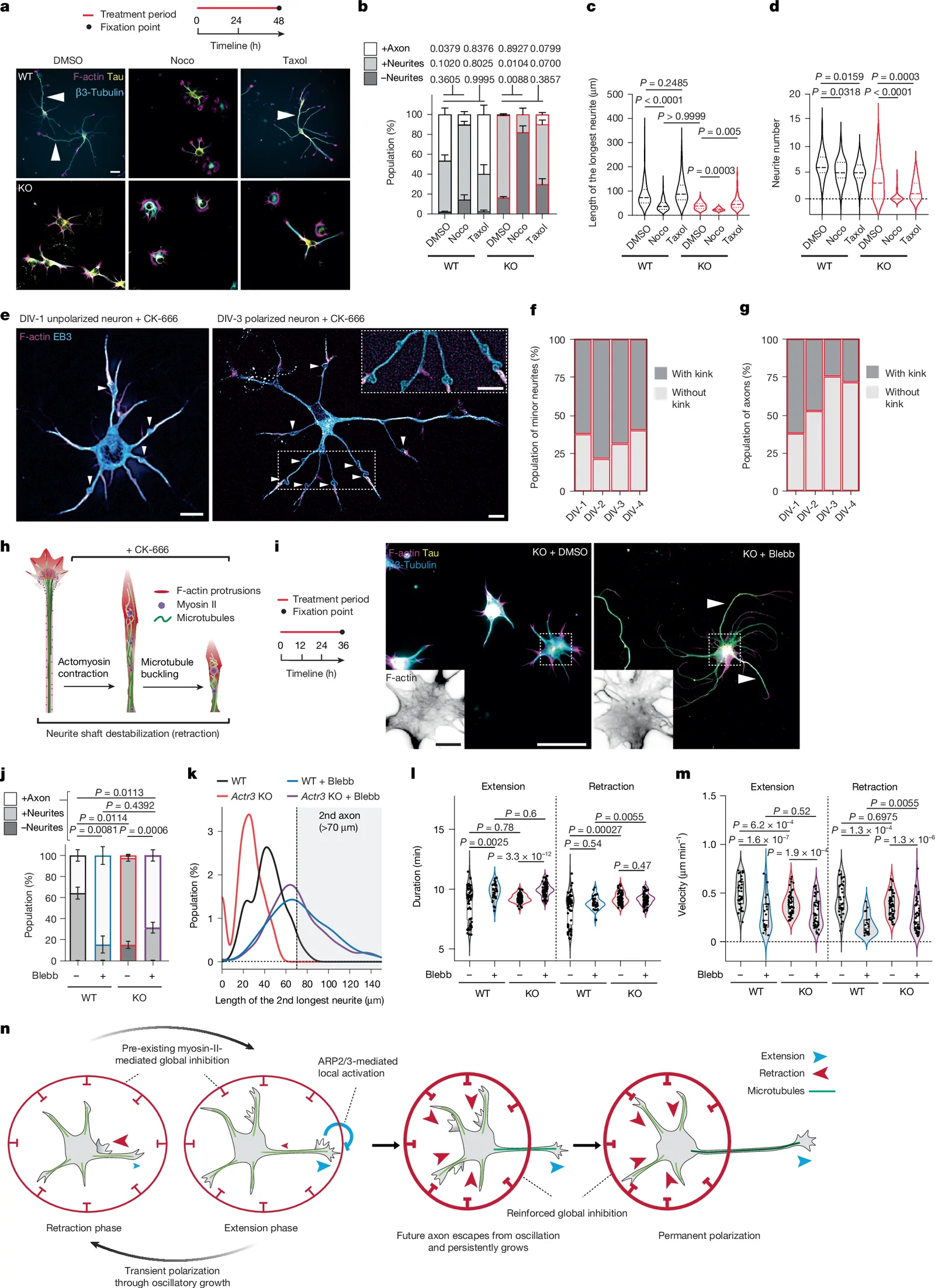
ARP2/3 prevents microtubule buckling and enables persistent growth by antagonizing actomyosin contractility. **a**, Top, experimental design. Bottom, images of neurons treated with DMSO, 75 nM nocodazole (Noco) or 5 nM taxol for 48 h and stained for actin (magenta), β3-tubulin (cyan) and tau (yellow). White arrowhead, the axon. Scale bar, 20 μm. **b**, Percentage of neurons with axon, or with or without neurites at 48 h after plating in polarization stages. *n* = 4 experiments. Error bars show the mean ± s.e.m., two-way ANOVA on the ‘with axon’ fraction with Tukey post hoc. **c**, Length of the longest neurite at 48 h after plating. *n* = the number of neurons from four independent experiments: WT, *n* = 236 (DMSO), 212 (Noco) or 236 (taxol); KO, *n* = 204 (DMSO), 42 (Noco) or 167 (taxol). Kruskal–Wallis test followed by Dunn’s post hoc test with Bonferroni correction. **d**, Number of neurites. *n* = the number of neurons from two independent experiments: WT, *n* = 86 (DMSO), 60 (Noco) or 41 (taxol); KO, *n* = 80 (DMSO), 32 (Noco) or 55 (taxol). Kruskal–Wallis test followed by Dunn’s post-hoc test with Bonferroni correction. **e**, Selected frame of WT DIV-1 and DIV-3 neurons expressing Lifeact–mScarlet (magenta) and EB3–mNeonGreen (cyan) after 150 μM CK-666 treatment. White arrowheads, microtubule kinks. The inset shows microtubule kinks in DIV-3 minor neurites. Scale bars, 10 μm. **f**, Stacked bar plot summarizing the occurrence of microtubule kinks in minor neurites of DIV-1 to DIV-4 neurons. *n* = 16 (DIV-1), 57 (DIV-2), 29 (DIV-3) and 25 (DIV-4) polarized neurons from 6 experiments. Two-tailed chi-square test: *χ*^2^ = 3.83 (d.f. = 3), *P* = 0.28. **g**, Stacked bar plot summarizing the occurrence of microtubule kinks in axons of DIV-1 to DIV-4 neurons. The data source is the same as in **f**. Two-tailed chi-square test: *χ*^2^ = 9.19 (d.f. = 3), *P* = 0.027. **h**, Summary of CK-666-induced cytoskeletal reorganization and neurite retraction. **i**, Left, schematic of the experimental design. Right, images of *Actr3* KO neurons treated with DMSO or 20 μM blebbistatin (Blebb) for 24 h and stained for F-actin (phalloidin, magenta), β3-tubulin (cyan) and tau (yellow). White arrowheads, the axons. The insets show actin structures at the soma. Scale bars, 100 μm or 10 μm (insets). **j**, Percentage of neurons with axons following blebbistatin treatment. *n* = 5 independent experiments. Error bars show the mean ± s.e.m. Two-way ANOVA on the ‘with axon’ fraction with Tukey post hoc test. **k**, Neurite length distribution of the second longest neurite. WT neurons, *n* = 182 control and 197 blebbistatin-treated neurons; *Actr3* KO, *n* = 229 control and 228 blebbistatin-treated neurons from 4 experiments. **l**, Quantification of extension and retraction duration of DIV-1 WT and *Actr3* KO neurons treated with blebbistatin. WT, *n* = 56 control (DMSO) and 34 blebbistatin-treated neurons; *Actr3* KO, *n* = 66 control (DMSO) and 67 blebbistatin-treated neurons from 5 experiments. Two-tailed Mann–Whitney test. **m**, Quantification of extension and retraction velocities of DIV-1 WT and *Actr3* KO neurons treated with blebbistatin. The data source is the same as in **l**. Two-tailed Mann–Whitney test. **n**, Model of neuronal polarization. Pre-existing myosin-II-mediated global inhibition restrains neurite growth and restricts axon number by default. Through cycles of oscillatory growth, ARP2/3-driven actin waves act as a local activation process that antagonizes global inhibition and enables transient extension of a single neurite. The transient relaxation of actomyosin constraint enables microtubule-driven growth, which in turn enables the future axon to resist retraction and escape from oscillation. Once escaped, the axon grows persistently and independently from ARP2/3, and the minor neurites constrained by reinforced actomyosin networks display increased retraction rates. For violin (**c**,**d**) and box-violin plots (**l**,**m**), the centre line is the median, the upper and lower lines of the violin and box show 25th–75th percentiles and the whiskers are 1.5× the IQR. The violin spans minimum to maximum values. Exact *P* values are shown. Source data

Because actin branching opposes actomyosin contractility and prevents neurite retraction, we proposed that unchecked actomyosin networks compress microtubule bundles along neurites, which makes them vulnerable to microtubule destabilization. Consistent with this view, destabilizing microtubules in WT neurons through nocodazole treatment reduced neurite length and abrogated axon formation to levels comparable to vehicle-treated *Actr3* KO neurons (Fig. 5a–c). Nocodazole-treated *Actr3* KO neurons displayed even more severe deficits in neurite formation (Fig. 5d). Conversely, stabilization of microtubules through treatment with taxol, which induces supernumerary axons^56,57^, did not restore axon formation in *Actr3* KO neurons to WT levels (Fig. 5b).

To test the acute impact of losing actin branching on microtubules, we treated neurons with CK-666 and live-imaged EB3-labelled microtubule plus-ends^58^. CK-666 treatment compressed microtubule plus-end tracks (Extended Data Fig. 11d,e), reduced the number of polymerizing ends (Extended Data Fig. 11f) and induced microtubule kinks along neurite shafts that were associated with retraction (Fig. 5e,f and Supplementary Video 27). In polarized neurons, these microtubule kinks were prevalent in minor neurites (Fig. 5e,f). Among polarized neurons undergoing axon retraction, 88% exhibited microtubule kinks in the retracting axon (Extended Data Fig. 11g). Consistent with the greater resistance of mature axons to retract (Fig. 3g–i), axonal microtubule kinks became less frequent with neuronal maturation (Fig. 5g and Supplementary Video 28). Washout of CK-666 mitigated microtubule compression at the soma and restored microtubule advance towards neurite tips, followed by neurite regrowth (Extended Data Fig. 11h,i and Supplementary Video 29). Collectively, these findings indicate that ARP2/3-mediated actin branching prevents compressive buckling of microtubules by counteracting contractile forces along neurites, which enables sustained neurite extension (Fig. 5h).

## ARP2/3 and actomyosin ensure single axon formation

**Extended Data Fig. 12:**
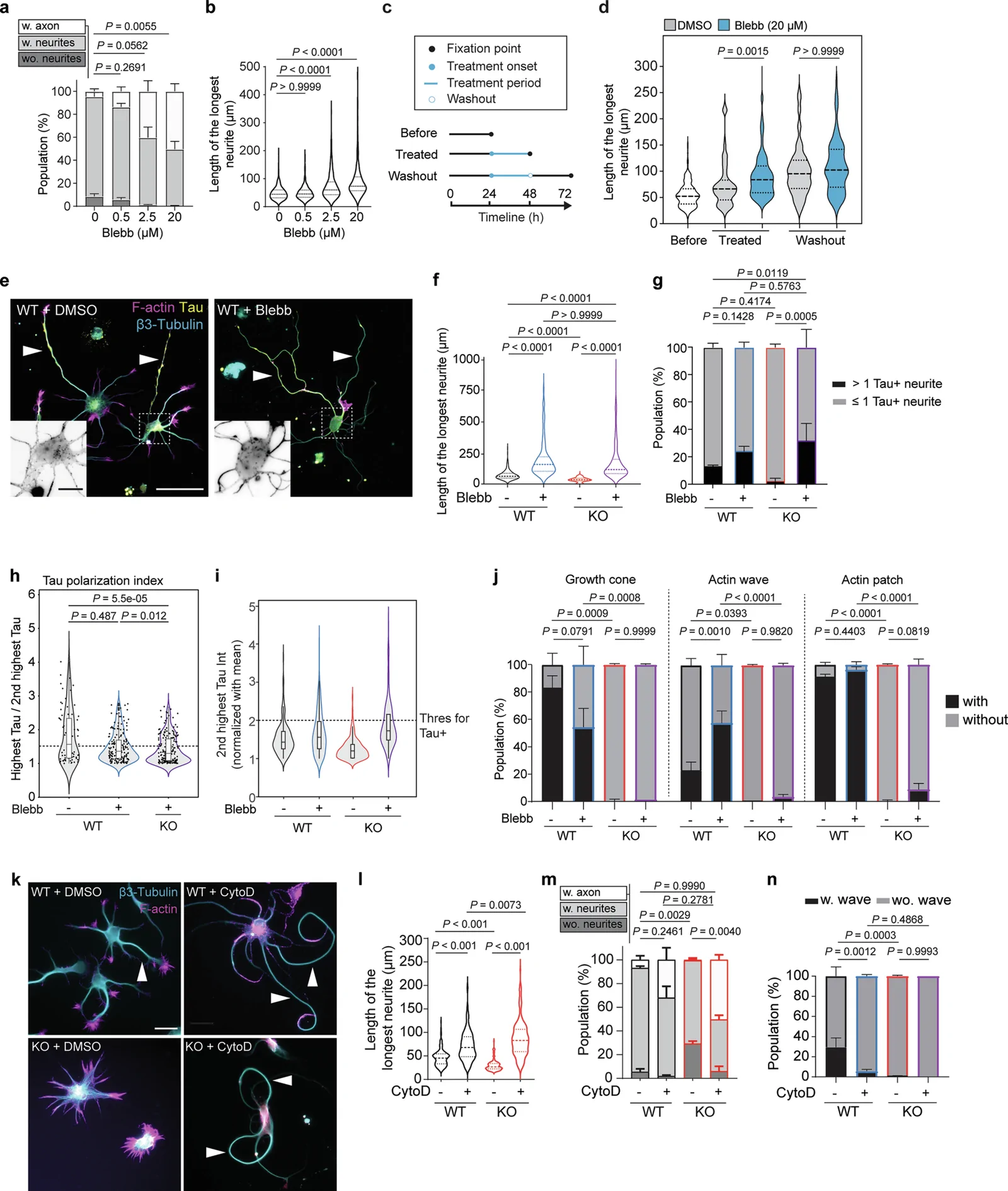
Pharmacological myosin II inhibition induces multiple axon formation in Arp3 KO neurons. a. Percentage of WT neurons with axons, with neurites or without neurites following treatment with blebbistatin (Blebb) of serial dosages: 0 μM, 0.5 μM, 2.5 μM and 20 μM. *n* = 5 experiments. Error bar shows mean ± s.e.m. One-way ANOVA followed by Dunnett’s post-hoc test on “with axon” proportion. b. Length of the longest neurite of WT neurons treated with blebbistatin (Blebb) as in **a**. Data point number *n* represents the number of neurons from 5 independent experiments: 0 μM: 468; 0.5 μM: 298; 2.5 μM: 311; 20 μM: 430. Kruskal-Wallis test followed by Dunn’s post-hoc test with Bonferroni correction. c. Timeline of experimental design for 20-μM blebbistatin treatment and washout. d. Length of the longest neurite of WT neurons treated with blebbistatin (Blebb) or control (DMSO) and after washout. Data point number *n* represents the number of neurons from 3 independent experiments: before treatment (Before), 237; DMSO-treated, 122; blebbistatin-treated, 117; washout from DMSO, 129; washout from blebbistatin, 101. Kruskal-Wallis test followed by Dunn’s post-hoc test with Bonferroni correction. e. Images of WT neurons treated with 20 μM blebbistatin (Blebb) for 24 h and stained for F-actin (phalloidin, magenta), β3-tubulin (cyan), and tau (yellow). The insets show the actin structures at the soma. White arrowheads: the axons. Scale bar, 50 μm; scale bar of insets, 10 μm. f. Length of the longest neurite following blebbistatin (Blebb) treatment. WT: *n* = 371 control (DMSO) and 362 Blebb-treated; *Actr3* KO: *n* = 267 control (DMSO) and 302 Blebb-treated from 5 experiments. Kruskal-Wallis test followed by Dunn’s post-hoc test with Bonferroni correction. g. Percentage of neurons with more than one tau-positive axon. *n* = 3 experiments. Error bar shows mean ± s.e.m. Two-way ANOVA followed by Tukey test. h. Polarization level quantified by asymmetric tau distribution. Neurons with neurite length > 70 μm were analyzed. *n* = 73 DMSO-treated WT, 180 blebbistatin treated WT from 4 independent experiments, and 197 blebbistatin treated *Actr3* KO neurons from 3 independent experiments. The residual 4 DMSO-treated *Actr3* KO neurons with neurite length > 70 μm are excluded from the plot. Kruskal-Wallis test followed by Dunn’s post-hoc test with Bonferroni correction. i. Quantification of the neurites with the second highest tau intensity. The intensity threshold defining Tau-positive axon was two-times of the average intensity across the neurites of a neuron. *n* = 182 DMSO-treated WT, 205 blebbistatin treated WT, 165 DMSO-treated *Actr3* KO and 180 blebbistatin treated *Actr3* KO neurons from 3-4 experiments. j. Percentage of neurons showing growth cone, actin waves, and actin patches. *n* = 4 independent experiments. Two-way ANOVA followed by Tukey test on “with” proportion. k. Images of WT and *Actr3* KO neurons treated with 1 μM cytochalasin D (CytoD) for 24 h and stained for F-actin (phalloidin; magenta) and β3-tubulin (cyan). White arrowheads: the axons. Scale bar, 10 μm. l. Length of the longest neurite following cytochalasin D (CytoD) treatment. WT: *n* = 179 control (DMSO) and 181 cytoD-treated; *Actr3* KO: *n* = 133 control (DMSO) and 185 cytoD-treated from 5 experiments. Kruskal-Wallis test followed by Dunn’s post-hoc test with Bonferroni correction. m.Percentage of neurons with or without axons following cytochalasin D treatment. *n* = 4 independent experiments. Error bar shows mean ± s.e.m. Two-way ANOVA followed by Tukey test on “with axon” proportion. n.Percentage of neurons showing actin waves. *n* = 3 independent experiments. Two-way ANOVA followed by Tukey test on “with actin wave” proportion. Violin (**b**, **d**, **f**, **l**) and box-violin plots (**h, i**): center line, median; upper/lower lines in violin and box, 25th–75th percentiles; whiskers, 1.5 × IQR. Violin spans min to max. Exact *P* values are indicated.

Myosin II imposes a global inhibition effect that constrains axon growth and limits axon number^40,59,60^ (Extended Data Fig. 12a–g). To determine whether ARP2/3 functions primarily by opposing this global inhibition or instead acts as an independent axon determinant, we treated *Actr3* KO neurons with the myosin II inhibitor blebbistatin^40^. If ARP2/3 counteracts myosin II, then blocking myosin II should facilitate supernumerary axon formation in the absence of ARP2/3. If ARP2/3 were an independent determinant, axon formation would remain impaired.

Inhibition of myosin II in *Actr3* KO neurons induced multiple tau-positive axons, with lengths comparable to those of blebbistatin-treated WT neurons (Fig. 5i–k and Extended Data Fig. 12e–i). Growth cones, actin waves and actin patches did not reappear (Extended Data Fig. 12j), which indicated that once myosin II is inactivated, axon growth can proceed independently of ARP2/3. Live-cell imaging further showed that blebbistatin treatment reduced both the extension and the retraction velocities while prolonging extension duration in WT and *Actr3* KO neurons (Fig. 5l,m and Supplementary Video 30). This result suggests that persistent growth—rather than growth speed—drives axon formation.

Similarly, treatment with the actin-depolymerizing drug cytochalasin D induced multiple axon growth in both WT and *Actr3* KO neurons (Extended Data Fig. 12k–n). This result supports the idea that actomyosin networks are the default dominant force in unpolarized neurons and act to restrict axon number. Together, these findings indicate that unchecked myosin II contractility and not the loss of actin branching itself prevents axon formation in *Actr3* KO neurons. Globally releasing this contractile constraint enables persistent growth of multiple neurites and thereby causes supernumerary axons.

## Discussion

During development, millions of neurons must polarize and extend axons to establish functional brain circuits. A long-standing model has proposed that neurites of cortical neurons compete for limiting resources through their growth cones, with the ‘winning’ neurite capturing growth-promoting cues while the others retract^20^. However, the oscillatory growth we observed here is inconsistent with such a simple tug-of-war. Instead, our findings revealed that the soma orchestrates an intrinsic oscillatory program that coordinates neurite behaviour.

Biochemical studies and modelling have suggested that ARP2/3-generated branched actin networks antagonize antiparallel actomyosin bundles^43–45,53,61^. In line with this concept, both pharmacological inhibition of ARP2/3 and genetic loss of ARP3 produced a marked dominance of contractile actomyosin networks that prevented axon growth. Thus, ARP2/3 and actomyosin operate as opposing cytoskeletal systems, the balance of which governs neurite dynamics.

ARP2/3-driven actin branching generates short-lived actin patches and waves^25^. These stochastic actin patches resemble conserved actin-remodelling modules observed across many cell types^26–28^. Notably, activation of ARP2/3 at the soma specifically induced transient retraction before growth resumed. This result probably reflects the proximity of myosin II to ARP2/3 patches at the soma, unlike at growth cones, where myosin II is largely absent from the peripheral domain where ARP2/3 operates.

After the patch intensity decreases at the soma, an actin wave emerges. This wave propagates from the soma into a neurite, probably through self-amplifying ARP2/3 branching at the leading edge of the wave and debranching behind it as actomyosin reassembles^24,62^. When the wave reaches the neurite tip, actin branching transiently relaxes actomyosin-driven constraints, which enables microtubule advance and transforms the tip into a growth cone. This process produces a brief period of axon-like growth. As actomyosin regains dominance, it captures the extending neurite such that the neurite retracts. The cycle then restarts with a new somatic wave entering a different neurite in a stochastic manner to produce oscillatory growth. This mechanism is distinct from neurite initiation itself, which depends on ADF/cofilin-mediated actin turnover^52^.

This oscillatory stage biases future polarization. Stable polarization occurs when one neurite becomes sufficiently resistant to actomyosin-mediated global inhibition and no longer requires ARP2/3-driven waves to grow (Fig. 5n). This neurite then extends persistently without retraction, whereas the remaining neurites are even more restrained by myosin II during and after polarization. Consistent with this idea, reducing myosin II activity facilitates release from global inhibition and in turn promotes axon formation^40^. Although external cues may orientate or accelerate symmetry breaking^63,64^, the global actomyosin-based inhibition seems to be a default, cell-intrinsic property. Such an intrinsic mechanism ensures that only a single neurite develops into an axon, even in an environment rich in growth-promoting molecules. Without such a mechanism, the pervasive axon-guidance ligands and neurotrophic factors would cause supernumerary axons and thereby disrupt brain circuitry.

Once polarity is established through oscillation, polarization is then consolidated through mechanisms that stabilize microtubules^56,65,66^, which is reminiscent of processes that support directional persistence in motile cells^67,68^. It is noteworthy that the clutch mechanism of mesenchymal cell migration^69^, which transduces myosin-driven retrograde actin flow to cell motion, does not seem to apply to growth cones and neurite growth^70^. Indeed, growth cones of embryonic cortical and hippocampal neurons do not pull on 3D soft matrices to move forwards^71^. Thus, the primary function of actomyosin networks in CNS neurons is not to generate traction force but to globally restrain neurite growth. In this process, ARP2/3-nucleated branching locally modulates actomyosin–microtubule interaction rather than directly driving neurite growth via actin protrusions. Consistently, in motile cells, ARP2/3-driven protrusions are dispensable for locomotion but are essential for directed migration^72^.

Together, our findings identify a robust cytoskeletal oscillatory mechanism that ensures reliable neuronal polarization by balancing contractile and branching actin networks. Given that modulation of the actin–microtubule system can promote axon regeneration in the injured adult CNS^41,55,73–75^, understanding how this oscillatory program operates in mature neurons may reveal new therapeutic strategies for CNS repair.

## Methods

### Animals

All the mice handling procedures followed the Animal Welfare Act and the German guidelines of the State Agency for Consumer Protection and Nutrition (LAVE) North Rhine-Westphalia. The animals were housed in groups of up to five in individually ventilated cages from the Tecniplast Green Line. The following parameters were applied: 12-h light–12-h dark cycle (6:00–18:00/18:00–6:00), 22 °C temperature and 60% humidity. For environmental enrichment, nestlets for nest building and red-coloured hiding places (houses, tubes) were provided.

Mice homozygous for the *loxP*-site flanked *Actr3* allele (*Actr3*^*fl/fl*^) were generated as previously described^48,76–78^. To specifically knockout *Actr3* in the CNS, *Actr3*^*fl/fl*^ mice were mated with transgenic Nestin-Cre mice (*Nestin-cre*^*tg/−*^, Jackson Laboratory), in which the Cre recombinase is specifically expressed in the CNS from E12.5 (ref. ^78^). The resulting *Nestin-cre*^*tg/−*^*Actr3*^*fl/WT*^ mice were then backcrossed with the *Actr3*^*fl/fl*^ line. The embryos with the *Nestin-cre*^*tg/−*^*Actr3*^*fl/fl*^ genotype are referred to as *Actr3* KO, and those with the *Nestin-cre*^*−/−*^*Actr3*^*fl/fl*^ genotype or embryos of WT C57BL/6 mice (*Nestin-cre*^*−/−*^*Actr3*^*WT/WT*^) are referred to as WT. The genotypes of all mice used in this study were verified by PCR^77^.

### Primary embryonic neuron culture

The procedure was based on a previously described protocol^79^. Cultured neurons were incubated in a 36.5 °C chamber supplied with 5% CO_2_.

Primary mouse hippocampal neurons were dissected from the brains of E17.5 mice. Tails of embryos were collected for PCR genotyping. Brains from WT and *Actr3* KO mice were separated according to the genotype. Neurons of dissected regions were separated and dissociated in trypsin solution (0.05% trypsin-EDTA, 7 mM HEPES pH 7.3) at 37 °C for 15 min. After washing once in HBSS supplemented with DNase and twice in HBSS, the cells were dissociated in MEM-HS (1× MEM, 5% horse serum, 0.22% NaHCO_3_, 0.6% glucose, 2 mM glutamine, 1× essential and non-essential amino acids, pH 7.3). After mechanical dissociation with fire-polished Pasteur glass pipettes, we counted and adjusted the neuron density.

For immunocytochemistry, neurons were plated on PLL-coated 15-mm round glass coverslips immersed in MEM-HS (VWR, Marienfeld 630-1597) at a density of around 70 cells per mm^2^. After 2 h of incubation in the culture chamber, MEM-HS was replaced with glia-conditioned N2 medium (1× MEM, 1 mM sodium pyruvate, 1% Neuropan2 supplement (Pan-Biotech), 0.22% NaHCO_3_, 0.6% glucose, 2 mM L-glutamine and 2% B27 supplement).

For live-cell imaging of hippocampal neurons, 5 × 10^5^ cells and 3 µg plasmid DNA encoding the gene of interest fused with a selected fluorescent protein were transferred to a nucleofection cuvette for electroporation (built-in program 0-005) with an Amaxa Nucleofector II (Lonza). After electroporation, 7.5 × 10^4^ cells were plated on PLL-coated glass-bottom 8-well or 4-well chamber slides (Ibidi). After 2 h of incubation, MEM-HS was replaced with astrocyte-conditioned N2 medium. We acquired the images immediately after exchange of the medium or after an additional 16 h of incubation.

Neuron cultures in 3D collagen matrix were conducted in accordance with our previously published protocol^71^. In brief, the matrix solution (3.4 mg ml^–1^ collagen and 1× MEM, 0.3% NaHCO_3_) was prepared on ice to keep the solution in liquid phase. After mixing with a suspension of neurons (matrix solution to neuron suspension ratio of 3:1), a 40-µl drop of matrix–cell mix with a neuron density of 0.75–1.5 × 10^6^ cells per ml was directly applied to the bottom of each well of 8-well glass bottom chamber slides (Ibidi). After incubation in a neuron culture chamber for 20 min, conditioned N2 medium was added to fully cover the solidified matrix droplet.

For experiments manipulating the neuronal cytoskeleton, chemical stock solutions were diluted in glia-conditioned N2 medium to achieve the indicated working concentrations. DMSO of the same volume of the added stock solution was used as controls. Treatment of cultured neurons was initiated after exchange with MEM-HS after 2 h of seeding. On the basis of published works^56,59,65,80^, the final concentration of each chemical was as follows: 20 µM blebbistatin, 40 µM *para*-blebbistatin, 100–200 µM CK-666, 5 nM taxol and 75 nM nocodazole. For the washout experiments, we replaced the medium containing the indicated chemicals with fresh conditioned N2 medium at 24 h after plating.

### Immunocytochemistry

Chemical fixation of cultured neurons on coverslips was performed with PHEM fixative (4% paraformaldehyde, 4% sucrose, 0.25% glutaraldehyde, 0.1% Triton X-100, 60 mM PIPES, 25 mM HEPES, 10 mM EGTA and 2 mM MgCl_2_, pH 6.9) for 15–20 min. Alternatively, a modified PHEM fixative (4% paraformaldehyde, 4% sucrose, 60 mM PIPES, 25 mM HEPES, 10 mM EGTA and 2 mM MgCl_2_, pH 7.4) was used. After washing out the fixative with PBS solution, the residual fixatives were quenched with 0.1 M glycine in PBS or 50 mM ammonium chloride for 10–15 min, followed by incubation with blocking solution (2% FCS, 2% BSA and 0.2% fish gelatin in PBS) at room temperature for 1 h. When the modified PHEM fixative was used, permeabilization with 0.1% Triton X-100 in PBS for 3 min was done before blocking.

Fixed neurons were incubated with the indicated primary antibodies diluted in 10% blocking solution at room temperature for 1 h or at 4 °C overnight. After washing out unbound antibodies with PBS, dye-conjugated secondary antibodies or chemical probes were applied at room temperature for 1 h. Coverslips were mounted onto glass slides using Fluoromount solution (F4680, Sigma) for confocal microscopy or using ProLong Gold Antifade solution (P36930, ThermoFisher) for super-resolution imaging.

The following primary antibodies were used: anti-tau1 (1:1,000; MAB3420, Millipore), anti-tubulin-β3 (1:2500; T2200, Sigma), anti-myosin IIb (1:1,000, 8824, Cell Signalling Technology), anti-ARP3 (1:500, A5979, Sigma-Aldrich; 1:200, 0727-2, Millipore) and anti-GFP (1:1,000; Abcam ab13970). Validation of the primary antibodies is shown in Supplementary Table 1.

The following secondary antibodies were used: Alexa Fluor 405 anti-rabbit (1:1,000; A31556, Invitrogen), Alexa Fluor 555 anti-mouse (1:1,000; A21422, Invitrogen), Alexa Fluor 488 anti-rabbit (1:1,000; A11034, Invitrogen), Alexa Fluor 488 anti-chicken (1:1,000; A11039, Invitrogen), Alexa Fluor 594 anti-rabbit (1:500; A-21207, Invitrogen), Alexa Fluor 594 anti-mouse (1:500; A-11032, Invitrogen), Phalloidin-Atto 647N (1:2500; 65906, Sigma) and Phalloidin-Alexa Fluor 647Plus (1:400; A30107, Thermo).

For morphological phenotype characterization, images were acquired on a Zeiss Axiovert 135TV inverted microscope equipped with a CCD camera (COHU Mod 4912) controlled by Zeiss ZEN Blue or on an AxioObserver equipped with an LED Colibri illumination system and an Axiocam 512 mono camera. Alternatively, a DeltaVision RT and a DeltaVision Elite equipped with a Photometrics CoolSnap HQ camera (Roper Scientific) were used. For neuronal cytoskeleton characterizations, super-resolution Airyscan images were acquired with the SR mode on a Zeiss LSM 980 confocal microscope equipped with an Airyscan 2 detector and a Plan-Apochromat ×63/1.4 oil objective. For the imaging of neurons grown in 3D collagen matrix, *z*-stacked images were acquired with a confocal microscope (Zeiss LSM 700). Deconvolution and Airyscan post-processing were performed with Zen Blue (Zeiss).

### Western blots

We used cortical neurons for optimal extraction of biomolecules. Cortical neurons were plated at high density (350 cells per mm^2^) onto PLL-coated plastic 6-cm or 6-well 3-cm dishes (Nunclon Delta surface, ThermoFisher). Cells were resuspended and lysed in ice-cold RIPA buffer (10 mM Tris-Cl pH 7.5, 150 mM NaCl, 0.5 mM EDTA, 0.1% SDS, 1% Triton X-100 and 1% deoxycholate) supplemented with phosphatase inhibitor (PhosSTOP, Roche) and protease inhibitor (cOmplete Mini, Roche) cocktail tablets. After removal of the insoluble fraction via centrifugation, the soluble fraction was heat-denatured in SDS–Laemmli sample buffer. After resolving the protein mixture with 12% polyacrylamide–SDS gels, the resolved proteins were transferred onto methanol-activated PVDF membranes (Immobilon-P^SQ^, Millipore).

Membranes were then incubated in blocking solution (5% skim milk in PBS) and then incubated with primary antibodies (listed below) diluted in blocking solution at 4 °C overnight. After washing out unbound primary antibodies with washing solution (TBS, 0.1% Tween-20), the membranes were incubated at room temperature with secondary antibodies (listed below) diluted in blocking solution for 1 h. After removal of unbound secondary antibodies with washing solution, the membranes were treated with horse radish peroxidase (HRP) substrate (SuperSignal West Femto, ThermoFisher), and the films (CL-Xposure, ThermoFisher) were developed using an AGFA Curix 60 developer. Uncropped scans of the films are shown in Supplementary Fig. 1.

GAPDH was used as a loading control. When multiple rounds of blotting of proteins on a single membrane was required, the bound antibodies were stripped from the membranes with stripping solution (0.2 M glycine pH 2.5 and 0.05% Tween-20) at 85 °C for 30 min. Alternatively, the membrane was divided on the basis of the protein molecular weight and incubated separately with the indicated primary and secondary antibodies.

The following primary antibodies were used: anti-ARP3 (1:1,500; 07-272, Millipore), anti-ARP2 (1:1,500; 5614S, Cell Signaling), anti-ARPC1a (1:500; HPA004334, Sigma), anti-ARPC2 (1:1,000; 07-227, Millipore), anti-ARPC3 (612234, BD Biosciences), anti-ARPC4 (1:400; sc-68394, Santa Cruz), anti-ARPC5 (1:400; sc-166760, Santa Cruz), anti-phospho-MRLC (1:1,000, ab2480, Abcam; 1:1,000, 3675, Cell Signaling), anti-MRLC (1:800, PA5-17624, ThermoFisher; 1:1,000, 3672, Cell Signaling) and GAPDH (1:10,000; ACR001P, Acris). Validation of the primary antibodies is shown in Supplementary Table 1.

The following secondary antibodies were used: StrepMAB-Classic-HRP (1:20,000; 2-1509-001, IBA Lifescience), anti-mouse-HRP (1:20,000; 31432, ThermoFisher) and anti-rabbit-HRP (1:20,000; 31458, ThermoFisher).

### IUE experiments

We followed a previously described IUE protocol^81^. Timed-pregnancy mice fostering embryos (E14.5 for sparse labelling and E12.5 for neuron-specific KO) were anaesthetized under a constant flow of isoflurane (Abbot) and the uterus was carefully exposed from the abdominal cavity. Throughout surgery, warm saline was used to prevent dehydration. The lateral ventricles of embryos were each filled with 1–3 µl of a 10:1 mix of EndoFree DNA of interest and Fast Green dye (Sigma) using micropipettes pulled in a pipette-puller device (Zeitz) and a Picospritzer III microinjection device (Intracel). The DNA samples used for electroporation included pTα-Cre, pTα-Dre, pTα-Vika, pCAG-rox-STOP-rox-Lifeact-mScarlet, pCAG-vox-STOP-vox-ZsGreen and pCAG-rox-STOP-rox-LYN(PM)-mNeonGreen. Tweezertrodes electrodes controlled by an ECM 830 electroporator (BTX Harvard Apparatus) were used to deliver 5 pulses at 35 mV with 50-ms duration and 600-ms intervals. Following electroporation of embryos, the uterus was returned into the abdomen, which was carefully stitched. The mother was euthanized when embryos were at E15.5 (Fig. 1c,h) or E17.5 (Fig. 4g). The genotypes of the embryos were determined by PCR.

### Cryosectioning and imaging

Brains from E17.5 mice were fixed with 37 °C pre-warmed PHEM fixative for 2 h. After additional incubation at 4 °C for 16 h, the heads were incubated in 30% sucrose in PBS for 48 h. The PBS-washed brains were embedded in cryoembedding medium, frozen and sliced into 20–50-μm-thick sections.

Cryosections of embryonic brains expressing ZsGreen or Lifeact–mScarlet were aligned on the glass slides and mounted with Fluoromount solution (F4680, Sigma). Tiles of *z*-stacked images covering the fluorescence-positive cortical regions were acquired with SR mode on a Zeiss LSM 980 confocal microscope. Airyscan post-processing and maximum intensity projection were performed using Zen Blue (Zeiss). Tile stitching was performed with Imaris Stitcher (v.9.0.4).

Cryosections of embryonic brains without IUE were stained with mouse anti-tau1 (PC1C6, 1:400; Chemicon) and AlexaFluor 488-conjugated goat anti-mouse for axons and with DAPI (1:5,000, Invitrogen) for nuclei. After staining, brain sections on glass slides were mounted with Fluoromount solution (F4680, Sigma). Tiles of confocal stacks were acquired using a Zeiss LSM700 confocal microscope. The image stacks were projected with maximum intensity and the tiles were reconstructed using ZEN Blue (Zeiss).

### Organotypic culture of brain slices for live-cell imaging

Organotypic cultures of brain slices were conducted as previously described in detail^81^. In brief, heads of embryos with brains expressing LYN(PM)-mNeonGreen or Lifeact–mNeonGreen were collected in Hank’s balanced salt solution (HBSS, Sigma) supplemented with 0.5% glucose (HBSS-Glucose). Brains dissected from skulls were embedded in 3% low-melting point agarose (Biozym) and cut into 150-µm-thick coronal sections flanking the electroporated areas with a VT1200S vibratome (Leica). Brains were cut and kept in cold HBSS-Glucose. Brain slices were laid on 30 mm polytetrafluoroethylene membranes (Millipore) in 35 mm Transwell plates (Fluorodish, WPI). Slice medium (Neurobasal 1×, FCS 5%, B27 supplement 1:50, Glutamax 1:400, penicillin–streptomycin 1:200, horse serum 5% and Neuropan-2 supplement 1:100 pH 7.3) was injected underneath the membrane to support growth. After incubating at 35 °C and 5% CO_2_ for 4–8 h, slices were imaged with a Zeiss LSM880 confocal microscope equipped with a 488-nm laser line and a ×32 objective (C-Achroplan ×32/0.85 W Corr M27 VIS-IR) as *z*-stack tiles with a frame rate 0.5 frames per min for 2 h.

### Time-lapse live-cell microscopy for profiling of neurite growth and protein intensity fluctuations

Time-lapse live-cell images of 2D cultures were acquired with widefield epi-fluorescence microscopes (DeltaVision RT and DeltaVision Elite, Applied Precision). Both microscopes were equipped with Photometrics CoolSNAP HQ cameras (Roper Scientific) and incubation chambers maintaining conditions for primary neuron cultures. Image acquisition and deconvolution were performed with SoftWoRx (v.3.5 or 4.0, Applied Precision).

To profile native neurite growth of early-stage neurons, differential imaging contrast images were acquired with a frame rate of 1 frame per min for at least 8 h. To profile filamentous actin fluctuations, we used the live-cell actin probe Lifeact, which has been applied in profiling the dynamics of the leading edge of migrating cells^82,83^. As high levels of Lifeact expression affect native actin dynamics and organization and potentially create artefacts in our analyses^84,85^, we excluded neurons with high levels of Lifeact from image acquisition.

Neurons expressing Lifeact–mScarlet were acquired with a frame rate 0.25 frames per min for at least 12 h. To profile fluctuations in ARP2/3 and myosin II levels, dual-channel images (Lifeact–mScarlet with ARP3–SNAP-SiR or MRLC–SNAP-SiR) were acquired with a frame rate of 0.2 frames per min for at least 12 h. To profile ARP2/3 and myosin II fluctuations simultaneously, triple-channel images (Lifeact–mScarlet, ARP3–SNAP-SiR and MRLC–mNeonGreen) were acquired with a frame rate of 0.1 frames per min for at least 12 h. To profile subcellular actin (Lifeact–GFP) and microtubule dynamics (EB3–mCherry), the images were acquired with a frame rate of 1 frame per s for 3 or 5 min.

Time-lapse live-cell images of neurons expressing Lifeact–mScarlet and grown in 3D collagen matrix were acquired with a Zeiss LSM880 confocal microscope equipped with a ×20 objective (Plan-Apochromat ×20/0.8 NA M27) as *z* stacks with a frame rate of 1 frame per 15 min for at least 12 h. Deconvolution post-processing was performed using Zeiss Zen Blue.

### Machine-learning-assisted segmentation of brightfield images of neurons

To facilitate extraction of neurite growth profiles from the low-contrast brightfield time-lapse images of neurons, we applied convolutional neural networks to segment the images. The segmentation process defined and distinguished the background, the soma and the neurites from each other.

We performed supervised pixel classification on the basis of machine learning on the time-lapse image stacks. We used the open-source software YAPiC (https://yapic.github.io/yapic/) to train a 2D U-Net (‘unet_2d’ network of YAPiC) with three classes (background, soma and neurites). Training data were collected by manually labelling a subset of time-lapse images with Ilastik software (https://ilastik.org)^86^. A total of 18 images of 10 WT neurons and 19 images of 11 *Actr3* KO neurons were labelled. In each image, parts of the soma, parts of the neurites and parts of the background were labelled. To obtain precise segmentation of neurites, in each image, several small sections of neurites at different positions were labelled with the directly adjacent background at pixel-level precision. Larger regions of the background were also roughly labelled, and the soma was labelled more roughly than neurites. More detail is described in documentation available at GitHub (https://yapic.github.io/yapic/example_neurite.html).

The network was trained on an Ubuntu 16.04 workstation equipped with Nvidia TITAN V GPU (12 GB RAM). The labelled data were split into 80% training data and 20% validation data (YAPiC default settings) and trained for 5,000 iterations. Computation time for model training was 5 days. The model with the lowest loss of the validation dataset was applied to all image stacks.

### Optogenetic control of ARP2/3 activation with PA-RAC1

We used a previously described PA-RAC1 construct^37^ for optogenetic experiments with an Andor spinning disk confocal microscope or a Zeiss LSM980 confocal microscope.

The Andor spinning disk microscope was built on an inverted Nikon Eclipse Ti microscope and equipped with a Nikon Perfect Focus system, a Yokogawa CSU-X1 Spinning Disk Unit, a REVOLUTION 500 series AOTF Laser module, iXON EMCCD and Neo Monochrome sCMOS dual cameras and a FRAPPA photobleaching module. The operation and parameter setting were performed with Andor iQ3. We used Nikon Plan Apo ×40 oil objective N.A. 1.4 for light activation and image acquisition.

Neurons expressing mVenus–PA-RAC1 were selected. The photoactivation sites were manually defined as single or multiple spots of 1–4 pixels. PA-RAC1 at the selected sites was sequentially photoactivated with a 450-nm laser controlled by the FRAPPA module. The dwell time of the laser on each pixel was 20 µs and the repetition number was 500. Time-lapse single-plane images of Lifeact–mScarlet were acquired every 10 s before, during and after the photoactivation step. A total of 20 frames were acquired for each session.

For the optogenetic experiments performed with a Zeiss LSM980 confocal microscope, a ×40 objective (Zeiss LD LCI Plan-Apochromat ×40/1.2 Imm Corr DIC M27) was used for photoactivation and Airyscan image acquisition. The photoactivation sites were manually defined as a single region of 4–12 pixels in Zeiss ZEN Black. PA-RAC1 at the selected sites was photoactivated with a 445-nm laser. The spot-bleaching duration was 500 and iteration was 400. Time-lapse single-plane dual-channel Airyscan images (Lifeact–mScarlet and MRLC–SNAP-SiR) were acquired every 20 s before, during and after the photoactivation step. A total of 20 or 30 frames were acquired for each session.

Identical settings for an Andor spinning disk or LSM980 microscope were followed for the control experiment using a photoinsensitive variant of PA-RAC1 (PA(C450M)-RAC1) and for the ARP2/3 suppression experiment using the dominant-negative variant (PA-RAC1(T17N)).

For quantification, the last frame before photoactivation (pre), the last frame of photoactivation (act) and the last frame of deactivation (post) were analysed. To reduce variations caused by heterogeneity of exogenous protein expression and differences in neurite length among neurons, we applied a normalization process. We calculated the direction and the amplitude of changes (intensity and neurite length) relative to the mean by deducting the mean value from the measured values.

### Negative-staining electron microscopy

Before cell seeding, Formvar-film-coated 200-mesh Au grids (Gilder Grids) were glow-discharged and coated in 1 mg ml^–1^ poly-lysine (Sigma-Aldrich) at room temperature overnight. Subsequently, grids were washed 3 times with PBS, placed in grid holders^87^, transferred into 96-well plates and incubated with conditioned N2 medium at 36.5 °C for 1 h. For seeding of cells onto EM grids, cells were freshly thawed and diluted to achieve a seeding density of approximately two cells per grid square. At 2 h after seeding at 36.5 °C, a medium exchange with fresh conditional N2 medium was performed. Finally, cells were incubated at 36.5 °C for 20 h.

Cells were prepared for negative-staining transmission electron microscopy as previously described^88^, but with minor modifications. In brief, neurons were extracted and mildly fixed in incubating grids in 50 μl droplets of cytoskeleton buffer (10 mM MES, 150 mM NaCl, 5 mM EGTA, 5 mM glucose and 5 mM MgCl_2_, adjusted to pH 6.2) containing 0.75% Triton X-100 (Sigma-Aldrich), 0.25% glutaraldehyde (Electron Microscopy Sciences) and 0.1 μg ml^–1^ phalloidin (Sigma-Aldrich) for 1 min. For post-fixation, grids were incubated in 50 μl droplets of cytoskeleton buffer containing 2% glutaraldehyde and 1 μg ml^–1^ phalloidin for 15 min. Negative staining was performed by dropwise application and immediate blotting of 50 μl total volume of 4% negative-staining solution (10 nm BSA-conjugated gold colloid diluted 1:8 in 4% sodium silicotungstate (Agar Scientific), adjusted to pH 7.0).

Dual tilt axis tomography of negatively stained cytoskeletons was performed on a FEI Tecnai G2 20 operated at 200 kV using SerialEM software^89^. Data were recorded on a FEI Eagle 4k camera at a magnification of ×29,000, resulting in a pixel size of 7.767 Å. Each unidirectional tilt series ranged from −60 to +60° in 1° increments. The defocus was set to −3 μm.

Tilt series alignment and tomogram reconstruction via weighted backprojection was performed in IMOD^90^ by combining the data from two related tilt series obtained around orthogonal axes.

### Machine-learning-assisted segmentation and automatic actin filament tracking

To facilitate automatic filament tracking, we applied convolutional neural networks to segment the tomograms and filter out the background and artefactual membrane fragments. We performed supervised pixel classification based on deep learning on all collected EM raw data stacks. We used the open-source software YAPiC (https://yapic.github.io/yapic/) to train a multislice U-Net (‘unet_multi_z’ network of YAPiC) with two classes (background region and actin filament region). Training data were collected by manually labelling a subset of image stacks (3 WT and 3 *Actr3* KO stacks, each containing 50–100 slices) with Ilastik software (https://ilastik.org)^86^. Approximately 20% of all pixels in the images were labelled.

The network was trained on a Ubuntu 16.04 workstation equipped with Nvidia TITAN V GPU (12 GB RAM). The labelled data were split into 80% training data and 20% validation data (YAPiC default settings) and trained for 5,000 iterations. Computation time for model training was 5 days. The model with the lowest loss of the validation dataset was applied to all image stacks. This process is described in greater detail in the documentation available at GitHub (https://yapic.github.io/yapic/example_actin_em.html).

The stacks of the actin network model were converted to a mask and applied to the original reconstructed tomograms to remove background and artefacts, leaving the pixels of actin filaments only (Extended Data Fig. 9a). We then performed automatic actin filament tracking with the modified Matlab scripts as previously described^88,91^.

### Actin filament polarity determination

Analysis of actin filament polarity was done as previously described^92^. In brief, traces of the filaments were interpolated by a 3D spline curve, and subtomograms, including the actin filaments, were extracted along the spline curves. The actin filament in the extracted subtomogram was traced again automatically through correlation with a 3D cylinder. The filament was unbent according to the trace. The unbent filament was 2D-projected onto a plane including the filament axis with the smallest tilt angle against the grid plane. The projected images were analysed using single-particle analysis procedures for filamentous complexes and the filament polarity was determined. The analysis code has been deposited into Zenodo (https://doi.org/10.5281/zenodo.20081075)^93^.

### CK-666-induced neurite retraction

E17.5 mouse hippocampal WT neurons were isolated. After introducing plasmids encoding the indicated reporters (Lifeact–mScarlet, MRLC–SNAP and EB3–mNeonGreen) via electroporation, neurons were cultured on PLL-coated 8-well chamber slides. Time-lapse images were acquired with widefield epi-fluorescence microscopes (DeltaVision RT or DeltaVision Elite, Applied Precision). To have neurons with developed neurites, we started image acquisition 48 h or 72 h after plating for the pre-CK-666 condition. After this session, we pre-mixed the CK-666 stock solution with half of the volume of conditioned N2 medium in the well and then added this mixture back to the original well to achieve a final CK-666 concentration of 150 µM. In this way, we kept the neuron-secreted autocrine growth factors consistent. After 10–15 min of temperature re-equilibration and stage re-focusing, we started another session of image acquisition for the post-CK-666 condition. The acquisition duration was 16–24 h. After this session, cells were washed three times with pre-warmed fresh conditioned N2 medium. After temperature re-equilibrium and stage re-focusing, we started another session of image acquisition for the CK-666-washout condition. Images were deconvoluted with SoftWoRx (v.3.5 or 4.0, Applied Precision).

For investigation of the impact of CK-666 on the lengths of axons and minor neurites of DIV-1 and DIV-3 neurons, DIV-1 (32–36 h after plating) and DIV-3 (80–84 h after plating) neurons were treated with CK-666 (200 µM) dissolved in DMSO. After 12 h of treatment, the treated neurons were fixed with modified PHEM fixative for 20 min and immunostained as described in the above. The neurite tracks and lengths were defined using the Segmented Line of Fiji.

### Local perfusion of CK-666 and *para*-aminoblebbistatin

Hippocampal WT neurons were isolated, and plasmids encoding Lifeact–mScarlet were introduced via electroporation. Neurons were cultured on PLL-coated MatTek 35-mm glass-bottom dishes. Before the experiments, cells were washed twice with pre-warmed HBSS solution and finally with pre-warmed and filtered conditioned N2 medium to avoid blockage of the suction pipette with culture debris. To visualize the application fluid during imaging, FastGreen For Coloring Food (FCF) was diluted in HBSS solution. Then we added the filtered FastGreen FCF HBSS mix to freshly filtered conditioned N2 medium (1:4). Fast Green FCF was imaged during the experiment with far-red excitation. To prepare the application fluid, we mixed the CK-666 and *para*-aminoblebbistatin stock solution (diluted in DMSO) with the HBSS FastGreen FCF conditioned N2 mix to achieve a final CK-666 concentration of 150 µM and *para*-aminoblebbistatin of 40 µM. DMSO of the same volume of the added stock solution was used as the control. The application capillary was filled with 10 µl of the application fluid.

For local perfusion, we modified a previously published setup^94^. We used a micromanipulator-controlled application and suction system attached to an Andor spinning disk confocal microscope, configured and controlled as described for the optogenetic control experiments. We used a Nikon Plan Apo ×10/0.45 for positioning the application and suction pipettes in the vicinity of the target neurons and a Nikon Plan Fluor ×40/1.30 for fine adjustment of the pipettes and subsequent image acquisition. To establish local perfusion, we applied positive pressure (3–5 hPa) to the infusion capillary and negative pressure (0.5–1.5 hPa) to the aspiration capillary with two motor-controlled syringe pumps. Once the position and pressure were adjusted to establish a defined local perfusion at the focal plane, the infusion pressure was reduced to stop the outflow. The aspiration pressure was kept constant throughout the experiment.

After placing the target soma or growth cone at the designated local perfusion site, we started image acquisition and local perfusion by initiating the previously established application pressure. After 90 min of local application, the application pressure was released, and the application and suction pipettes were removed. The neuron was imaged for at least 1 h after application. Image analysis was performed using Fiji (v.1.48o).

### Quantification of EB3 intensities at growth cones and neurite tips

We manually defined the area of growth cones and neurite tips using the Lifeact channel, which visualizes actin protrusions. We used EB3 intensity to approximate the level of polymerizing microtubules. To reduce interference of photobleaching on measurements, we only analysed the temporal maximum intensity projection of the first 24 frames over 2 min before and after CK-666 treatment.

### Neurite tracking, growth profiling and local protein intensity profiling

The procedures below were performed manually with the indicated Fiji plugins or batch-processed with customized ImageJ and Jython macro scripts in Fiji using ImageJ API. The custom ImageJ macros used for generating kymographs, extracting neurite tip positions and protein intensities have been deposited into GitHub (https://github.com/darkbreaker0/IJ_NeuriteGrowthScript).

We used segmented brightfield images of early-stage neurons to define the neurite tracks and to profile native neurite growth. Alternatively, to quantitatively characterize the relationship between neurite growth and local protein fluorescence intensity fluctuations, the actin channel (Lifeact–mScarlet or Lifeact–mNeonGreen) of the images was subjected to neurite track determination and growth profiling. For neurite growth profiling in cortical slice cultures, we used the LYN–mNeonGreen channel. The neurite growth profiles derived from the segmented brightfield and actin fluorescence intensity gave the same neurite growth dynamic.

To define neurite tracks, a temporal projection from the time-series image stacks was generated, which showed the trajectories of neurite growth. Neurite paths were then manually tracked with ‘Segmented Line’. To include the background intensity in the subsequently generated kymograph, the length of the neurite track was at least 10 μm longer than the corresponding neurite. To reduce complexity, neurites were only analysed if they directly originated from the soma such that secondary and tertiary neurite branches were excluded. Following the neurite tracks, the kymographs were generated with the ‘Multi-Kymograph’, with a line width of 3.2 μm used to cover the width of the neurite shaft. This then calculated average intensity across the line width.

To extract neurite growth profiles from the kymographs, a thresholding filter ‘Huang’ was first applied and then the upper threshold value was manually adjusted to define the end positions of the neurite tip. The tip point was defined as the position of the pixel with an intensity above the upper threshold and the longest *x* axis value. In cases when the bright background speckles interfered with identification of the tip position, the speckles were manually removed from the kymographs beforehand. The end positions of the neurite tip at each time point along the kymograph were defined as the neurite lengths.

To approximate the indicated protein level at the neurite tip, the protein fluorescence intensity was integrated in the 6.2-μm window from the neurite tip at each time point along the kymograph. The protein level at the neurite shaft was defined as the integrated intensity of the whole neurite minus the integrated intensity of the neurite tip. As the integrated intensity at the neurite shaft is proportional to the neurite shaft length (neurite length minus 6.2 μm), the intensity profiles were normalized with the length profiles, which resulted in profiles of actin density at the neurite shafts. To quantify the intensity fluctuations of the indicated proteins at the soma, the soma area was manually defined using ‘Polygon Selection’ and intensity was profiled using ‘Measure stack’.

### Cross-correlation of neurite growth dynamics and local protein intensity fluctuations

The extracted neurite growth profiles and the local protein fluorescence intensity profiles were imported and analysed in the integrated development environment RStudio as multiple time-series data registered to the associated neurons (R packages stats::ts and tsibble::as_tsibble). To reduce noise as high-frequency background fluctuations, moving average smoothening (R package forecast::ma) was applied to the multiple time-series data with a window size of 3 for the data with low temporal resolution (profiles derived from cortical slice cultures and triple-channel images) or with a window size of 5 for the data with high temporal resolution (profiles derived from segmented brightfield images and single-channel and dual-channel images).

For the neurite extension–retraction cross-correlation analysis, differential growth profiles of the neurites were generated (R base diff) from the smoothened time-series data (Extended Data Fig. 1d). This differencing step calculated the first derivative (velocity) of the smoothened time-series data over time. Next, at any given time point, from the extending neurites, the positive changes were summed and integrated extension activity profiles were generated (Extended Data Fig. 1d, yellow blocks). Similarly, from the retracting neurites, integrated retraction activity profiles were generated. Pearson’s correlation coefficient between the extension and the retraction was computed as a function of the time lag using the R package stats::ccf. The correlation functions from different cells were pooled, and the averaged cross-correlation function and the standard deviation were calculated (R package rstatix::get_summary_stats).

For the cross-correlation analysis between neurite growth and local protein fluorescence intensity fluctuations, two strategies were applied. The first strategy excluded soma protein intensity profiles and considered one neurite as one sample. It computed the Pearson’s correlation coefficient between the differential growth profile of each neurite and the associated neurite tip or normalized neurite shaft protein intensity differential profiles (R package stats::ccf). The correlation values from all the neurites of different cells were pooled, and the averaged cross-correlation value and the standard deviation were calculated (R package rstatix::get_summary_stats). This strategy is more sensitive in detecting correlations at the individual neurite level.

The second strategy included soma protein intensity profiles and considered one neuron as one sample. The assumption here is that soma protein intensity fluctuations reflect the integration of the overall relationships with every neurite. Consequently, the soma protein intensity fluctuations are less correlated with any one neurite but more with the overall neurite growth and with the overall neurite protein fluorescence intensity fluctuations. To obtain the integrated measurements of all neurites, the measured values of every neurite were summed first (Extended Data Fig. 1d, brown blocks). After moving-average smoothening (R package forecast::ma), the first derivatives of the summed measured values were calculated (R base diff). Pearson’s correlation coefficients of the paired differential profiles from different cells were pooled for the calculation of the averaged cross-correlation function and the standard deviation (R package rstatix::get_summary_stats).

### Quantification of ARP3 and MRLC intensities at extending and the retracting neurites

From the neurite growth profiles, we categorized the neurite growth states as extending, pausing or retracting by setting the growth velocity threshold as ±0.02 µm min^–1^. The following values were then used for classification: extending, ≥0.02 µm min^–1^; retracting, ≤ −0.02 µm min^–1^; and pausing, between −0.02 and 0.02 µm min^–1^.

For each neuron, the ARP3 and MRLC intensities at the neurite tips of extending, pausing and retracting neurites were calculated. To reduce variations caused by the heterogeneity of exogenous protein expression among neurons and batches of experiments, the averaged ARP3 and MRLC intensities in each neuron was normalized as a *Z* score with the averaged value of the three growth states.

### Neurite length and soma area measurement and categorization of early neuronal morphogenesis stages

The stages of early neuronal morphogenesis are defined by the critical events neuritogenesis and axogenesis. We considered a process longer than 16 µm as a neurite, and a neurite longer than 70 µm and with medial-to-distal accumulation of the axon marker tau as an axon. Therefore, neurons without a neurite longer than 16 µm were categorized as stage 1, neurons with a neurite longer than 16 µm were categorized as stage 2, and neurons with a tau-positive neurite longer than 70 µm as stage 3. Neurons exhibiting multiple axons were also considered stage 3 neurons in spite of aberrant neuronal polarization.

We measured neurite lengths using the Fiji plugin Simple Neurite Tracer^95^ or ‘Segmented Line’. The neurite tracks from the soma to the neurite tips were defined by the microtubules visualized with anti-β3-tubulin. The length of neurite precursors of stage 1 neurons was excluded from neurite length quantifications. For the neurite lengths of stage 2 and stage 3 neurons, we defined the neurites excluding the longest neurite as the remaining neurites. To quantify the soma area, we manually defined the soma contour with ‘Polygon Selection’. The above procedures were performed manually with the indicated Fiji plugins or batch-processed in Fiji using ImageJ API.

### Ventricle area and cortical thickness measurement

To quantify the ventricle area of coronal sections of brains from WT and *Actr3* KO E17.5 mice, we manually defined the contour of ventricle regions with ‘Polygon Selection’ and measured the area in Fiji.

To quantify cortical thickness, for each coronal section, we measured the thickness of at least eight regions that were randomly selected. The cortical thickness was measured as the shortest distance between the boundary of the ventricle to the contour of the cortex.

The result files were imported in RStudio (v.1.4.1103) and analysed with a customized R script.

### Patch-like structure identification tracking, counting and size measurement

To track the movement of ARP3 patches, we applied the Fiji plugin MOSAIC Particle Tracker 2D/3D to detect and track the patches^96^. The following parameters for patch tracking were used: radius, 3–5; cutoff, 0.001; Per/Abs, 0.8–1.5; link range, 1; displacement, 10; dynamics, straight lines.

For the quantification of patch number and patch size, we applied the Fiji plugin ComDet (https://github.com/ekatrukha/ComDet/). The following parameters were used: include larger particles; segment larger particles; approximate particle size, 5–10 pixels; intensity threshold, 5–10; ROI shape, ovals.

The result files were imported into RStudio (v.1.4.1103) and analysed with a customized R script.

### Colocalization analysis

To generate the colocalization cross-correlation colour map of selected two channels (ARP3–actin, ARP3–MRLC or MRLC–actin), the images acquired using a DeltaVision microscope were first deconvoluted with SoftWoRx (v.3.5 or 4.0, Applied Precision). To remove the background signals, we applied ‘Subtract Background’ with a rolling radius of 20 pixels. To avoid interference by small, non-specific particles, we applied the ‘FFT bandpass filter’ to filter out objects smaller than 3 pixels. The typical size of ARP3, MRLC and actin patches is larger than 9 pixels for images with a pixel size of 0.065 µm. We analysed the processed images with the Fiji plugin Colocalization Colormap^97^, with the option ‘autothreshold’ checked.

To quantify the colocalization levels of selected two channels (ARP3–actin, ARP3–MRLC or MRLC–actin) at the growth cones and at the soma, the images were processed as described above. The regions of growth cones and the soma were manually defined with ‘Rectangle Selection’. We then applied the Fiji plugin EzColocalization^98^ to quantify the Pearson cross-correlation of the two channels. We selected ‘Costes’ as the autothresholding method.

### Quantification of actin branches and orientation in EM tomograms

Branch junctions were manually selected using the IMOD software package. For determination of filament orientation relative to the leading edge, first the contours representing the individual filaments were reordered so that their last point would represent the barbed end, as determined by the polarity analysis. For calculation of angles, filaments were represented by 2D vectors (only considering *x* and *y* coordinates) pointing from the first point of the respective contour (pointed end) to the last point of the contour (barbed end), and the leading edge was represented by the 2D unit vector of protrusion rotated 90° in a clockwise direction.

Calculations were performed by applying the following equation in a custom Python3.6 script:

$$\mathrm{Angle}\,\mathrm{to}\,\mathrm{leading}\,\mathrm{edge}=\arctan 2({x}_{\mathrm{LE}}\times {y}_{\mathrm{AF}}-{y}_{\mathrm{LE}}\times {x}_{\mathrm{AF}},{x}_{\mathrm{LE}}\times {x}_{\mathrm{AF}}+{y}_{\mathrm{LE}}\times {y}_{\mathrm{AF}})$$

With *x* and *y* representing the *x* and *y* values of the vectors of the leading edge (LE) or the actin filament (AF). The resulting angles in radians were then transformed to degrees as depicted in the figures.

### Statistical information

Data organization and statistical analysis were performed with Excel 2016 (Microsoft), Prism (v.8.0.1 or 9.0.0, GraphPad Software) and with R packages stats, rstatix and ggpubr in R (v.R 4.0.2). The test, the number of data points (*n*) and the number of independent experiments or biological replicates are described in the figure legends. All tests were two-sided (*α* = 0.05) and exact *P* values are reported in the figures or legends. Normality (D’Agostino–Pearson) and homoscedasticity (Bartlett, Brown–Forsythe) were tested before parametric methods.

The following tests were performed in this study: *t*-tests (Figs. 3i and 4d,r and Extended Data Figs. 3b, 4a,f, 8b,c,k,n,q, 9c,d and 11b,c), Mann–Whitney tests (Figs. 1k,l, 2i, 3h,n, 4h,k,o,p and 5l,m and Extended Data Figs. 2f, 5b,i, 6e,g, 7b,d,f, 8e,j,m and 9i), paired *t*-tests (Fig. 2d and Extended Data Figs. 3q and 4b–c), paired Wilcoxon tests (Figs. 2h,j,k, 3l and 4f and Extended Data Figs. 2i–k and 11f,i), paired Wilcoxon tests with Holm correction (Fig. 3c and Extended Data Figs. 3n, 5e, 6d,i and 7h,i), one-way ANOVA with Dunnett’s post hoc (Extended Data Fig. 12a), two-way ANOVA followed by Tukey post hoc (Figs. 4j and 5b,j and Extended Data Figs. 8g,i and 12g,j,m,n), two-tailed chi-square test of independence (Fig. 5f,g and Extended Data Figs. 4e and 11g) or Kruskal–Wallis followed by Dunn’s post hoc with Bonferroni correction (Figs. 3g and 5c,d and Extended Data Figs. 3e,f, 4h, 8f,h,p and 12b,d,f,h,l).

### Reporting summary

Further information on research design is available in the Nature Portfolio Reporting Summary linked to this article.

## Online content

Any methods, additional references, Nature Portfolio reporting summaries, source data, extended data, supplementary information, acknowledgements, peer review information; details of author contributions and competing interests; and statements of data and code availability are available at https://doi.org/10.1038/s41586-026-10755-6.

## Supplementary information

## Source data

## Data Availability

The raw data of the representative images have been deposited into Zenodo (https://doi.org/10.5281/zenodo.20118606)^99^. Owing to the large file size of the raw image data and the processed data used in the analyses that generated the graphs, we archived the image files in the read-only file archive at the DZNE institute. We provide raw data files upon request. The request can be directed to and will be fulfilled by the lead contact F.B. Source data are provided with this paper.

## Code availability

The custom ImageJ macro used for generating kymographs, extracting neurite tip positions and protein intensities is available at GitHub (https://github.com/darkbreaker0/IJ_NeuriteGrowthScript) and Zenodo (https://doi.org/10.5281/zenodo.20118606)^99^. The custom R and Python scripts used in the study are available at GitHub (https://github.com/darkbreaker0/Arp3_neuronal_polarization_2026) and Zenodo (https://doi.org/10.5281/zenodo.20118606)^99^. The code used for polarity determination of the actin filament in tomograms is available at Zenodo (https://doi.org/10.5281/zenodo.20081075)^93^.
