# Supplementary material for: An intrinsic cytoskeletal oscillator establishes neuronal polarity
Source: Nature. 2026 Jul 8;657(8130):213–25. doi: 10.1038/s41586-026-10755-6 (PMC13538037; doi:10.1038/s41586-026-10755-6)
Supplement: Supplementary file 1 [file 41586_2026_10755_MOESM1_ESM.pdf]

nature portfolio

https://doi.org/10.1038/s41586-026-10755-6

Supplementary information

An intrinsic cytoskeletal oscillator establishes neuronal polarity

In the format provided by the authors and unedited

Fig. 4b

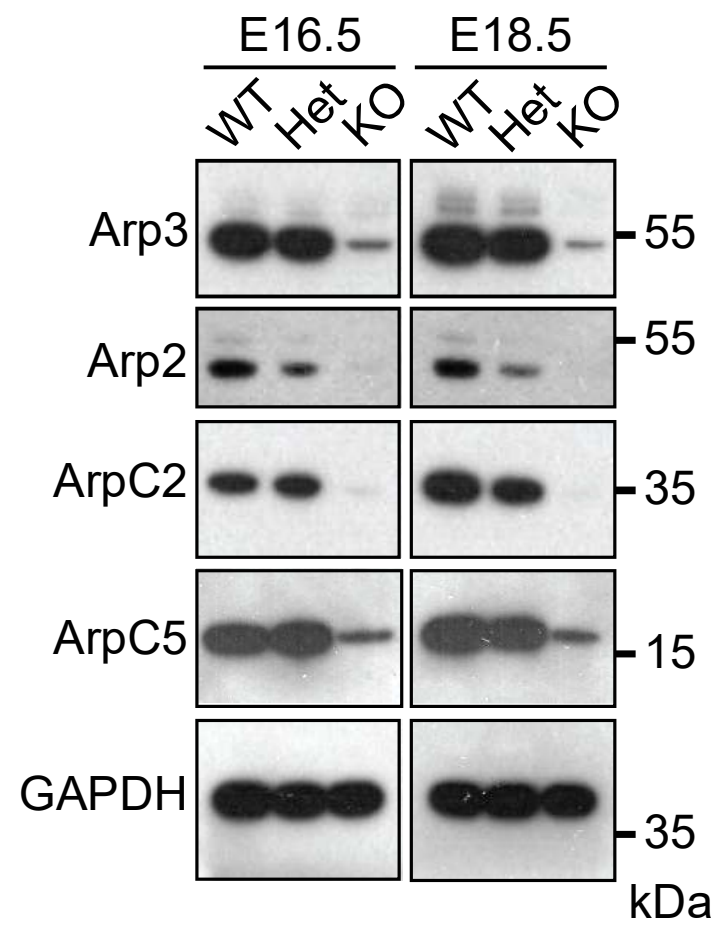

2013_Feb_28

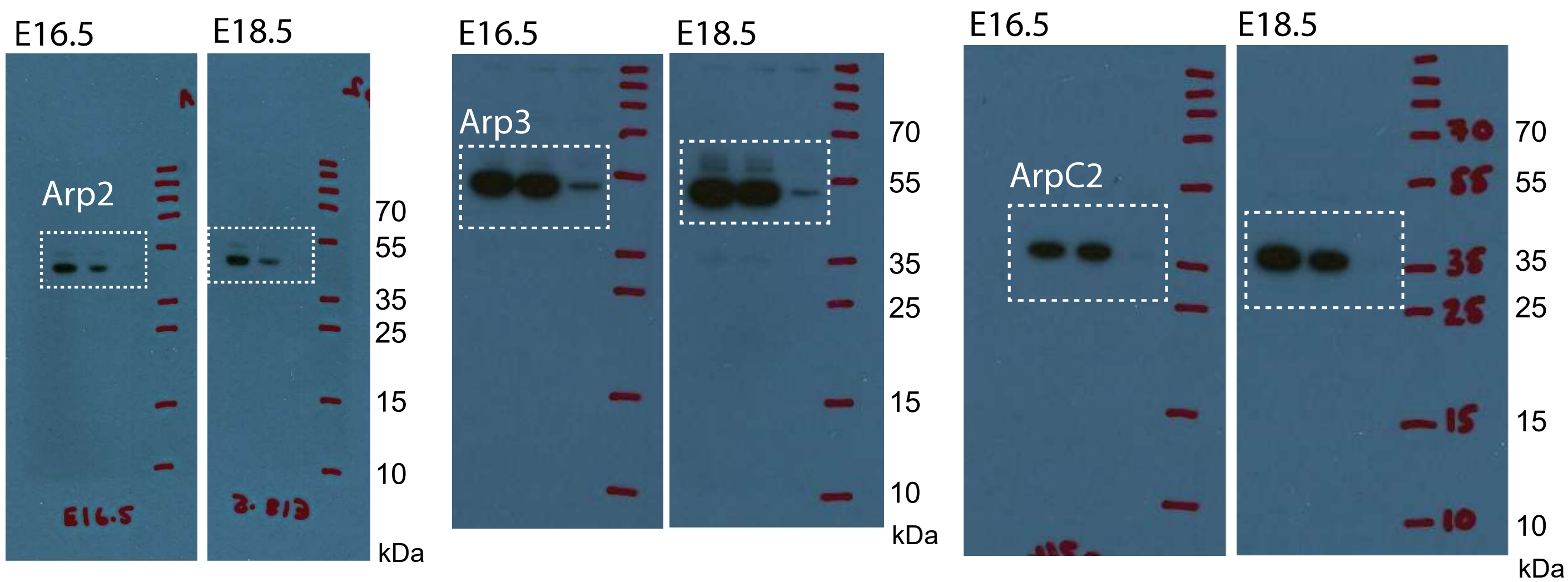

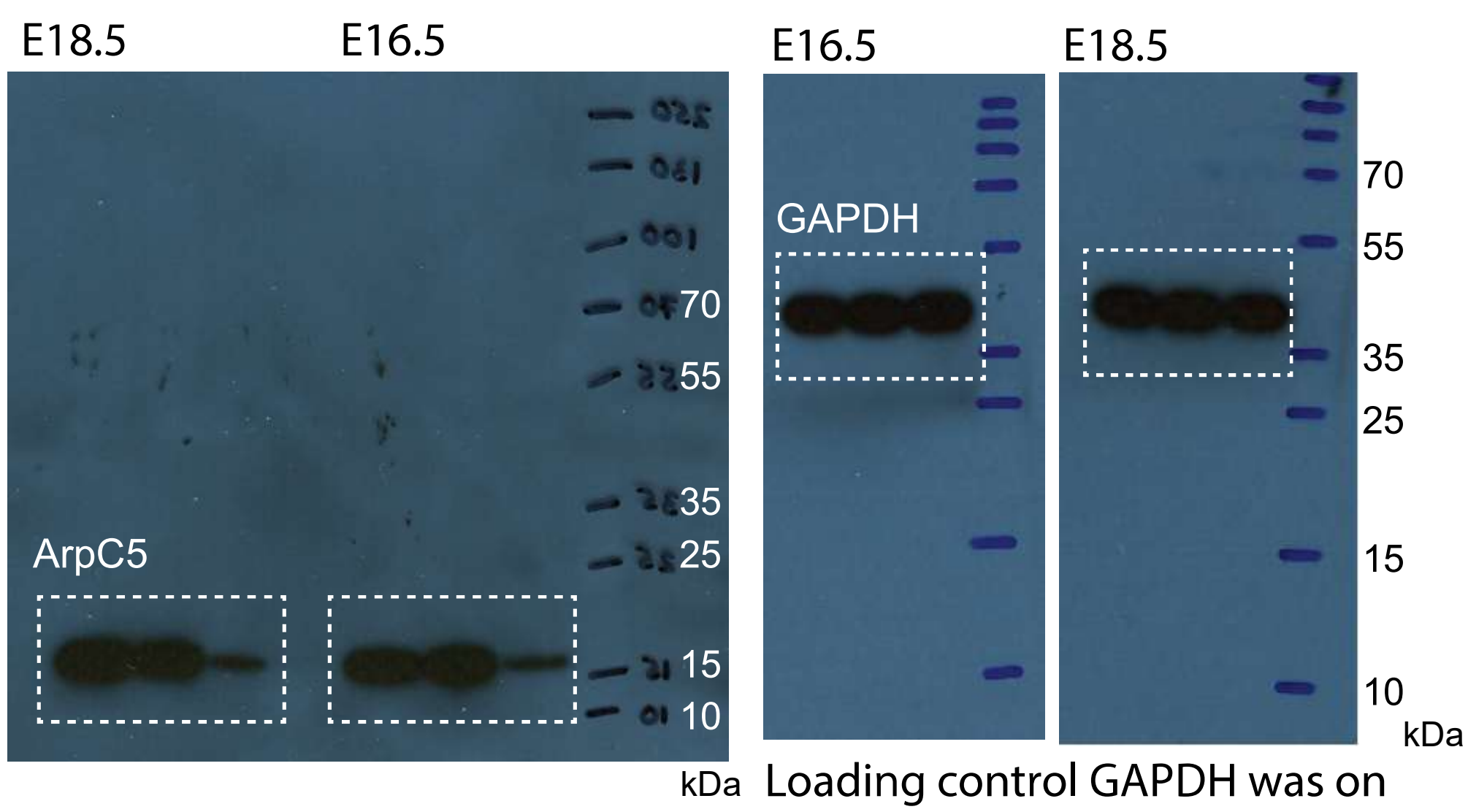

Loading control GAPDH was on the same membrane of Arp3 blot

Fig. 4c

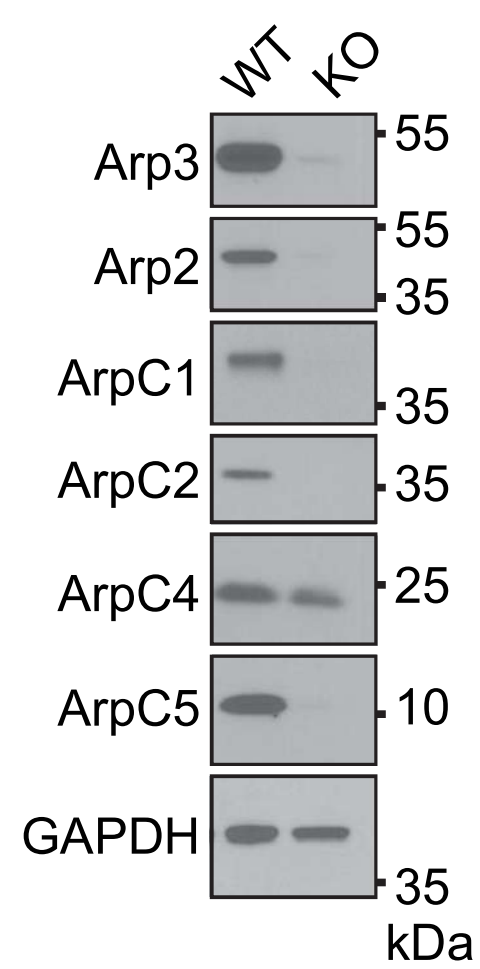

2016_Jan_18-23

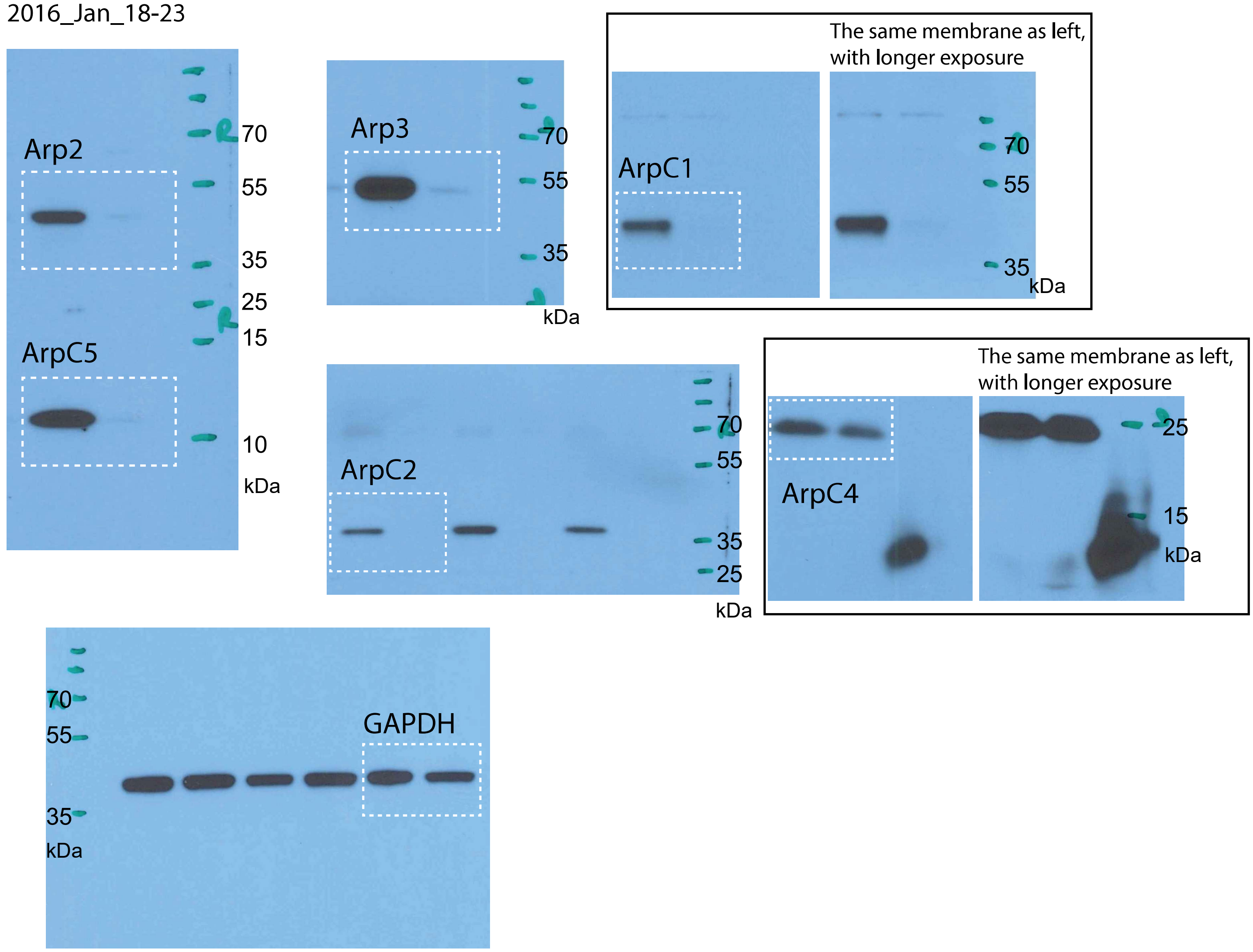

Loading control GAPDH was of the same samples as Arp3 blot but loaded on separate gel

Extended Data Fig. 9h

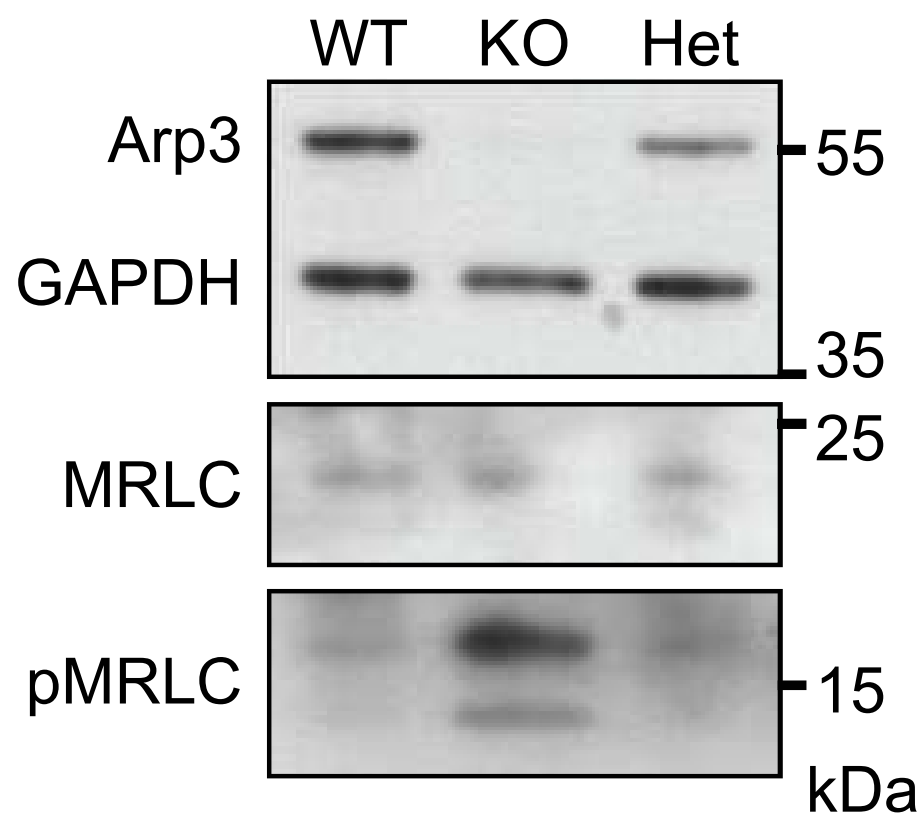

2024_Mar_26

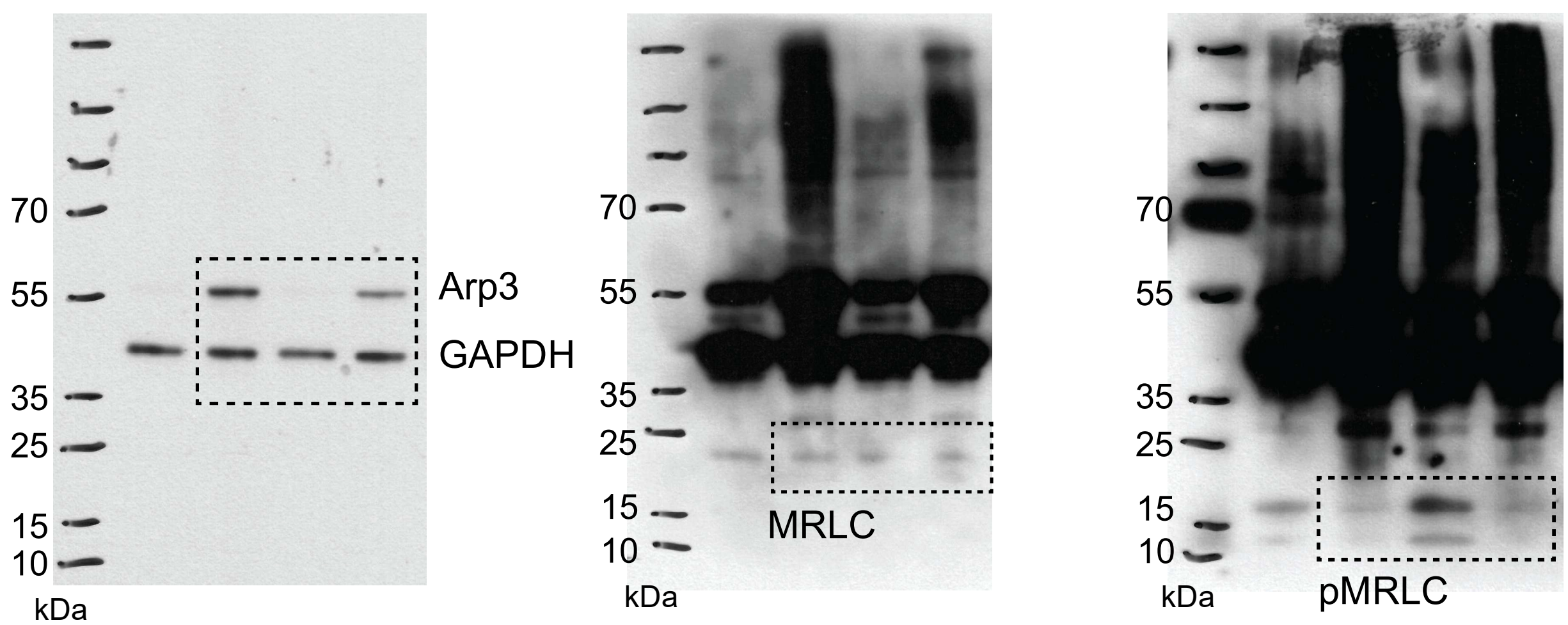

Loading control GAPDH was on
the same membrane of Arp3 blot
