# Supplementary material for: An intrinsic cytoskeletal oscillator establishes neuronal polarity
Source: Nature. 2026 Jul 8;657(8130):213–25. doi: 10.1038/s41586-026-10755-6 (PMC13538037; doi:10.1038/s41586-026-10755-6)
Supplement: Supplementary file 2 [file 41586_2026_10755_MOESM2_ESM.pdf]

nature portfolio

Corresponding author(s): Dr. Frank Bradke

Last updated by author(s): May 11, 2026

Reporting Summary

Nature Portfolio wishes to improve the reproducibility of the work that we publish. This form provides structure for consistency and transparency in reporting. For further information on Nature Portfolio policies, see our Editorial Policies and the Editorial Policy Checklist.

Statistics

For all statistical analyses, confirm that the following items are present in the figure legend, table legend, main text, or Methods section.

n/a	Confirmed	
☐	☒	The exact sample size (*n*) for each experimental group/condition, given as a discrete number and unit of measurement
☐	☒	A statement on whether measurements were taken from distinct samples or whether the same sample was measured repeatedly
☐	☒	The statistical test(s) used AND whether they are one- or two-sided<br>*Only common tests should be described solely by name; describe more complex techniques in the Methods section.*
☐	☒	A description of all covariates tested
☐	☒	A description of any assumptions or corrections, such as tests of normality and adjustment for multiple comparisons
☐	☒	A full description of the statistical parameters including central tendency (e.g. means) or other basic estimates (e.g. regression coefficient) AND variation (e.g. standard deviation) or associated estimates of uncertainty (e.g. confidence intervals)
☐	☒	For null hypothesis testing, the test statistic (e.g. *F*, *t*, *r*) with confidence intervals, effect sizes, degrees of freedom and *P* value noted<br>*Give P values as exact values whenever suitable.*
☒	☐	For Bayesian analysis, information on the choice of priors and Markov chain Monte Carlo settings
☒	☐	For hierarchical and complex designs, identification of the appropriate level for tests and full reporting of outcomes
☐	☒	Estimates of effect sizes (e.g. Cohen's *d*, Pearson's *r*), indicating how they were calculated

Our web collection on statistics for biologists contains articles on many of the points above.

Software and code

Policy information about availability of computer code

Data collection	The image acquisition was performed with SoftWoRx 3.5 or 4.0 (Applied Precision) for DeltaVision, Zeiss Zen Blue 3.3 for Zeiss LSM880 and LSM980, Zeiss Zen Blue 3.11 for Zeiss Axio Observer, Andor iQ3 for Andor confocal spinning disk, Nikon NIS-Elements 6.10 for Nikon CSU-W1 confocal spinning disk. For acquiring EM tomography SerialEM 4.0 was used.
Data analysis	Data were organized using Microsoft Excel (2016), Graphpad Prism (8.0.1 and 9.0.0) and RStudio (Version 1.4.1103). Analysis were performed in Fiji (Version 1.48o), MATLAB (Version R2021b), Imaris (Version 9.2.0) and R-Studio (Version 1.4.1103) as described in Methods.<br><br>The neurite tracks were defined with Fiji plugin Simple Neurite Tracaer (https://github.com/morphonets/SNT). Cellular protein colocalization was analyzed with Fiji plugin Colocalization Colormap (https://sites.google.com/site/colocalizationcolormap/home) and EzColocalization (https://github.com/DrHanLim/EzColocalization). Particle tracking and size quantification were analyzed with Fiji plugin MOSAIC Particle Tracker 2D/3D (https://github.com/mosaic-group/MosaicSuite) and ComDet (https://github.com/ekatrukha/ComDet/).<br><br>Data was imported and organized in R-Studio (Version 1.4.1103) with R packages stats::ts, tsibble::as_tsibble and data.table::data.table. We used R package forecast::ma for moving average smoothening of time-series data. Differential profiles were generated with function R base diff. Pearson's correlation coefficient was computed using the R package stats::ccf. Descriptive statistic analysis was done with R package rstatix::get_summary_stats. We used R package lomb::lsp to calculate the dominant frequency of the time-series data.<br>The custom R and Python scripts used in the study are deposited in https://github.com/darkbreaker0/Arp3_neuronal_polarization_2026 and https://doi.org/10.5281/zenodo.20118606<br><br>The custom ImageJ macro used for generating kymographs, extracting neurite tip positions and protein intensities was deposited in https:/ github.com/darkbreaker0/IJ_NeuriteGrowthScript and https://doi.org/10.5281/zenodo.20118606

We used IMOD (Version 4.5; https://bio3d.colorado.edu/imod/) for tilt series alignment and tomogram reconstruction. Code for actin filament tracking a was published in ref #111.

The code used for polarity determination of the actin filament in tomograms is deposited in https://doi.org/10.5281/zenodo.20081074.

We used the open-source software YAPiC (https://yapic.github.io) and Ilastik software (https://ilastik.org) for machine learning-assisted image segmentation.

For manuscripts utilizing custom algorithms or software that are central to the research but not yet described in published literature, software must be made available to editors and reviewers. We strongly encourage code deposition in a community repository (e.g. GitHub). See the Nature Portfolio guidelines for submitting code & software for further information.

Data

Policy information about availability of data

All manuscripts must include a data availability statement. This statement should provide the following information, where applicable:

- Accession codes, unique identifiers, or web links for publicly available datasets
- A description of any restrictions on data availability
- For clinical datasets or third party data, please ensure that the statement adheres to our policy

We provided the quantified data underlying the graphs of figures in spreadsheet form (.xlsx) as Source Data.
The raw data of the representative images were deposited in https://doi.org/10.5281/zenodo.20118606
Due to large file size of raw image data and the processed data used in the analyses that generated the graphs, we archived the files in the read-only file archive in DZNE institute. We provide raw data files upon request. The request will be fulfilled by the lead contact, Frank Bradke (frank.bradke@dzne.de).

Research involving human participants, their data, or biological material

Policy information about studies with human participants or human data. See also policy information about sex, gender (identity/presentation), and sexual orientation and race, ethnicity and racism.

Reporting on sex and gender	N/A
Reporting on race, ethnicity, or other socially relevant groupings	N/A
Population characteristics	N/A
Recruitment	N/A
Ethics oversight	N/A

Note that full information on the approval of the study protocol must also be provided in the manuscript.

Field-specific reporting

Please select the one below that is the best fit for your research. If you are not sure, read the appropriate sections before making your selection.

☒ Life sciences ☐ Behavioural & social sciences ☐ Ecological, evolutionary & environmental sciences

For a reference copy of the document with all sections, see nature.com/documents/nr-reporting-summary-flat.pdf

Life sciences study design

All studies must disclose on these points even when the disclosure is negative.

Sample size	Sample sizes were not predetermined by formal power analysis. For each experimental paradigm, sample sizes were chosen based on (i) established practice in comparable studies of neuronal polarization and live-cell imaging in our field (ref #8, #49, #83), (ii) the practical throughput of each technique (ref #8, #28, #83), and (iii) ensuring sufficient biological replication to assess reproducibility across independent experiments.<br>For high-throughput assays such as morphological scoring of fixed neurons and live-cell imaging, sample sizes typically ranged from tens to hundreds of cells per condition, examined across at least three independent biological replicates (independent dissections, cultures, or animals).<br>For lower-throughput assays such as optogenetic manipulation, local perfusion, EM tomogram analysis or in utero electroporation, sample sizes were proportionally smaller but always included a minimum of three independent biological replicates (independent dissections, cultures, or animals). The exact sample size, number of independent biological replicates, and statistical test used are specified in each figure legend.
Data exclusions	We considered data points with values greater than 3 standard deviations of the mean as outliers. This exclusion criteria were not pre-

Data exclusions	established but applied on data where normal distribution was expected but obviously skewed by the outliers. These outliers were mostly caused by few cells with abnormal morphologies or artifacts induced by very high overexpression of proteins.
Replication	The exact number of replicates for each experiment has been indicated in the figure legends and supplementary information. Experiments were replicated 3 or more times, with similar results observed each time. Independent dams were used to isolate the embryos, their brains, and the neurons in each independent experiments.
Randomization	The mice, the neurons and the brains isolated from the mice, were randomized into treatment and control groups for experiments using pharmacological agents.
Blinding	Investigators were not blinded to group allocation during data collection and analysis, as data collection and analysis were performed by the same individual assigning the groups. Wherever possible findings (e.g., neurite length, neurite growth velocities, colocalizations, etc,.) were analyzed in an unbiased manner by the use of automated Fiji and custom analysis codes in R.

Reporting for specific materials, systems and methods

We require information from authors about some types of materials, experimental systems and methods used in many studies. Here, indicate whether each material, system or method listed is relevant to your study. If you are not sure if a list item applies to your research, read the appropriate section before selecting a response.

Materials & experimental systems

n/a	Involved in the study
☐	☒ Antibodies
☒	☐ Eukaryotic cell lines
☒	☐ Palaeontology and archaeology
☐	☒ Animals and other organisms
☒	☐ Clinical data
☒	☐ Dual use research of concern
☒	☐ Plants

Methods

n/a	Involved in the study
☒	☐ ChIP-seq
☒	☐ Flow cytometry
☒	☐ MRI-based neuroimaging

Antibodies

Antibodies used	For immunocytochemistry: anti-Tau1 (1:1000; MAB3420, Millipore), anti-tubulin-β3 (1:2500; T2200, Sigma), anti-myosin IIb (1:1000, 8824, Cell Signaling Technology), anti-Arp3 (1:500, A5979, Sigma-Aldrich; 1:200, 0727-2, Millipore), anti-GFP (1:1000; Abcam ab13970). Secondary antibodies: Alexa Fluor 405 anti-rabbit (1:1000; A31556, Invitrogen), Alexa Fluor 555 anti-mouse (1:1000; A21422, Invitrogen), Alexa Fluor 488 anti-rabbit (1:1000; A11034, Invitrogen), Alexa Fluor 488 anti-chicken (1:1000; A11039, Invitrogen), Alexa Fluor 594 anti-rabbit (1:500; A-21207, Invitrogen), Alexa Fluor 594 anti-mouse (1:500; A-11032, Invitrogen), Phalloidin-Atto 647N (1:2500; 65906, Sigma) and Phalloidin-Alexa Fluor 647Plus (1:400; A30107, Thermo).<br>For western blot: anti-Arp3 (1:1500; 07-272, Millipore), anti-Arp2 (1:1500; 5614S, Cell Signalling), anti-ARPC1a (1:500; HPA004334, Sigma), anti-ARPC2 (1:1000; 07-227, Millipore), anti-ARPC4 (1:400; sc-68394, Santa Cruz), anti-ARPC5 (1:400; sc-166760, Santa Cruz), anti-phospho-MRLC (1:1000, ab2480, Abcam; 1:1000, 3675, Cell Signaling), anti-MRLC (1:800, PA5-17624, ThermoFisher; 1:1000, 3672, Cell Signaling), GAPDH (1:10,000; ACR001P, Acris). Secondary antibodies: StrepMAB-Classic-HRP (1:20,000; 2-1509-001, IBA Lifescience), anti-mouse-HRP (1:20,000; 31432, ThermoFisher), anti-rabbit-HRP (1:20,000; 31458, ThermoFisher).
Validation	The detail info of the primary antibodies and their validation is uploaded as Supplementary Table 1.

Animals and other research organisms

Policy information about studies involving animals; ARRIVE guidelines recommended for reporting animal research, and Sex and Gender in Research

Laboratory animals	We used E12.5-E17.5 embryos of Mus musculus, C57BL/6J. For breeding and the generation of embryos we used 3-8 months old Nestin-Cre tg/-, Actr3 fl/wt line, Actr3 fl/fl line and Actr3 fl/- line, with C57BL/6J genetic background. The genotypes of all mice used in this study were verified with PCR.<br>The animals were housed in groups of up to five in individually ventilated cages (IVC) from the Tecniplast Green Line. The following parameters apply: 12-hour light/12-hour dark cycle (6 a.m.–6 p.m./6 p.m.–6 a.m.); 22°C temperature, 60% humidity. For environmental enrichment, nestlets for nest-building and red-colored hiding places (houses, tubes) are provided.
Wild animals	The study did not involve wild animals.
Reporting on sex	This study did not consider effects attributed by sex. The sex of either the embryos or newborn pups was never determined in this study resulting in a random mix of both sexes in all related experiments. Cells from all isolated embryos were pooled, therefore, cultures always contained a random mixture of both sexes.
Field-collected samples	The study did not involve samples collected from the field.

Ethics oversight	All animal experiments were performed in accordance with the Animal Welfare Act and the guidelines of the Landesamt für Natur, Umwelt und Verbraucherschutz (LAVE), North Rhine–Westphalia, Germany.

Note that full information on the approval of the study protocol must also be provided in the manuscript.

Plants

Seed stocks	N/A
Novel plant genotypes	N/A
Authentication	N/A
