# Supplementary material for: An intrinsic cytoskeletal oscillator establishes neuronal polarity
Source: Nature. 2026 Jul 8;657(8130):213–25. doi: 10.1038/s41586-026-10755-6 (PMC13538037; doi:10.1038/s41586-026-10755-6)
Supplement: Supplementary file 3 [file 41586_2026_10755_MOESM4_ESM.pdf]

nature portfolio

Peer Review File

An intrinsic cytoskeletal oscillator establishes neuronal polarity

Corresponding Author: Professor Frank Bradke

Any redactions in this file are there to maintain patient confidentiality, the confidentiality of unpublished data, or to remove third-party material.

This file contains all reviewer reports in order by version, followed by all author rebuttals in order by version.

Version 0:

Reviewer comments:

Referee #1

(Remarks to the Author)
Lin et al. perform time-lapse analysis of dissociated hippocampal neurons and used knockout mice to study the role of Arp2/3 in neuritogenesis and the development of neuronal polarity. Their data suggest that neurites oscillate between extension and retraction phases and that these oscillations are orchestrated by waves of F-actin propagating from the soma to neurites. They also propose a “tensegrity” model whereby actin branching in the soma releases actomyosin and cell tension and ultimately induces axon outgrowth. The work is interesting as understanding how neuronal polarity is established is one of the key question in developmental neurobiology. Unfortunately, many of the data do not support the claims and model.

One important concern is that movies are stopped before symmetry breaking and before neuronal polarity is established. The axons are never seen (Maybe at the exception of movie S7 and for one neuron on figure 4i). Why didn’t they image for a longer period or at a later stage? This is puzzling as using the same model, others were able to precisely monitor protein level variations in nascent axons (Jacobson et al. Neuron 2006; Twelvetrees et al., Neuron 2016).
See for instance 2 movies from the Jacobson paper
https://www.cell.com/cms/10.1016/j.neuron.2006.02.005/attachment/e3be2568-9693-43e5-a6fb-5a9f2da2b998/mmc7.mp4
https://www.cell.com/cms/10.1016/j.neuron.2006.02.005/attachment/5a68147a-890b-491b-9458-9ac3e6e93c1d/mmc9.mp4

These earlier studies (that, surprisingly, are not cited) also showed that kinesin-1 and dynein accumulate in nascent axons. Therefore it would have been important to use these tools to confirm the axonal identity of the observed neuronal processes and truly correlate the described phenomena with axon initiation.
For many movies, it is difficult to see what the author claim in the text. For instance on Movie S5, the neuron looks already polarized (a larger and longer neurite on the top) at the start and the polarity does not change after PA-Rac1 is activated in the nucleus. It is not known if this process will become the axons. Why does the wave do not propagate in neurites that are closer to the site of PA-Rac1 activation? Thus, PA-Rac1 has an effect, but I don’t see an axon being formed.

All the evidence suggesting that variations of Arp2/3 levels in soma and neurites matters for axon specification are indirect. The authors do not directly alter the local concentration of Arp2/3 in growth cones or cell bodies. Inhibitors, such as CK-666 (and nocodazole) are just bath applied, homogeneously. They would need to apply it (using a micropipette or constructs) more locally to better support the proposed model. They would also need to directly act on Arp2/3 (at this point only Pa-Rac1 local concentration is modulated).

Importantly, the authors seem to ignore many previous publications that have already addressed Arp2/3 function in axonogenesis and growth cones using either CK-666 or other inhibitors. I am only citing some.
Strasser et al. Neuron 2004
Ikeno and Konishi, Neurochemical Journal, 2020, not cited
Therefore, the novelty is questionable.

As mentioned before, they do not mention the classic work of Jacobson et al., 2006, who showed that hippocampal neurite

extension and oscillations are correlated with kinesin-1 levels and that it accumulates in the future axon. A phase of oscillatory neurite outgrowth preceding axon specification has already been described and associated to a stabilization of Kinesin-1 in the "winning" growth cone.

The authors "hypothesized that microtubule-actomyosin interactions build a tensegrity system, whereby actomyosin contractility along the whole neurite constantly applies force to microtubules, which bear tension". This is interesting but they did not measure tension, forces and their evolution along the soma and neurites and this would be required to support the proposed model (they could use FRET probes or optic tweezers).

In addition, although their work shows that intrinsic processes influence axon outgrowth, neurons are not isolated when in an in vivo context. ECM and other axon guidance cues will probably change the dynamics of the described processes and should also be integrated in the proposed model. In other words, the physiological relevance of the model, and the relative contribution of intrinsic and extrinsic factors in axon specification is still unclear at this stage.

The Arp3 cKO brain seems extremely affected. Some of the forebrain appears missing, cortex is much thinner ventricles are enlarged. It is most likely that neurogenesis, possibly neuronal migration and survival are altered. The authors used a Nestin-Cre driver that is almost ubiquitously expressed in neural stem cells from early stages of development, well before cortical neurons extend axons. They did not attempt to target a small fraction of cortical neurons using electroporation of Cre and the observed defect in slices could be non-cell autonomous. neurons look abnormal. Again, they did no capture nascent axonas and should perform live imaging as done before to study the emergence of pyramidal neuron polarity and axons (see for example Namba et al., Neuron 2014; 10.1016/j.neuron.2013.12.015)
Here, many things could be affected by Arp2/3 deletion in vivo.

Other points

"Before a single axon forms, multiple neurites emanate from the soma" are the authors confident that this applies to all neurons? I am not so sure as in many systems (retina, brainstem) neurons adopt a bipolar morphology as soon as they become postmitotic and migrate at the same time their axon elongates. Neurite/dendrite outgrowth occurs later.

The concentrations of CK-666 (150 uM, about 2-5x more than in similar studies) and Blebbistatin (20uM) seem very high. Washout data is shown for CK-666 but not Blebbistatin (why?). How was the dose established and are drugs specific at such concentrations?

Video S12 and Supp Fig.9: Previous work using has already used electron microscopy to show at high resolution the consequence of Arp2/3 inactivation on actin filament bundling in growth cones (see Strasser et al., Neuron 2004; not cited). This work should be discussed. In addition, colors could be used to facilitate the analysis of the tomograms

What is the effect of PA-Rac1 activation in Arp3-/- neurons ?

In the case of Actomyosin, it would be important to monitor or detect the active form, which is phosphoryated.

Line 178: "myosin II negatively regulates Arp2/3". Could Arp2/3 also control myosin II activity since acting on actin meshes will influence MyosinII binding

Movie S8: is it in an axon?

Referee #2

(Remarks to the Author)
The study by Lin et al. addresses the basis by which a single axon is formed in the process of neuronal polarization. Mechanisms underlying axon formation have been explored by the classic studies of Banker, Dotti, Bradke and colleagues, for example, Bradke and Dotti reporting of local actin instability being a key trigger in specifying an axon (Science 1999). Despite many important studies to date, the precise cellular and molecular basis for generating a single axon has remained elusive. Here the authors re-examine the topic and conclude that neuronal polarization to generate an axon depends on a tensegrity system. This mechanism involves an actomyosin network that applies tension to the microtubule-dependent extension process to exert global inhibition of outgrowth in an oscillatory manner, and localized Arp2/3-mediated actin branching breaks the global inhibition to allow a single axon to extend. Technically the study is beautifully done. Nevertheless, despite the wealth of data, the individual questions are not clearly motivated in that the logical progression of experiments is missing or not well justified. This makes the paper a difficult read. At the outset, the authors place an emphasis on extrinsic, growth cone-centered view of neuronal polarity specification (Introduction, lines 64-66). Yet, it is not clear that such has been an established view. For instance, there have been reports, including those from the Bradke lab, highlighting the importance of cell intrinsic signaling involving GSK3 signaling and cytoskeletal dynamics in specifying polarization (e.g. JCB 2008; Curr Biol 2008). In the end, how oscillatory neurite growth is coupled to localized Arp2/3 activation, what might be the trigger and how localized Arp2/3 activation, in turn, overcomes the global inhibition mediated through myosin II are not clear. Altogether, the key conclusions that are implied in the title, are not compellingly supported by

the data shown and the study raises more questions in its present form.

Specific points that require consideration:

Fig 1e-g
Why would one expect a positive correlation (i.e. tug of war) between extension of axon and retraction of other neurites in the first place? This is not particularly intuitive. At least in cultures, cells are growing in 2D, and for an axon to extend, a less dynamic change of other neurites would be expected to provide better overall traction/adherence and stability (do cell bodies remain in one position?).

P.5 text line 5. Comparison of polarization in response to chemotaxis is not the most apt comparison because for chemotaxis, there is a clear extracellular source of directional trigger, and moreover, cell polarization involves translocation of the cell body. The analogy invites confusion.

Ext Fig 2c
Kymographs show that actin spots appear and disappear all of a sudden, even for those at the leading edge, which makes it difficult to infer their translocation. Also the image seems pixellated. Would it be possible to show the kymograph (and/or image) at higher spatio-temporal resolution?

Fig 2e
How does the extent change in somatic F-actin intensity relate to the occurrence of F-actin waves? (i.e. What is monitoring the level of somatic F-actin patches and F-actin waves at the neurite?) Is monomeric actin pool shared between somatic F-actin patches and F-actin waves entering the neurite? Relating to the point above on kymographs, FRAP may show a clearer relationship between somatic F-actin patches and F-actin waves beyond correlations.

Fig 2g-k
Photoactivation of PA-Rac1 may simply reflect a gain of function effect. Inhibition experiments might be more insightful. Having said this, what happens if PA-Rac1 is activated at multiple foci (i.e. more than two)? Would there be a competition for resources required for neurite elongation? If neurite formation is due to random activation of Arp2/3, then one might not expect to see competition. In addition, the spatial effects of PA-Rac1 activation spots relative to the site of neurite extension is not clear, at least from the representative examples/videos shown. Would one expect to see the most proximal neurite to the PA-Rac1 activation site to extend?

Fig 3a
In the LEGI model, it is not clear what is the trigger for a localized relaxation of global inhibition (i.e. local excitation) to allow the outgrowth of a single neurite. How is the preferred neurite specified and/or ends up being preferred?

Fig 4
Related to the point above, how Arp2/3 contributes to the formation of no more than a single neurite extension is not clear here. The analyses of the effects of loss of Arp2/3 presented here is limited to the characterization of axon/the longest neurite that has already formed. In addition, the data shown in extended Figures – the lack of effect of PA-Rac1 activation on F-actin dynamics and neurite length – are important in clarifying the role of Arp2/3 and should be presented as main data.

Ext Fig 10f,g
That CK-666 treatment reduces the length of minor neurites despite a lack of effect on the longest neurite in DIV-3 cultures is an important point and should be presented in the main figure. Could the authors show representative images of experiments in DIV-3 neurons? Does photoactivating PA-Rac1 in DIV-3 neurons enable short neurites to elongate? Under such conditions, what happens to the longest neurite?

Fig 5d-g
It is not clear how the experiments here relate to the stability of axon (i.e. resistance to retraction) to Arp2/3 inhibition shown at DIV3. 48 h post-plating would imply that the cultures are DIV2, in which case, are axons already resistant to CK-666 treatment at 48 h post-plating? How about the behavior of axons to taxol treatment in DIV-3 neurons with or without CK-666? Terminology (DIV or post-plating hours) should be consistent throughout the manuscript. Also, does taxol treatment in DIV-3 neurons have any effect on the lengths of minor neurites? That is, given that taxol promotes the formation of multiple axons, if a single axon is already specified, then does the cell still have a capacity to turn the other minor neurites into axons?

Ext Fig 10l-n
Following the washout of CK-666, does the previously longest neurite again become the longest or do other neurites extend further? Also, it would be informative to compare the behavior of EB3 in DIV1 vs DIV3 cultures where the longest neurite is resistant to CK-666 treatment. Does EB3 remain stable in DIV3 axons?

In the model where local Arp2/3 activity breaks the symmetry of global inhibition and induces a single axon, focal activation of Arp2/3 is a key process. To this end, is it possible to deliver CK-666 locally and examine its effect, perhaps coupled to variously positioned local photoactivation of PA-Rac1?

Fig 5k-m
Blebbistatin treatment is for 24 hours, and the slow timing of drug inhibition that is longer than the neurite outgrowth events

complicates the data interpretation.

Referee #3

(Remarks to the Author)
In this paper by Lin et al., the authors re-examine the mechanisms of cell autonomous polarization of axons and dendrites that developing cortical neurons exhibit in vitro. It has been known for decades that cortical and hippocampal neurons will spontaneously polarize with a single long axon and multiple short dendrites over several well-defined stages after days in vitro. Previous work suggested that long-range signals originating from terminal growth cones determined which neurite became the axon. While in vivo there is likely an environmental cue that directs axons since they consistently extend toward the ventricle, how cortical neurons spontaneously polarize without external cues remains a mystery. This paper challenges current dogma by suggesting that axon outgrowth symmetry is broken by oscillatory waves of Arp2/3 driven actin polymerization originating in the cell body. This is observed as traveling waves of F-actin that extend up axons as described previously. Furthermore, the authors suggest that traveling branched actin networks relax acto-myosin contractility when they reach the terminal to promote axon extension.

While the experiments performed in this paper are elegant, cutting-edge, well-controlled and beautifully presented, it is not clear to me whether the conclusions reached are sufficiently novel for publication in Nature. It seems to me that a more specialized cell biology focused journal would be more appropriate. One concern is the relevance of the mechanisms observed in vitro to how cortical neurons naturally polarize in vivo. As cortical neurons migrate along radial glial fibers and stereotypically extend their axons toward the ventricular zone during migration, it is not clear how directly comparable the processes observed on a homogenous PDL substratum in 2D cell culture. While the authors do show a similar correlation of extension and retraction in neurons in cortical slice culture, this one comparison does not prove the same mechanisms are involved (e.g are actin waves observed in vivo?). As environmental factors likely guide polarization in vivo, it seems likely that long-range signals initiated at axon terminals could contribute to polarization. Moreover, previous publications do suggest that paracrine signals released from terminals provide some local excitatory feedback signals that influence distant neurites (Takano et al., 2018). It is not clear how these findings fit with those described here.

This in vitro model systems has be used for decades to understand neuronal polarization and it does provide useful cell biological information about the dynamic regulation of the cytoskeleton free from confounding variables. In this regard, the most significant conclusion reached by these authors in my opinion is the novel notion that branched networks of actin filaments somehow relax actomyosin contractility. While this is an interesting idea, the authors provide no mechanistic or biophysical explanation how this occurs. This conclusion is reached largely from observations of F-actin, Myo-II and MRLC distributions and correlation with actin waves and axon extension, together with specific manipulations of Arp2/3 and myosin activity. To reach this conclusion I believe more direct biophysical or molecular measurements would need to be made. For example, if Arp2/3 mediated F-actin waves suppress myosin activity, would one expect to see reduced p-MRLC (active) within traveling branched networks? Better yet, if a live biosensor for myosin-II activity could be used to correlate dynamic distributions with different actin filament networks, this would provide more direct comparisons. The authors did measure retrograde flow, which is an indirect read-out of myosin II activity. However, they did not compare flow rates of retracting versus extending neurites, which should correlate with Arp2/3 content and branched F-actin. However, these approaches still would not provide a mechanistic understanding how branched actin networks influence myosin-II activity. Reconstitution of branched actin and myosin II-driven F-actin sliding in a cell-free system may be necessary to conclusively demonstrate that this type of mechanism is involved.

Version 1:

Reviewer comments:

Referee #1

(Remarks to the Author)
In their revised manuscript, the authors have addressed all my initial concerns and added an impressive amount of new data. The new results on the local activation/inhibition of Arp2/3 are quite convincing and support their model.
I would suggest to say on the opening frame of each movie, to which figure panel it is related and also provide the movie number. It took me quite some time to find out.

Referee #2

(Remarks to the Author)
The revised manuscript by Lin and colleagues have been significantly improved with additional experiments and careful editing. Extensive sets of new data provide strong support for the claim that cell-intrinsic oscillatory actin cytoskeletal dynamics underlie the formation of a single axon in a developing neuron with further mechanistic insights, and validation in cortical brain slice cultures also adds strength. The authors have suitably addressed my prior concerns in the revision. The main findings would be of broad interest.

Minor comments:

Figure 1d is redundant with panel b. It would be more informative to show an inset of a representative time window with an expanded time scale to highlight the sequential extension and retraction events by the labelled neurites.

Line 98. When introducing the use of cortical slices, it should be made clear that the preparation refers to organotypic culture and/or that slices have been cultured for 30 h. The clarification is not only important in interpreting the data, but also in avoiding the confusion since the authors also use acutely sliced preparation (if I understood it correctly) referred to as transections in other experiments (e.g. Fig 4e-h).

Referee #3

(Remarks to the Author)
I have re-reviewed the paper by Lin et al., and I am afraid my opinion has not changed. This is a very nice cell biological study that describes details of neuron polarization processes. The most impactful conclusion according to authors description is that a “soma-initiated oscillatory program primes axon selection”, which contrasts with the growth cone centric held view previously regarding polarization. This conclusion is based on extensive analysis of live cell behaviors together with pharmacological and molecular manipulations. While this study is extremely detailed, its overall impact, combined with several minor concerns, reduces my enthusiasm for publication in Nature. I will list a few concerns below.

Is it stated on line 101 “Strikingly, we found that upon wave formation, adjacent neurites rapidly retracted”. I do not see any change of adjacent neurites after wave enters primary neurite in figure or movie. Moreover, the “waves” in cell bodies appear subtle (some appear to be blebs without increased lifeact intensity). It is not clear how these are subjectively scored as waves. Moreover and most importantly, often waves appear to form first in proximal processes near the cell body (Fig 2a). Are these also considered cell soma waves? All examples shown were of developing neurons that already had minor processes. Prior to any minor process formation, do neuronal cell bodies show many waves that initiate minor processes?

Figure 2 correlates sub-cellular actin punta (patches) with wave formation. Small puncta appear all over the cell body with no clear bias toward where a wave forms. Also, in figure 2a, waves initiate within the proximal neurite, so it is not clear how this correlates with actin patches.

Figure 3 shows that Arp2/3 is responsible for F-actin waves, which is expected as Arp2/3 is known to promote branched actin networks.

Figure 4 is nice, as it shows neuronal polarization is disrupted in Arp3 mutants in vivo, although this is not unexpected.

On line 174 the others state that “the original polarity was largely preserved upon recovery” after washout of CK666. How does the wave model account for this finding?

Finally, the authors make the blanket statement that “Indeed, growth cones of embryonic CNS neurons do not pull on 3-dimensional soft matrices to move forward”. This may be true for some CNS neurons in some circumstances, but it is highly likely that some CNS neurons (eg retinal ganglion cells and spinal commissural neurons) extend by traditional mesenchymal based cell migration) by pulling on soft substrates (see papers by David Odde (eg Traction Dynamics of Filopodia on Compliant Substrates) and others).

Open Access This Peer Review File is licensed under a Creative Commons Attribution 4.0 International License, which permits use, sharing, adaptation, distribution and reproduction in any medium or format, as long as you give appropriate credit to the original author(s) and the source, provide a link to the Creative Commons license, and indicate if changes were made.
In cases where reviewers are anonymous, credit should be given to 'Anonymous Referee' and the source.
The images or other third party material in this Peer Review File are included in the article's Creative Commons license, unless indicated otherwise in a credit line to the material. If material is not included in the article's Creative Commons license and your intended use is not permitted by statutory regulation or exceeds the permitted use, you will need to obtain permission directly from the copyright holder.
To view a copy of this license, visit https://creativecommons.org/licenses/by/4.0/

Response to the Reviewers' comments on Nature 2023-11-21494:

"Antagonistic actin networks coordinate neuronal polarization though oscillatory neurite growth"

We thank all three reviewers for their constructive criticism, which has led to an improved manuscript. In the following, we address the reviewers' comments point-by-point.

The reviewers' comments are in blue font, our responses are in black font, and new text added to the manuscript is in black italic font.

Reviewer #1:

Lin et al. perform time-lapse analysis of dissociated hippocampal neurons and used knockout mice to study the role of Arp2/3 in neuritogenesis and the development of neuronal polarity. Their data suggest that neurites oscillate between extension and retraction phases and that these oscillations are orchestrated by waves of F-actin propagating from the soma to neurites. They also propose a "tensegrity" model whereby actin branching in the soma releases actomyosin and cell tension and ultimately induces axon outgrowth. The work is interesting as understanding how neuronal polarity is established is one of the key question in developmental neurobiology. Unfortunately, many of the data do not support the claims and model.

1) One important concern is that movies are stopped before symmetry breaking and before neuronal polarity is established. The axons are never seen (Maybe at the exception of movie S7 and for one neuron on figure 4i). Why didn't they image for a longer period or at a later stage? This is puzzling as using the same model, others were able to precisely monitor protein level variations in nascent axons (Jacobson et al. Neuron 2006; Twelvetrees et al., Neuron 2016). See for instance 2 movies from the Jacobson paper

https://www.cell.com/cms/10.1016/j.neuron.2006.02.005/attachment/e3be2568-9693-43e5-a6fb-5a9f2da2b998/mmc7.mp4

https://www.cell.com/cms/10.1016/j.neuron.2006.02.005/attachment/5a68147a-890b-491b-9458-9ac3e6e93c1d/mmc9.mp4

We thank the reviewer for this important comment. In the initial submission, our time-lapse videos focused primarily on the pre-polarization stage, which is where oscillatory neurite dynamics and actin waves are most prominent. As the reviewer suggested, we have now extended our imaging to include **before, during, and after symmetry breaking**, both *in vitro* and *ex vivo* in cortical slices.

These new experiments clarify the transition from oscillation to polarization and demonstrate that oscillatory growth is mechanistically distinct from the polarization phase itself.

1. Oscillatory stage (before polarization)

When neurons were imaged for longer periods, we observed repeated somatic actin waves, each followed by rapid retraction of neighboring neurites. When a wave propagated into a particular neurite and reached the tip, that neurite underwent transient extension. Importantly, we confirmed this behavior not only in dissociated neurons but also in cortical slice cultures. We added the results in **Fig. 1h-i** and the associated text in the manuscript:

Line 99: "*Consistent with studies showing actin waves in cultured neurons*[1,2]*, unpolarized neurons in cortical slices repeatedly formed actin waves at the soma **(Fig. 1i)**. Strikingly, we found that upon wave formation, adjacent neurites rapidly retracted; the wave-receiving neurite then regrew **(Fig. 1h; Supplementary Video 3)**.*"

2. Polarization stage (symmetry breaking)

With extended long-term time-lapse imaging, we now show that nascent axons grow independently of actin waves. During polarization, the nascent axon receives fewer somatic waves (**Fig. 2g-h)**, shows higher extension rates (**Fig. 2j-k**), and undergoes much shorter retraction episodes (**Fig. 2j-k**). In contrast, minor neurites continue to receive waves and show increased retraction speed (**Fig. 2k**).

These new data are articulated in the manuscript:

Line 119: "*As in cortical slices, the nascent axon in vitro hardly received waves during polarization (**Fig. 2g-h**), while the overall frequency of wave formation per neuron remained constant **(Fig. 2i)**. Concomitantly, the axon showed increased extension rate and duration (**Fig. 2j-k**). By contrast, upon polarization, minor neurites received more waves **(Fig. 2g-h; Supplementary Video 5)**, and their retraction rate increased (**Fig. 2j-k**).*"

3. After polarization (polarity consolidation)

Once polarization was established, axons continued to show reduced retraction rate and duration compared with minor neurites **(Extended Data Fig. 2j-k)**. Moreover, actin waves continued to be absent in axons both in cell culture and in cortical slice culture **(Fig. 1j; Extended Data Fig. 2h-i)**. Consistently, we observed a drastic decrease of growth-actin correlation in axons in comparison with minor neurites **(Extended Data Fig. 2g and m)**.

We added these results in **Extended Data Fig. 2g-m** and the associated text in the manuscript:

Line 123: "*The axon and the minor neurites kept these different characteristics after polarization (**Extended Data Fig. 2h-k; Supplementary Video 6**). Both growth oscillation (**Extended Data Fig. 2l**) and growth-actin correlation were drastically reduced in axons **(Fig. 2l and Extended Data Fig. 2g and m)***".

Taken together, actin waves correlate with neurite growth only during the oscillatory pre-polarization stage. This association disappears during polarization, as axonal growth becomes independent of wave activity. These results further support our conclusion that axons of polarized neurons become resistant to Arp2/3 inhibition: pharmacological Arp2/3 blockade with CK-666 caused retraction only in minor neurites, while axons remained largely unaffected **(Fig. 3f-i; Extended Data Fig. 4d-f)**.

2) These earlier studies also showed that kinesin-1 and dynein accumulate in nascent axons. Therefore it would have been important to use these tools to confirm the axonal identity of the observed neuronal processes and truly correlate the described phenomena with axon initiation.

As the reviewer suggested, we examined the relationship between kinesin-1 and neurite growth by tracking the translocation of KIF5C, following established approaches[3,4]. Using a truncated KIF5C construct that serves as a marker of "transient axon" identity[5], we found that changes in KIF5C intensity at neurite tips were positively correlated with changes in extension velocity. Consistent with this, KIF5C levels were significantly higher in extending neurites compared with pausing or retracting neurites. These results indicate that during oscillatory growth, kinesin-1 enrichment is tightly associated with actin fluctuations and reliably marks neurites undergoing axon-like extension. We have added these data to the revised manuscript (**Extended Data Fig. 3m-o**) and included the following text:

Line 152: "*Consistent with previous work[3,5,6], extending neurites were also enriched in an exogenously expressed truncated kinesin KIF5C construct, a marker of nascent axons[5] (**Extended Data Fig. 3m-o**), and exhibited......*"

Of additional note for the reviewer: We think that the reviewer was absolutely right about this point and it improved our manuscript. Although Jacobson et al. (2006) actually reported that there was "*no correlation between the minor neurites undergoing growth spurts and those with accumulations of Kinesin-1*" and suggested that "*stage growth and molecular differentiation are indeed separable events*" (verbatim from Jacobson et al. 2006), their time-lapse imaging had a low temporal resolution of one frame per 15 minutes. Because the extension-retraction oscillation period is ~20-30 minutes, such resolution is insufficient to capture these dynamics. Our imaging at one frame every two minutes allowed us to resolve these transient relationships, and we now directly observe the correlation anticipated by the reviewer.

Together, these new results confirm that KIF5C enrichment reliably marks the nascent axon during the oscillatory stage and supports its use to identify axon initiation in our study.

3) For many movies, it is difficult to see what the author claim in the text. For instance on Movie S5, the neuron looks already polarized (a larger and longer neurite on the top) at the start and the polarity does not change after PA-Rac1 is activated in the nucleus. It is not known if this process will become the axons. Why

does the wave do not propagate in neurites that are closer to the site of PA-Rac1 activation? Thus, PA-Rac1 has an effect, but I don't see an axon being formed.

To improve the clarity of presentation, we revised the figures and videos by adding labeling and annotations and optimizing color coding. We also revised the text for better clarity and added schemes illustrating experimental design and working models to assist digestion of the manuscript.

The indicated video (now **Supplementary Video 17** and **Fig. 3m**) shows that activating photoactivatable (PA)-Rac1 at the soma induced lamellipodia as one nascent actin wave at the soma periphery, which was associated with retraction of adjacent neurites. The wave then propagated into the neurite closest to the activation site, consistent with our observation of native wave formation and the following propagation. We now enlarged the dots marking the activation site and added arrowheads to assist tracking of wave translocation in **Supplementary Video 17**.

We agree with the reviewer that PA-Rac1 activation at the soma does not trigger axon formation. This is in fact a key mechanistic result and is fully consistent with the observation in cortical slices and in cultured neurons that axon formation and growth was independent of actin waves **(Fig.1j and 2g-k; Extended Data Fig. 2h-k)**; wave formation was associated with retraction of minor neurites **(Fig.1h, 1 and 2g-k; Extended Data Fig. 2h-k)**. In contrast, activating PA-Rac1 at the growth cone induced transient neurite extension. These results demonstrate location-dependent effects of Arp2/3 activation on neurite growth and recapitulate the oscillatory growth and the local actin-growth correlations observed in native cells.

4) All the evidence suggesting that variations of Arp2/3 levels in soma and neurites matters for axon specification are indirect. The authors do not directly alter the local concentration of Arp2/3 in growth cones or cell bodies. Inhibitors, such as CK-666 (and nocodazole) are just bath applied, homogeneously. They would need to apply it (using a micropipette or constructs) more locally to better support the proposed model. They would also need to directly act on Arp2/3 (at this point only Pa-Rac1 local concentration is modulated).

We agree that directly manipulating Arp2/3 activity at specific subcellular locations is important to determine whether wave initiation depends on somatic context or simply on local Arp2/3 availability. In response, we have now performed local pharmacological inactivation of Arp2/3, complementing our existing local PA-Rac1 activation experiments.

To locally perturb Arp2/3 function, we adapted a controlled perfusion setup we previously developed[7]. By combining an ejection capillary with an aspiration capillary, we created a perfusion field with maximal diameter of 80 µm for 90 mins. CK-666 was perfused together with the fluorescent tracer Fast green to continuously visualize the perfused region **(Fig. 3o-p)**.

Local perfusion of CK-666 over the soma and proximal neurites caused selective retraction of neurites within the perfused region **(Extended Data Fig. 5i)**. In contrast, neurites outside the perfusion area remained stable and, in more than half of the neurons (14/25 vs. 1/19 in DMSO controls), these neurites generated retrograde actin waves originating from their growth cones **(Fig. 3p-r)**. These waves propagated toward the soma but disappeared upon entering the CK-666 exposed region.

These results demonstrate that wave initiation requires local Arp2/3 activity and is not restricted to the soma, directly addressing the reviewer's concern. We have added this in **Fig. 3p-r**, **Extended Data Fig. 5i** and to the manuscript text:

Line 205: "*Conversely, locally inactivating Arp2/3 by perfusing CK-666[7] induced spatially asymmetric effects (**Fig. 3o-r and Supplementary Video 18**). When CK-666 was perfused over the soma and short neurites, these neurites retracted, whereas neurites outside the perfused region or in vehicle treated controls did not (**Extended Data Fig. 5i**). Notably, wave initiation depended on local Arp2/3 activity and was nor restricted to the soma: in more than half of the neurons, a neurite located outside the perfused area generated retrograde actin waves originating from its growth cone (14/25 versus 1/19 in DMSO control; **Fig. 3p-r**). These waves propagated toward the soma but dissipated upon reaching the CK-666-exposed region.*"

Moreover, our data showed that myosin II activity constrains neurite extension and drives retraction: inhibiting myosin II with *para*-aminoblebbistatin increased the extension induced by PA-Rac1 activation at

the neurite tip, and abolished the retraction induced by PA-Rac1 activation at the soma. Consistently, local perfusion of *para*-aminoblebbistatin induced extension specifically in neurites within the perfused region, whereas neurites outside the perfusion area did not extend.

We added the results in **Extended Data Fig. 7a-f** and text**:**

Line 240: "*Consistent with the idea that global myosin II activity restrains neurite extension and drives retraction following Arp2/3 activation, inhibiting myosin II with para-aminoblebbistatin enhanced the extension induced by PA-Rac1 activation at the neurite tip (**Extended Data Fig. 7a-b**). Moreover, para-aminoblebbistatin abolished the retraction triggered by PA-Rac1 activation at the soma (**Extended Data Fig. 7c-d**). Consistently, local perfusion of para-aminoblebbistatin at the soma and short neurites induced neurite extension (**Extended Data Fig. 7e-f**).*"

5) Importantly, the authors seem to ignore many previous publications that have already addressed Arp2/3 function in axonogenesis and growth cones using either CK-666 or other inhibitors. I am only citing some. Strasser et al. Neuron 2004; Ikeno and Konishi, Neurochemical Journal, 2020, not cited. Therefore, the novelty is questionable.

We thank the reviewer for highlighting these important studies. Indeed, several groups have examined Arp2/3 function in neurite and axon growth, and their conclusions have been conflicting. Some reports found that Arp2/3 inhibits neurite or axon extension (Strasser et al. 2004; Ikeno and Konishi 2020)[8,9] whereas other studies concluded that Arp2/3 promotes neurite/axon growth (Kunda et al. 2001; Tahirovic et al. 2010; Montenegro-Venegas et al. 2010)[10–12]. These discrepancies have contributed to substantial uncertainty regarding the role of Arp2/3 in axogenesis.

Importantly, all these prior studies relied on molecular manipulations in cultured neurons, including delocalization of Arp2/3 or global pharmacological perturbations, which can alter Arp2/3's spatial distribution or produce non-physiological effects. Resolving this issue necessitated the generation of a physiological loss of function, together with local manipulation of Arp2/3 activity. With this work, we demonstrate that Arp2/3 is a positive physiological regulator of neuronal polarization.

Following reviewer #1's advice, we now pointed out this debate about Arp2/3 in axon growth as a positive or negative regulator in the revised manuscript:

Line 259: "*Paradoxically, Arp2/3 has been reported to both promote and inhibit axon growth in vitro*[8,9,13]*, but these studies relied on molecular manipulations of Arp2/3 that may have altered the complex's localization. To determine the physiological requirement of Arp2/3 for neuronal polarization, we used a genetic loss-of-function approach.*"

6) As mentioned before, they do not mention the classic work of Jacobson et al., 2006, who showed that hippocampal neurite extension and oscillations are correlated with kinesin-1 levels and that it accumulates in the future axon. A phase of oscillatory neurite outgrowth preceding axon specification has already been described and associated to a stabilization of Kinesin-1 in the "winning" growth cone.

We fully agree that kinesin-1 dynamics are closely linked to neurite growth and axon specification and we now explicitly discuss this work in the revised manuscript. As noted in our response to point #2, our new experiments directly show that kinesin-1 (KIF5C) levels correlate with neurite extension during the oscillatory stage and that KIF5C is enriched in neurites that transiently behave like nascent axons (**Extended Data Fig. 3m-o**). Thus, our data support the reviewer's statement that kinesin-1 marks the "transient axon" during symmetry breaking.

As mentioned above, Jacobson et al. (2006) reported no correlation between growth spurts and kinesin-1 enrichment. This difference is likely due to the much lower temporal resolution of their time-lapse imaging. Indeed, Jacobson et al. explicitly noted "*During stage 2 we found no correlation between the minor neurites undergoing growth spurts and those with accumulations of Kinesin-1. This finding suggests that the signaling events lead to minor neurite elongation are different from those that lead to enhanced translocation of Kinesin-1, suggesting that at this stage growth and molecular differentiation are indeed separable events.*" With higher-resolution imaging (1 frame/2-min), we were able to detect correlations that were previously inaccessible.

Collectively, our work reveals that Arp2/3 establishes a cytoskeleton-based activator-inhibitor system with actomyosin networks. This system drives transient polarization and restricts axon number in the first place.

This enables neurons to respond dynamically to a rapidly changing cellular environment during development while retaining the intrinsic polarization program. This intrinsic mechanism operates alongside, and is compatible with, kinesin-1 accumulation and other molecular events described previously. We have now incorporated these points into the Discussion to provide clearer connection to the existing literature and to acknowledge Jacobson et al.'s foundational contributions.

7) The authors "hypothesized that microtubule-actomyosin interactions build a tensegrity system, whereby actomyosin contractility along the whole neurite constantly applies force to microtubules, which bear tension". This is interesting but they did not measure tension, forces and their evolution along the soma and neurites and this would be required to support the proposed model (they could use FRET probes or optic tweezers).

We agree with the reviewer's concern. As this terminology was not essential for our main conclusions and could be misleading without direct biophysical measurements, we have removed the term and any inference of a tensegrity framework from the revised manuscript to avoid overinterpretation.

8) In addition, although their work shows that intrinsic processes influence axon outgrowth, neurons are not isolated when in an in vivo context. ECM and other axon guidance cues will probably change the dynamics of the described processes and should also be integrated in the proposed model. In other words, the physiological relevance of the model, and the relative contribution of intrinsic and extrinsic factors in axon specification is still unclear at this stage.

The Arp3 cKO brain seems extremely affected. Some of the forebrain appears missing, cortex is much thinner ventricles are enlarged. It is most likely that neurogenesis, possibly neuronal migration and survival are altered. The authors used a Nestin-Cre driver that is almost ubiquitously expressed in neural stem cells from early stages of development, well before cortical neurons extend axons. They did not attempt to target a small fraction of cortical neurons using electroporation of Cre and the observed defect in slices could be non-cell autonomous. neurons look abnormal. Again, they did not capture nascent axons and should perform live imaging as done before to study the emergence of pyramidal neuron polarity and axons (see for example Namba et al., Neuron 2014; 10.1016/j.neuron.2013.12.015). Here, many things could be affected by Arp2/3 deletion in vivo.

We thank the reviewer for raising these important points regarding physiological relevance and potential non–cell-autonomous effects in the Nestin-Cre model. We agree that, *in vivo*, neurons experience extracellular cues and interactions with surrounding cells that could influence axon initiation. We therefore performed additional experiments to directly address these concerns:

1. Testing cell-autonomous polarization defects of Arp3 KO neurons in an intact brain environment

To determine whether the axon formation defect in Arp3 conditional knockout neurons reflects a cell-intrinsic requirement rather than alterations in the surrounding tissue, we eliminated Arp3 in a sparse population of cortical neurons while preserving overall brain architecture. We achieved this by *in utero* electroporation (IUE) of a Cre expression plasmid driven by the neuron-specific tubulin-α1 promoter together with a Cre-dependent GFP reporter. As a control, we used Dre recombinase and a Dre-dependent GFP reporter, as previously established[14].

Strikingly, neurons expressing Cre failed to form an axon, despite developing within an otherwise normal cortex. This result demonstrates that loss of Arp2/3 activity is sufficient to abolish the cell-intrinsic polarization mechanism and is not simply a secondary consequence of altered brain morphology in the nestin-Cre line.

We added the result in **Fig. 4g-h** and following text in the revised manuscript:

Line 272: "*To test whether the defect reflects impaired cell-intrinsic polarization, we used tubulin α1 promotor to drive neuron-specific expression of Cre through IUE. As tubulin-α1 promotor starts to express later than the Nestin promoter[15,16], we used Arp3$^{fl/-}$ brains to ensure efficient Arp2/3 depletion. The result consistently showed abrogated axon formation (**Fig. 4g-h**).*"

2. Live imaging of WT neurons in cortical slices

To directly examine the physiological relevance of oscillatory growth and actin waves, we imaged unpolarized and polarized WT neurons in E15.5 cortical slices expressing Lifeact-FP via IUE. The results fully mirrored our cultured neuron findings: unpolarized neurons showed repeated somatic waves, each wave coincided with rapid retraction of adjacent neurites, the neurites receiving the wave transiently regrew, and in polarized neurons, axons did not acquire waves and extended persistently and wave-independently. These data show that the oscillatory-to-polarized transition described *in vitro* also occurs in the physiological tissue environment.

We added the result in **Fig.1h-l**, **Supplementary Video 3 and 4** and the text:

Line 100: "……*unpolarized neurons in cortical slices repeatedly formed actin waves at the soma* ***(Fig. 1i)****. Strikingly, we found that upon wave formation, adjacent neurites rapidly retracted; the wave-receiving neurite then regrew* ***(Fig. 1h; Supplementary Video 3****). In polarized neurons of cortical slices, by contrast, axons acquired no wave and elongated persistently, with extension times four-fold longer than retraction times* ***(Fig 1j-l; Extended Data Fig. 1g; Supplementary Video 4)****.*"

3. Live imaging Arp3 KO neurons in cortical slices

To assess whether Arp3 KO neurons *in vivo* exhibit the same deficits observed *in vitro*, we expressed cell membrane marker LynTM-FP via IUE in WT and Arp3 KO brains and analyzed neurite dynamics in cortical slices. As with *in vitro* culture, Arp3 KO neurons in the slices displayed markedly reduced growth dynamics and impaired polarization. These phenotypes support the conclusion that Arp2/3 is required for dynamic neurite growth *in vivo*. We added these results in **Fig.4n-p** and the text:

Line 288: "*both Arp3 KO cortical slices and cultured Arp3 KO neurons exhibited a reduction of growth dynamics* ***(Fig. 4n-p; Supplementary Videos 24 and 25)***."

Collectively, these new experiments demonstrate the defective polarization autonomy of knocking out Arp3 in only a minority of cortical neurons, visualize actin waves and oscillatory growth in cortical tissue (and thus confirm their physiological relevance *in vivo*), and show that Arp3 KO defects persist in this intact cellular environment. Thus, while extrinsic cues undoubtedly influence axon guidance, our data show that the initiation of neuronal polarity relies on a cell-intrinsic cytoskeletal mechanism governed by Arp2/3.

Responses to the minor points:

9) "Before a single axon forms, multiple neurites emanate from the soma" are the authors confident that this applies to all neurons? I am not so sure as in many systems (retina, brainstem) neurons adopt a bipolar morphology as soon as they become postmitotic and migrate at the same time their axon elongates. Neurite/dendrite outgrowth occurs later.

Indeed, not all neuronal types form multiple neurites prior to axon initiation. To avoid overgeneralization, we now explicitly restrict our statements to cortical pyramidal neurons, which are the neuronal type investigated in this study and for which the "multiple neurites before symmetry breaking" paradigm applies. The text has been revised:

Line 46: "*Cortical pyramidal neurons establish polarity by breaking cellular symmetry to form a single axon and multiple dendrites*[17,18]"

Line 65: "*Using cortical pyramidal neurons, we showed that the antagonistic interplay between Arp2/3 and actomyosin drives oscillatory neurite growth prior to polarization.*"

10) The concentrations of CK-666 (150 uM, about 2-5x more than in similar studies) and Blebbistatin (20uM) seem very high. Washout data is shown for CK-666 but not Blebbistatin (why?). How was the dose established and are drugs specific at such concentrations?

We did the suggested serial dosage titration and washout experiments and presented the results in **Extended Data Fig. 12a-d**. The results show that the selected dosages of blebbistatin produce robust, reproducible effects on neurite growth without compromising cell viability. We based our working concentrations on commonly used doses in prior studies: studies addressing the role of myosin II in neurons

used 50 µM blebbistatin[19,20], which was more than double of the dosage used in our study. Former works characterizing the Arp2/3 inhibitor CK-666 use working concentrations ranging from 50 – 300 µM[21,22]. Within this range, the concentration of CK-666 used in our study was 150 µM.

We added these references in Materials and Methods to clarify that the selected concentrations of CK-666 and blebbistatin are well supported by previous works:

Line 675 (Materials and Methods): "*Based on the published works[4,19,22,23], the final concentration of each chemical was as followed: 20 µM blebbistatin, 40 µM para-Blebbistatin, 100 and 150 µM CK-666, 5 nM Taxol, 75 nM nocodazole*".

11) Video S12 and Supp Fig.9: Previous work using has already used electron microscopy to show at high resolution the consequence of Arp2/3 inactivation on actin filament bundling in growth cones (see Strasser et al., Neuron 2004; not cited). This work should be discussed. In addition, colors could be used to facilitate the analysis of the tomograms.

We thank reviewer #1 for pointing out the work by Strasser et al. and the advice on tomogram visualization.

Strasser et al. performed important early ultrastructural analyses of growth cones using the CA peptide to delocalize Arp2/3. Their work demonstrated changes in actin organization upon Arp2/3 perturbation, and we now cite this study in the revised manuscript as part of the background motivating our work. However, the CA-peptide approach alters Arp2/3 localization rather than depleting the full Arp2/3 complex. Consistent with this, Strasser et al. reported that they could not resolve 3D actin architectures or identify branching junctions in their controls, making it difficult to determine the extent to which Arp2/3-mediated branching was specifically disrupted.

In contrast, our study used Arp3 KO neurons, where the depletion of Arp2/3 complex was confirmed with western blot and immunostaining. Our EM analysis resolved 3D actin organizations and identified actin branching points in the WT control growth cones that were absent in the Arp3 KO neurite tips.

As the reviewer suggested, we added the text below and cited this paper as one of the references motivating this work:

Line 259: "*Paradoxically, Arp2/3 has been reported to both promote and inhibit axon growth in vitro[8,9,13], but these studies relied on molecular manipulations of Arp2/3 that may have altered the complex's localization. To determine the physiological requirement of Arp2/3 for neuronal polarization, we used a genetic loss-of-function approach.*"

To facilitate the analysis of tomograms, we color-coded the filaments of extracted models according to the orientations and created orientation maps. We added the results in **Fig. 4q** and **Extended Data Fig. 10f**. In addition, we overlaid the tomograms with filament density heatmaps, together with branching points, as shown in **Extended Data Fig. 10b**.

12) What is the effect of PA-Rac1 activation in Arp3-/- neurons?

In Arp3 KO neurons, PA-Rac1 activation had no detectable effect on either local actin assembly or neurite growth (original Extended Data Fig. 3f-h) further supporting the notion that Rac1 affects the actin cytoskeleton through Arp2/3. For better comparison between experimental groups and the controls, we moved the neurite length quantification including all the controls (Arp3 KO and PA^{C450M}-Rac1) and experimental groups to **Fig. 3n**. The quantification of actin intensity including all the controls and experimental groups is shown in **Extended Data Fig. 5b-c**, and the associated text:

Line 187: "*Activating PA-Rac1 increased local actin assembly, an effect absent in its light-insensitive mutant (C450M) and in Arp3-knockout neurons **(Extended Data Fig. 5b-c)**.*"

13) In the case of Actomyosin, it would be important to monitor or detect the active form, which is phosphorylated.

As suggested, we assessed not only total myosin regulatory light chain (MRLC) but also its phosphorylated, active form, which directly reflects myosin II activity. Western blotting of biochemical neuronal extracts showed that Arp3 KO neurons had elevated level of phospho-MRLC. This is consistent with the idea that the depletion of actin branching increases the dominance of myosin II on the actin networks. We added the result in **Extended Data Fig.9h-i** and the text:

Line 297: "*Consistently, analysis of biochemical extracts of Arp3 KO neurons showed an increase of phospho-MRLC (**Extended Data Fig. 9h-i**), reporting higher myosin II activity*[20]."

14) Line 178: "myosin II negatively regulates Arp2/3". Could Arp2/3 also control myosin II activity since acting on actin meshes will influence Myosin II binding

The reviewer is absolutely right. This is the reason that we also mention this interaction as reciprocal and antagonistic. In brief, Arp2/3 regulate actomyosin networks through two mechanisms. First, Arp2/3 and myosin II compete for limited actin binding sites [24]. Second, actin branching, which is angled at 70 degrees, is geometrically incompatible with an antiparallel network (180 degrees). Therefore, branches interfere with actomyosin alignment and impede actomyosin contractility[25–28]. We further added more info about this molecular interaction in the manuscript in the text:

Line 246: "*Consistent with the view that actin branching disrupts contraction of anti-parallel actomyosin networks*[25–28] *and competes with actin bundling factors for actin binding*[24,29,30]*...*"

15) Movie S8: is it in an axon?

The original Movie S8 (now **Supplementary Video 20**) is a growth cone of an extending neurite of an unpolarized neuron. Now in Supplementary Video 20 we present the full neuron and the zoom-in of the indicated growth cone, which shows dispersion of MRLC patches during extension.

Reviewer #2:

The study by Lin et al. addresses the basis by which a single axon is formed in the process of neuronal polarization. Mechanisms underlying axon formation have been explored by the classic studies of Banker, Dotti, Bradke and colleagues, for example, Bradke and Dotti reporting of local actin instability being a key trigger in specifying an axon (Science 1999). Despite many important studies to date, the precise cellular and molecular basis for generating a single axon has remained elusive. Here the authors re-examine the topic and conclude that neuronal polarization to generate an axon depends on a tensegrity system. This mechanism involves an actomyosin network that applies tension to the microtubule-dependent extension process to exert global inhibition of outgrowth in an oscillatory manner, and localized Arp2/3-mediated actin branching breaks the global inhibition to allow a single axon to extend.

Technically the study is beautifully done. Nevertheless, despite the wealth of data, the individual questions are not clearly motivated in that the logical progression of experiments is missing or not well justified. This makes the paper a difficult read.

We thank the reviewer for the thoughtful overall assessment and for noting that "the work is beautifully done." We appreciate the reviewer's concern that, despite the technical rigor, the manuscript was at times difficult to follow. To address this, we have substantially revised the manuscript to make the central rationale explicit and continuous throughout the Results, integrating the reciprocally antagonistic activator–inhibitor framework as the conceptual backbone guiding each experimental step. We also added schemes illustrating experimental design and working models to assist digestion of the manuscript. Throughout the Results section of the revised manuscript, we refined now the rationale of the experiments and addressed the points as detailed in the following subpoints:

At the outset, the authors place an emphasis on extrinsic, growth cone-centered view of neuronal polarity specification (Introduction, lines 64-66). Yet, it is not clear that such has been an established view. For instance, there have been reports, including those from the Bradke lab, highlighting the importance of cell intrinsic signaling involving GSK3 signaling and cytoskeletal dynamics in specifying polarization (e.g. JCB 2008; Curr Biol 2008).

We thank reviewer #2 for this important comment. We fully agree that cell-intrinsic signaling mechanisms, including those involving GSK3 and cytoskeletal regulation, have been recognized for many years as contributors to neuronal polarity. The point we intended to convey is not that intrinsic signaling was unknown, but that these intrinsic pathways have historically been interpreted within a framework in which the growth cone is viewed as the central organizing site of symmetry breaking. This perspective has been prominent for decades and is reflected in numerous reviews, including our own[18,31–35].

Because growth cones share structural and functional features with the leading edge of motile cells, many models inferred mechanisms by analogy to migratory machinery. Consequently, the growth cone has been widely regarded as the primary "signaling hub" for neuronal polarization[18,32,36].

However, purely growth cone-centered models raise conceptual difficulties. If axon specification were dictated by local growth-cone signaling in response to widely available environmental cues, then multiple neurites would acquire axon identity under uniform exposure. Yet, neurons reliably form only a single axon *in vivo*, despite the developing brain being rich in neurotrophic factors. Moreover the initial site of axon emergence is not specified by the later direction of guided axon extension[37]. Even in controlled *in vitro* settings, when two neurites simultaneously contact laminin, only one becomes the axon[38].

Growth cone-centered models also assume the existence of a long-range inhibitory signal to prevent multiple neurites from adopting axon identity, a mechanism sometimes attributed to RhoA signaling[20,32]. Yet neurons polarize normally even when RhoA signaling is disrupted, calling this assumption into question[39].

To clarify these issues, we have revised the Introduction to better articulate this conceptual gap. The new text reads:

Line 55: "*In this growth-cone-centered view, both neuronal polarity and axon formation would be dictated by the distribution of external guidance molecules, raising the possibility that multiple neurites could adopt axon identity under uniform exposure. However, this model is difficult to reconcile with the fact that neurons reliably form only a single axon in the developing brain, an environment rich in growth-promoting factors, and the initial site of axon emergence is not dictated by the eventual direction of guided axon extension*[37]*. Even when two neurites of a neuron simultaneously contact growth promoting substrates, only one becomes the axon, and the other is restrained*[38]*.*"

In summary, our work provides two key insights that resolve this long-standing puzzle:

1) Neurite growth is not controlled solely by the growth cone. We identify the soma as a central site coordinating neurite growth through Arp2/3-driven actin waves that transiently relax actomyosin contractility in only one neurite at a time.

2) Global actomyosin-mediated inhibition is cell-intrinsic and dominant by default. This constraint does not require extrinsic cues or long-range inhibitory signaling and provides a robust mechanism to ensure that only a single neurite escapes retraction to become the axon.

In the end, how oscillatory neurite growth is coupled to localized Arp2/3 activation, what might be the trigger and how localized Arp2/3 activation, in turn, overcomes the global inhibition mediated through myosin II are not clear. Altogether, the key conclusions that are implied in the title are not compellingly supported by the data shown and the study raises more questions in its present form.

We thank Reviewer #2 for this insightful summary comment. We agree that in the initial submission, the coupling between oscillatory neurite growth, localized Arp2/3 activation, and the mechanism by which Arp2/3 overcomes global myosin II–mediated inhibition was not described as clearly as it could have been. Importantly, the reviewer's suggestions helped us significantly strengthen both the experimental evidence and the clarity of the model.

Specific points:

1) Fig 1e-g: Why would one expect a positive correlation (i.e. tug of war) between extension of axon and retraction of other neurites in the first place? This is not particularly intuitive.

In the classic winner-takes-all model, neurites are proposed to compete for a shared pool of limiting growth resources. In this framework, when one neurite captures these resources, it extends rapidly, while the remaining neurites are expected to retract. It is analogous to a tug-of-war, where the winning team pulls the rope (resources) toward them while the losing team slips the rope. Thus, a positive correlation between extension and retraction velocities is expected in such a model. To clarify this assumption, we have added explanatory text in the Discussion:

Line 361: "*A long-standing model has proposed that neurites of cortical neurons compete for limiting resources through their growth cones, with the "winning" neurite capturing growth-promoting cues while the others retract*[36]*. However, the oscillatory growth we observed is inconsistent with such a simple tug-of-war.*"

- At least in cultures, cells are growing in 2D, and for an axon to extend, a less dynamic change of other neurites would be expected to provide better overall traction/adherence and stability (do cell bodies remain in one position?).

In dissociated cultures, the soma remains stationary, and one might therefore expect that reducing dynamics in other neurites would improve overall traction or stability for axon extension. However, hippocampal neurons grow their axons independent of pulling forces. Specifically, previous studies from us and others showed that disrupting actin networks promotes neurite growth[7,10,40,41]. Moreover, our recent work revealed that, unlike moving fibroblasts, neurons with growing neurites do not exert pulling force on 3D collagen gels [42]. In addition, neurons in 3D gels inert to adhesion formation still grow axons [42]. Thus, neurites and axons grow in an adhesion-independent fashion. In other words, the classical view on the

growth cone that was considered a "fibroblast-on-a-leash" and with the thought that the neurite pulling strongest becomes the axon need to be rectified.

To make this clearer to readers, we emphasize in the Discussion:

Line 397: "*It is noteworthy that the clutch mechanism of mesenchymal cell migration*[43]*, which transduce myosin-driven retrograde actin flow to cell motion, appears not to apply to growth cones and neurite growth*[44,45]*. Indeed, growth cones of embryonic CNS neurons do not pull on 3-dimensional soft matrices to move forward*[42]*. Thus, the primary function of actomyosin networks in CNS neurons is not to generate traction force but to globally restrain neurite growth."*

2) P.5 text line 5. Comparison of polarization in response to chemotaxis is not the most apt comparison because for chemotaxis, there is a clear extracellular source of directional trigger, and moreover, cell polarization involves translocation of the cell body. The analogy invites confusion.

This text aimed to draw the link between oscillatory growth and actin. We removed "chemotaxis" and revised the text:

Line 93: "*As motile non-neuronal cells polarize by exiting from actin–driven oscillatory protrusions to directional movement*[46–49]*, we hypothesized that an actin-based system drives oscillatory growth.*"

To clarify this point further, we also added the basis for our hypothesis at the beginning of the result section:

Line 77: "*Oscillatory processes can orchestrate polarity in non-neuronal cells*[46–49]*. We therefore asked whether neurites exhibit oscillatory growth before neurons polarize.*"

3) Ext Fig 2: Kymographs show that actin spots appear and disappear all of a sudden, even for those at the leading edge, which makes it difficult to infer their translocation. Also the image seems pixellated. Would it be possible to show the kymograph (and/or image) at higher spatio-temporal resolution?

To address this, we re-acquired all relevant datasets with substantially higher temporal resolution – either 4-fold (1 frame/min) or 2-fold (1 frame/2 min) faster than in the original submission. We also did this for neurons before, during, and after polarization, both *in vitro* and in cortical slices. The updated figures (now **Fig. 2b, Fig. 2g, and Extended Data Fig. 2h**) allow the translocation of actin waves to be directly followed in the kymographs and further reinforce our conclusion that actin waves are tightly coupled to oscillatory neurite growth prior to polarization.

4) Fig 2e: How does the extent change in somatic F-actin intensity relate to the occurrence of F-actin waves? (i.e. What is monitoring the level of somatic F-actin patches and F-actin waves at the neurite?) Is monomeric actin pool shared between somatic F-actin patches and F-actin waves entering the neurite? Relating to the point above on kymographs, FRAP may show a clearer relationship between somatic F-actin patches and F-actin waves beyond correlations.

To clarify the relationship between somatic F-actin levels and wave initiation, we quantified soma actin intensity over time and compared the intensity change profile with actin wave emergence at the soma. We found that actin wave formation was associated with 5% reduction of the total soma actin. We added the result in **Fig. 2d** and in the text:

Line 111: "*In line with theoretical patch-to-wave transformation of actin*[29]*, 63± 12% (mean ± SD) of wave-forming events followed a 5 ± 4% (mean ± SD) decrease in total somatic actin patch intensity (**Fig. 2c-d; Extended Data Fig. 2a**). Actin waves formed with a frequency of 2.6 ± 0.7 waves per hour (mean ± SD; **Extended Data Fig. 2b**), matching the interval 25 ± 7.5 min between extension and retraction phases (mean ± SD; **Extended Data Fig. 1g**)."*

Our measurements showed that individual Arp3 patches are very short-lived (< 10 s; **Extended Data Fig. 3f**). Consistent with this, our new results showed that the photoconverted Arp3-mEos3.2 in the soma diffused away within 2 min (**Extended Data Fig. 3g-h)**. These results indicate that the entire somatic Arp3 pool—including many transient patches—rapidly exchanges with the surrounding cytoplasm. Consistently,

comparable actin turnover rates have been reported: photoconverted somatic actin in rat hippocampal DIV-1 neurons rapidly diffuses away from the soma within 2 minutes[51], and the full recovery time of the bleached cortical actin in melanoma M2 cells is 80 sec[52]. These actin dynamics all occur on time scales at least 10-times faster than wave formation (10–20 min). Therefore, actin and Arp2/3 continuously recycle between transient patches, the developing wave, and the monomeric actin pool during each oscillatory cycle.

The result is added in **Extended Data Fig. 3g-h**, with text:

Line 145: "*Somatic Arp3 patches were short-lived, with lifetimes of less than 10 s (**Extended Data Fig. 3f**). Consistently, photoconverted somatic Arp3-mEos3.2 rapidly diffused away within 2 min (**Extended Data Fig. 3g-h**), demonstrating rapid turnover of Arp3 in these patches.*"

5) Fig 2g-k: Photoactivation of PA-Rac1 may simply reflect a gain of function effect. Inhibition experiments might be more insightful.

We thank reviewer #2 for this helpful suggestion. To locally suppress Arp2/3, we performed an optogenetic experiment with the dominant-negative PA-Rac1 variant PA-Rac1-T17N[53]. We found that local suppression of Arp2/3 at the growth cone induces collapse of growth cone and neurite retraction. The result supports our conclusion that Arp2/3 at the neurite growth cone counteracts retraction. The result is included in **Fig. 3n** and **Extended Data Fig. 5c**, together with the controls and other experimental groups in the revised manuscript. We added the text:

Line 190: "*Conversely, activation of a dominant-negative PA-Rac1 mutant (T17N) collapsed the growth cone and triggered retraction **(Fig. 3n and Extended Data Fig. 5c; Supplementary Video 14).***"

In addition, we also established a local perfusion setup to apply Arp2/3 inhibitor CK-666 in a spatially restricted fashion. By combining an ejection capillary with an aspiration capillary, we created a perfusion field with maximal diameter of 80 µm for 90 minutes. We closely monitored the perfusion area with fluorescent dye FastGreen that was mixed with CK-666 solution and loaded in the ejection pipette.

We found that local perfusion of CK-666 at the soma induced spatially asymmetric effects. The short neurites that were covered by the perfusion retracted. Intriguingly, in more than half of the neurons (14/25 versus 1/19 in DMSO control), the neurites outside of the perfusion field generated retrograde actin waves that originated from the growth cone and moved retrogradely along the shaft toward the soma. The retrogradely moving actin wave disappeared upon entering the CK-666-perfusion area. The result suggests that wave initiation depends on local Arp2/3 activity, not on somatic location.

We added the results in **Fig. 3o-r** and **Extended Data Fig. 5i**, and text:

Line 208: "*Notably, wave initiation depended on local Arp2/3 activity and was not restricted to the soma: in more than half of the neurons, a neurite located outside the perfused area generated retrograde actin waves originating from its growth cone (14/25 versus 1/19 in DMSO control; **Fig. 3p-r**). These waves propagated toward the soma but dissipated upon reaching the CK-666-exposed region.*"

Moreover, our data showed that myosin II activity constrains neurite extension and drives retraction: inhibiting myosin II with *para*-aminoblebbistatin increased the extension induced by PA-Rac1 activation at the neurite tip, and abolished the retraction induced by PA-Rac1 activation at the soma. Consistently, local perfusion of *para*-aminoblebbistatin showed that the perfused neurites, but not the neurites outside of perfusion area, extended.

We added the results in **Extended Data Fig. 7a-f** and text

Line 240: "*Consistent with the idea that global myosin II activity restrains neurite extension and drives retraction following Arp2/3 activation, inhibiting myosin II with para-aminoblebbistatin enhanced the extension induced by PA-Rac1 activation at the neurite tip (**Extended Data Fig. 7a-b**). Moreover, para-aminoblebbistatin abolished the retraction triggered by PA-Rac1 activation at the soma (**Extended Data Fig. 7c-d**). Consistently, local perfusion of para-aminoblebbistatin at the soma and short neurites induced neurite extension (**Extended Data Fig. 7e-f**).*"

6) Having said this, what happens if PA-Rac1 is activated at multiple foci (i.e. more than two)? Would there be a competition for resources required for neurite elongation? If neurite formation is due to random activation of Arp2/3, then one might not expect to see competition.

We followed the advice and performed PA-Rac1 activation at three neurite tips/growth cones simultaneously. Quantification of actin intensity change showed that actin intensity increased in only one or maximally two of the tips/growth cones (**Extended Data Fig. 5d-e**). In contrast, actin intensity decreased in the third growth cones upon activation. The restricted number of activated growth cones aligns with our observation that only one neurite (or maximal two neurites) extends simultaneously during the transient polarization stage (**Extended Data Fig.1b-c**) and that activating PA-Rac1 at the soma induces only one actin wave (**Extended Data Fig. 5f)**.

While the results could reflect possible competition for resources among neurites or activation sites, inhibiting myosin II in Arp3 KO neurons with blebbistatin induced supernumerary axons. Thus, with suppressed myosin II activity, Arp2/3 itself is not the limiting factor for axon formation; it is the antagonism between Arp2/3 and actomyosin that enables axon initiation and constrains axon number.

We added the result in **Extended Data Fig. 5d-e**, **Supplementary Video 15** and text:

Line 193: "*The cellular response to PA-Rac1 activation is limited to one or maximal two sites: simultaneous activation at three growth cones produced increased actin assembly in only one or two, while the third showed reduced actin levels (**Extended Data Fig. 5d-e and Supplementary Video 15**).*"

7) In addition, the spatial effects of PA-Rac1 activation spots relative to the site of neurite extension is not clear, at least from the representative examples/videos shown. Would one expect to see the most proximal neurite to the PA-Rac1 activation site to extend?

To improve clarity, we have revised the figures and videos by enhancing the visibility of PA-Rac1 activation sites (larger activation dots, clearer labels) and adding arrowheads to indicate wave positions and trajectories. We also revised the Results text to ensure consistent descriptions of the effects (extension vs. retraction).

We would like to clarify that the outcome of PA-Rac1 activation, neurite extension vs. retraction, depends on the subcellular location of activation.

Soma activation:

In **Supplementary Video 17** and **Fig. 3m** (formerly Supplementary Video 4 and Fig. 2j), PA-Rac1 activation at the soma induced a lamellipodial protrusion that initiated a nascent actin wave. This was accompanied by retraction of adjacent neurites. We show that this retraction is myosin II-dependent, as it is abolished by *para*-aminoblebbistatin (new data in **Extended Data Fig. 7c-d**).

Growth cone activation:

In **Supplementary Video 13** and **Fig. 3k and n** (formerly Supplementary Video 4 and Fig. 2h-i), PA-Rac1 activation at the neurite tip induced growth cone expansion and neurite extension, an effect that was enhanced by *para*-aminoblebbistatin (**Extended Data Fig. 7a-b**). These new results are now highlighted in the text:

Line 240: "*Consistent with the idea that global myosin II activity restrains neurite extension and drives retraction following Arp2/3 activation, inhibiting myosin II with para-aminoblebbistatin enhanced the extension induced by PA-Rac1 activation at the neurite tip (**Extended Data Fig. 7a-b**). Moreover, para-aminoblebbistatin abolished the retraction induced by the activation of PA-Rac1 at the soma (**Extended Data Fig. 7c-d**).*"

Finally, the effects of PA-Rac1 activation are fully consistent with our observations in cortical slices and cultured neurons: somatic actin waves coincide with minor neurite retraction, whereas waves arriving at growth cones promote extension (**Fig. 1h; Fig. 2b, g-k; Extended Data Fig. 2h-k**). These relationships also agree with the cross-correlations of local actin fluctuations and oscillatory neurite growth (**Fig. 2e**).

7’) The reviewer raised the question in the beginning: How is oscillatory neurite growth coupled to localized Arp2/3 activation? This is related to the location-dependent effects of Arp2/3 on neurite growth and how actin branching and myosin II interact.

First, activating somatic Arp2/3 generates a branching-rich lamellipodium at the soma periphery as a nascent actin wave. As branching meshworks are enriched at the soma, they are simultaneously decreased in the neurites – as our cross-correlation analysis reveals (**Fig. 2e**). With drop of branching in the neurites, actomyosin networks contract and cause neurite retraction. In addition, because of the proximity of myosin II to Arp2/3 patches at the soma, the Arp2/3-nucleated actin filaments fuel actomyosin assembly before the antagonistic branched meshworks could form. Thus, the wave formation at the soma is associated with myosin II-driven neurite retraction. Consistently, we found that inhibiting myosin II with *para*-aminoblebbistatin suppressed the retraction caused by activating PA-Rac1 at the soma (**Extended Data Fig. 7c-d**).

Then the nascent wave moves away from the soma and enters one nearby neurite. The wave moves towards the tip of the entered neurite and increases local branching level, transforming the tip into an active growth cone. There, actin branching counteracts actomyosin constraint and enables microtubule-driven neurite growth. Consistently, inhibiting myosin II with *para*-aminoblebbistatin increased the extension caused by activating PA-Rac1 at the neurite tip (**Extended Data Fig. 7a-b**).

Without sustained Arp2/3 activity at the growth cone to keep the antagonism, the severing activity of myosin II[54–58] readily disrupts branch meshworks and converts them into linear filaments, which in turn fuel antiparallel actomyosin assembly. Consequently, myosin II regains dominance and resumes local actomyosin integrity. Meanwhile, the recurring somatic wave formation also decreases actin branching meshworks of neurites. Thereby, the neurite stops extension and starts retraction.

To clarify the relationship between the location of Arp2/3 activation and oscillatory neurite growth, we now added results of local Arp2/3 inactivation with the dominant negative PA-Rac1-T17N and local-perfusion of CK-666:

Line 190: “*Conversely, activation of a dominant-negative PA-Rac1 mutant (T17N) collapsed the growth cone and triggered retraction* ***(Fig. 3n, Extended Data Fig. 5c and Supplementary Video 14)****.*”

Line 205: “*Conversely, locally inactivating Arp2/3 by perfusing CK-666[7] induced spatially asymmetric effects (****Fig. 3o-q and Supplementary Video 18****). When CK-666 was perfused over the soma and short neurites, these neurites retracted, whereas neurites outside the perfused region or in vehicle treated controls did not (****Extended Data Fig. 5i****).*”

We also added a scheme to clarify the relationship between the location of Arp2/3 activation and the phase in oscillatory neurite growth in **Fig. 3j**, in addition to **Extended Data Fig. 5a** (original Fig. 2g**)**.

8) Fig 3a: In the LEGI model, it is not clear what is the trigger for a localized relaxation of global inhibition (i.e. local excitation) to allow the outgrowth of a single neurite. How is the preferred neurite specified and/or ends up being preferred?

In our activator-inhibitor framework, the trigger for localized “excitation” is intrinsic, arising from the spontaneous, periodic formation of somatic actin waves, not from external cues. Somatic Arp2/3 activation drives these waves and serves as the local activator, while the pre-existing actomyosin network functions as a global inhibitor that normally retracts minor neurites and constrains axon number. Because Arp2/3 has location-dependent effects, somatic wave initiation promotes actomyosin-driven retraction, whereas the arrival of the wave at a neurite tip promotes extension by counteracting actomyosin. Thus, when a wave emerges stochastically, the nearest neurite transiently escapes global inhibition and extends, while others remain suppressed, introducing a cell-intrinsic, iterative bias toward selecting one neurite.

However, this transient bias must be stabilized to specify the axon. Our data show that microtubule stabilization performs this consolidation step. Neurites undergoing retraction after Arp2/3 inhibition exhibit pronounced microtubule buckling, whereas emerging axons resist retraction and lack these buckles, consistent with stabilized microtubules. The hallmark of the future axon is therefore its prolonged, uninterrupted extension, which we observe only in neurites that “escape” the oscillatory cycle. Accordingly,

we propose that Arp2/3-mediated relief of actomyosin contractility enables microtubule stabilization, which in turn prevents retraction and locks in axon identity.

Together, these findings support a model in which the Arp2/3–actomyosin activator–inhibitor system provides the intrinsic symmetry-breaking trigger, and microtubule stabilization solidifies the privileged neurite into a true axon. This framework is consistent with prior reports showing that lowering actomyosin activity accelerates polarization by facilitating axon formation[39].

9) Fig 4: Related to the point above, how Arp2/3 contributes to the formation of no more than a single neurite extension is not clear here.

Our data indicate that Arp2/3 constrains the number of extending neurites through its dual, location-dependent roles within the activator–inhibitor system. As an Arp2/3-generated actin wave emerges at the soma and enters a single neurite, that neurite undergoes transient extension, becoming a “transient axon.” Two outcomes can then occur. (i) The transient axon may retract due to somatic actomyosin activity, returning the cell to a new cycle of oscillation. (ii) Alternatively, if microtubules in that neurite become sufficiently stabilized, the actomyosin network can no longer retract it, allowing it to escape the oscillatory regime and consolidate into a nascent axon. Once this occurs, the remaining minor neurites experience increased retraction rate (**Fig. 2k and Extended Data Fig. 2k**), and the cell no longer supports formation of additional axons.

The reason only one neurite can extend at a time is that the number of extending processes is constrained by global actomyosin-mediated inhibition. Our work suggests that the pre-existing somatic actomyosin network functions as this global constraint, retracting all neurites by default and thereby limiting axon number. We clarified Arp2/3’s role in this process through multiple perturbations:

Myosin II as the global growth constraint: Inhibiting myosin II with blebbistatin rescues axon elongation in Arp2/3-deficient neurons to WT-like levels (**Fig. 5i-k and Extended Data Fig. 12e-i**). This demonstrates that the principal function of Arp2/3 in axon formation is to relieve actomyosin-driven global inhibition; without myosin II, Arp2/3-dependent waves are not required for axon extension.

In summary, Arp2/3 contributes to the formation of a single axon by (1) reinforcing somatic actomyosin networks that suppress supernumerary neurite extension and (2) enabling one neurite to escape this global inhibition through local counteraction of actomyosin and cooperation with microtubule stabilization. This dual mechanism explains why only one neurite becomes the axon.

10) The analyses of the effects of loss of Arp2/3 presented here is limited to the characterization of axon/the longest neurite that has already formed.

To strengthen that Arp2/3 mediates the cell-intrinsic polarization and to extend our analysis of Arp3 KO growth dynamics under physiological condition, we did the following experiments:

1. Neuron-specific Arp3 KO via *in-utero* electroporation (IUE) of Cre recombinase in Arp3$^{fl/-}$ brains. We found that Arp3 KO neurons failed to form an axon. This clarifies that the axon deficiency of Arp3 KO neurons was caused by defective cell-intrinsic polarization.
   We added the result in **Fig.4g-h** and the text:
   Line 272: “*To test whether the defect reflects impaired cell-intrinsic polarization, we used tubulin α1 promotor to drive neuron-specific expression of Cre through IUE. As tubulin-α1 promotor starts to express later than the Nestin promoter*[15,16]*, we used Arp3*$^{fl/-}$ *brains to ensure efficient Arp2/3 depletion. The result consistently showed abrogated axon formation (**Fig. 4g-h**).”*
2. Live-cell imaging of Arp3 KO neurons in cortical slice: We expressed LynTM-FP in E15.5 brains via IUE and analyzed neurite growth dynamics of WT and Arp3 KO neurons. We found that Arp3 KO neurons in cortical slices had reduced growth dynamics as observed *in vitro*.
   We added the result in **Fig.4n-p** and the text:
   Line 288: “*In addition, both Arp3 KO cortical slices and cultured Arp3 KO neurons exhibited a reduction of growth dynamics (**Fig. 4n-p; Supplementary Videos 23**).*”

11) In addition, the data shown in extended Figures – the lack of effect of PA-Rac1 activation on F-actin dynamics and neurite length – are important in clarifying the role of Arp2/3 and should be presented as main data.

We agree with reviewer #2 that PA-Rac1 in Arp3 KO is an important control to demonstrate the specific activation of Arp2/3. We moved the neurite length quantification including all the controls (Arp3 KO and PA-^{C450M}-Rac1) and experimental groups to **Fig. 3n**. Furthermore, we added the quantification of actin intensity in the controls and the experimental groups in **Extended Data Fig. 5b-c**.

12) Ext Fig 10f,g: That CK-666 treatment reduces the length of minor neurites despite a lack of effect on the longest neurite in DIV-3 cultures is an important point and should be presented in the main figure. Could the authors show representative images of experiments in DIV-3 neurons?

Following this advice, we have added representative images and the experimental timeline for the CK-666 assay as **Fig. 3f,** and we have moved the original Extended Data Fig. 10f-g into the main figure panel (**now Fig. 3g-h**).

To further clarify how neuronal maturation influences axonal resistance to CK-666-induced retraction, we additionally performed time-lapse imaging of DIV-1, DIV-2, DIV-3, and DIV-4 polarized neurons treated with CK-666. We found that axonal resistance to retraction increases with maturation: only 37% of early DIV-1/DIV-2 neurons maintained an intact axon, whereas 70% of DIV-3/DIV-4 neurons preserved their axon despite Arp2/3 inhibition. These data strengthen the conclusion that maturing axons progressively acquire stability mechanisms that protect them from global contractile forces.

We have incorporated these findings into **Fig. 3f-i** and **Extended Data Fig. 4d-f** and added the following text in the revised manuscript:

Line 163: "*In polarized neurons, however, nascent axons, which no longer received actin waves, progressively became resistant to CK-666-induced retraction, with resistance rising from 37 ± 26% at DIV-1/2 to 70 ± 18% at DIV-3/4;* ***Fig. 3f-i; Extended Data Fig. 4d-f; Supplementary Video 10****). Interestingly, minor neurites of polarized neurons, which continued to receive waves, remained sensitive to CK-666 and retracted upon treatment (****Fig. 3h****).*"

13) Does photoactivating PA-Rac1 in DIV-3 neurons enable short neurites to elongate? Under such conditions, what happens to the longest neurite?

We attempted to do the experiment as suggested but found the neurons expressing PA-Rac1 for 3 days hardly polarized and mostly died or showed abnormal morphologies by DIV-3. We speculate that this was due to the basal level of PA-Rac1 activity even under dark state.

14) Fig 5d-g: It is not clear how the experiments here relate to the stability of axon (i.e. resistance to retraction) to Arp2/3 inhibition shown at DIV3. 48 h post-plating would imply that the cultures are DIV2, in which case, are axons already resistant to CK-666 treatment at 48 h post-plating? How about the behavior of axons to taxol treatment in DIV-3 neurons with or without CK-666? Terminology (DIV or post-plating hours) should be consistent throughout the manuscript.

We thank reviewer #2 for pointing out our confusing description. For the original Fig. 5d-g (48 h) and Extended Data Fig. 10 h-I (24 h), the time label indicates **the duration** of treatment, not the onset of treatment. In both 24 h and 24 h groups, the indicated compound (nocodazole or Taxol) was added right after the attachment of neurons to the dish. Thus, at the onset of treatment, the axon was not yet formed. After 24 or 48 hours of treatment, cells were fixed for immunostaining. For clarification here, the experimental design is depicted as timeline below:

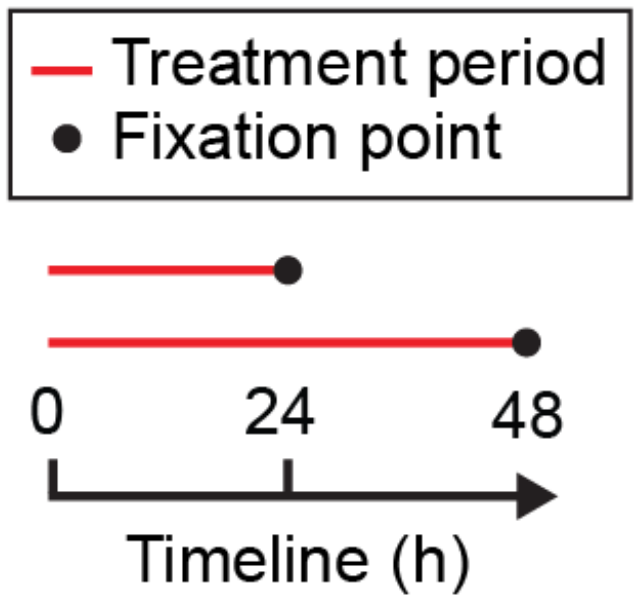

Given that the data of 24 h and 48 h essentially deliver overlapping information, we decided to keep only the 48 h results (now in **Fig. 5a-d**, with timeline). To avoid potential confusion in the experimental design of pharmacological treatments, we now added experimental timelines in the panels including **Fig. 3f** (DIV-1 vs DIV-3 WT neurons with CK-666) **Fig. 5i** (WT vs Arp3 KO with blebbistatin) **Extended Data Fig. 4g** (WT with CK-666 and washout), and **Extended Data Fig. 12c** (WT with blebbistatin and washout).

15) Also, does taxol treatment in DIV-3 neurons have any effect on the lengths of minor neurites? That is, given that taxol promotes the formation of multiple axons, if a single axon is already specified, then does the cell still have a capacity to turn the other minor neurites into axons?

Indeed, we had previously shown that taxol treatment in DIV-3 neurons transforms minor neurites into growing axons[59]. Hence, microtubule stabilization is still sufficient for axon formation at a later stage of development. This further supports our conclusion that microtubule stabilization is key for the neurite to become an axon. We have added this point in the text:

Line 335: "*Collectively, these results indicate that Arp2/3-mediated actin branching prevents compressive buckling of microtubules by counteracting contractile forces along neurites, thereby enabling sustained neurite extension (**Fig. 5h**).*"

16) Ext Fig 10l-n: Following the washout of CK-666, does the previously longest neurite again become the longest or do other neurites extend further?

We thank the reviewer for this question. To directly address it, we performed the suggested experiment in polarized neurons and tracked CK-666-induced retraction and washout-induced regrowth at 1 frame/min for 16 hours. Upon CK-666 treatment, 47% of polarized neurons retracted their axons and reverted to Stage 2 (no identifiable axon) or even Stage 1 (no neurite). Within this population, after washout, 58% regrew their original axon, 10% converted a different neurite into the new axon, and 32% failed to regenerate an axon. These results indicate that even after almost complete reorganization of actin networks and neurite retraction, neurons tend to re-establish their original polarity, rather than forming a new axon.

Further analysis of the washout videos revealed a consistent sequence of cytoskeletal events: during the first hour of CK-666 washout, we observed large somatic lamellipodia, accompanied by the reorganization of actin condensates into actin arcs at the rear of these actin protrusions. This somatic actin rearrangement correlated with transient retraction of remnant neurites, after which neurites regrew—typically along their original trajectories before CK-666 treatment. These observations further support our conclusion that somatic Arp2/3 activity promotes actomyosin-driven neurite retraction, and that relief of this constraint allows neurons to restore polarity.

We added the results in **Extended Data Fig. 4i**, **Supplementary Video 11 and 12**, and the associated text:

Line 170: "*Time-lapse imaging showed that, before regrowth, the actin condensates reorganized into lamellipodia and actin arcs at the soma periphery (**Extended Data Fig. 4i and Supplementary Video 11**). This reorganization was accompanied by a transient retraction of the remnant neurites followed by their regrowth in most cells **(81%; Extended Data Fig. 4i; Supplementary Video 11**). Interestingly, the original polarity was largely preserved upon*

*recovery (**Extended Data Fig. 4i and Supplementary Video 12**): among polarized neurons that had retracted their axons, 58% regrew the original axon, 10% formed a new axon, and 32% failed to regrow an axon.*"

17) Also, it would be informative to compare the behavior of EB3 in DIV1 vs DIV3 cultures where the longest neurite is resistant to CK-666 treatment. Does EB3 remain stable in DIV3 axons?

We performed analysis of the occurrence of EB3-labeled microtubule buckling in DIV-3 and DIV-4 polarized neurons and contrasted these results with DIV-1/DIV-2 cultures where axons are more sensitive to CK-666. We found a clear correlation between microtubule buckling and retraction behavior: 90% of axons that *did not* retract lacked EB3-labeled microtubule buckling, whereas 88% of retracted axons exhibited pronounced buckling. Moreover, the frequency of axonal microtubule buckling decreased with maturation, consistent with the increased resistance of DIV-3/DIV-4 axons to CK-666–induced retraction.

These results support our conclusion that Arp2/3 prevents neurite retraction by opposing actomyosin-driven compression of microtubule bundles, and that once microtubules become sufficiently stabilized during maturation, axons become retraction-resistant and Arp2/3-independent.

We now present these data in **Fig. 5e–g** with the following corresponding text

Line 328: "*In polarized neurons, these microtubule kinks were prevalent in minor neurites (**Fig. 5e-f**). Among polarized neurons undergoing axon retraction, 88% exhibited microtubule kinks in the retracting axon (**Extended Data Fig. 11g**). Consistent with the greater resistance of mature axons to retract (**Fig. 3g-i**), axonal microtubule kinks became less frequent with neuronal maturation (**Fig. 5g and Supplementary Video 28**).*"

18) In the model where local Arp2/3 activity breaks the symmetry of global inhibition and induces a single axon, focal activation of Arp2/3 is a key process. To this end, is it possible to deliver CK-666 locally and examine its effect, perhaps coupled to variously positioned local photoactivation of PA-Rac1?

Following the advice, we established local perfusion according to the setup described previously[7]. By combining an ejection capillary with an aspiration capillary, we created a perfusion field with maximal diameter of 80 µm for 90 minutes. We closely monitored the perfusion area with fluorescent dye FastGreen that was mixed with CK-666 solution and loaded in the ejection pipette.

We found that local perfusion of CK-666 at the soma induced spatially asymmetric effects. The short neurites that were covered by the perfusion retracted. In contrast, in more than half of the neurons (14/25 versus 1/19 in DMSO control), the neurites outside of the perfusion generated retrograde actin waves that originated from the tip and moved toward the soma. The retrogradely moving actin wave disappeared upon entering the CK-666-perfusion area. The result suggests that wave initiation depends on local Arp2/3 activity, not on somatic location.

We added the results in **Fig. 3o-r** and **Extended Data Fig. 5i**, and text:

Line 205: "*Conversely, locally inactivating Arp2/3 by perfusing CK-666[7] induced spatially asymmetric effects (**Fig. 3o-r and Supplementary Video 18**). When CK-666 was perfused over the soma and short neurites, these neurites retracted, whereas neurites outside the perfused region or in vehicle treated controls did not (**Extended Data Fig. 5i**). Notably, wave initiation depended on local Arp2/3 activity and was not restricted to the soma: in more than half of the neurons, a neurite located outside the perfused area generated retrograde actin waves originating from its growth cone (14/25 versus 1/19 in DMSO control; **Fig. 3p-r**). These waves propagated toward the soma but dissipated upon reaching the CK-666-exposed region.*"

Moreover, our data showed that myosin II activity constrains neurite extension and drives retraction: inhibiting myosin II with *para*-aminoblebbistatin increased the extension induced by PA-Rac1 activation at the neurite tip, but abolished the retraction induced by PA-Rac1 activation at the soma. Consistently, local perfusion of *para*-aminoblebbistatin showed that the perfused neurites, but not the neurites outside of the perfusion area, extended.

We added the results in **Extended Data Fig. 7a-f** and text

Line 240: "*Consistent with the idea that global myosin II activity restrains neurite extension and drives retraction following Arp2/3 activation, inhibiting myosin II with para-aminoblebbistatin enhanced the extension induced by PA-Rac1 activation at the neurite tip (**Extended Data Fig. 7a-b**). Moreover, para-aminoblebbistatin abolished the retraction triggered by PA-Rac1 activation at the soma (**Extended Data Fig. 7c-d**). Consistently, local perfusion of para-aminoblebbistatin at the soma and short neurites induced neurite extension (**Extended Data Fig. 7e-f**).*"

19) Fig 5k-m: Blebbistatin treatment is for 24 hours, and the slow timing of drug inhibition that is longer than the neurite outgrowth events complicates the data interpretation.

We are not sure of the "slow timing" referred to but believe that this comment came through our insufficient description of experimental design. Blebbistatin was added right after the attachment of neurons to the dishes, when neurons not yet grew any neurite. In addition, the original Supplementary Video 16 (now **Supplementary Video 30**) shows the effect of blebbistatin on neurite growth in WT and Arp3 KO neurons came rapidly within 4 hours of treatment. We added experimental timelines in the panels presenting pharmacological treatments, marking the onset of treatment, washout and fixation point. These panels include **Fig. 3f** (DIV-1 vs DIV-3 WT neurons with CK-666) **Fig. 5i** (WT vs Arp3 KO with blebbistatin) **Extended Data Fig. 4g** (WT with CK-666 and washout), **Fig. 5a** (WT vs Arp3 KO with Taxol or nocodazole), and **Extended Data Fig. 12c** (WT with blebbistatin and washout).

Reviewer #3:

In this paper by Lin et al., the authors re-examine the mechanisms of cell autonomous polarization of axons and dendrites that developing cortical neurons exhibit in vitro. It has been known for decades that cortical and hippocampal neurons will spontaneously polarize with a single long axon and multiple short dendrites over several well-defined stages after days in vitro. Previous work suggested that long-range signals originating from terminal growth cones determined which neurite became the axon. While in vivo there is likely an environmental cue that directs axons since they consistently extend toward the ventricle, how cortical neurons spontaneously polarize without external cues remains a mystery. This paper challenges current dogma by suggesting that axon outgrowth symmetry is broken by oscillatory waves of Arp2/3 driven actin polymerization originating in the cell body. This is observed as traveling waves of F-actin that extend up axons as described previously. Furthermore, the authors suggest that traveling branched actin networks relax acto-myosin contractility when they reach the terminal to promote axon extension.

While the experiments performed in this paper are elegant, cutting-edge, well-controlled and beautifully presented, it is not clear to me whether the conclusions reached are sufficiently novel for publication in Nature. It seems to me that a more specialized cell biology focused journal would be more appropriate.

1) One concern is the relevance of the mechanisms observed in vitro to how cortical neurons naturally polarize in vivo. As cortical neurons migrate along radial glial fibers and stereotypically extend their axons toward the ventricular zone during migration, it is not clear how directly comparable the processes observed on a homogenous PDL substratum in 2D cell culture. While the authors do show a similar correlation of extension and retraction in neurons in cortical slice culture, this one comparison does not prove the same mechanisms are involved (e.g are actin waves observed in vivo?).

We agree with the concern of this reviewer. To strengthen the physiological relevance of our work, we performed the following additional experiments:

1. **Live-cell imaging of unpolarized and polarized neurons in cortical slices:** We expressed Lifeact-FP in E15.5 brains via *in utero* electroporation (IUE) and analyzed the occurrence of actin waves and growth dynamics. Indeed, in unpolarized neurons, we found that waves formed at the soma with a frequency of 2-3 wave per hour; upon wave formation, adjacent neurites retracted. By contrast, axons did not acquire waves and grew in a wave-independent manner, recapitulating events we found also in cultured neurons.
   We added the result in **Fig.1h-l** and text:
   Line 99: "*Consistent with studies showing actin waves in cultured neurons*[1,2]*, unpolarized neurons in cortical slices repeatedly formed actin waves at the soma* ***(Fig. 1i)****. Strikingly, we found that upon wave formation, adjacent neurites rapidly retracted; the wave-receiving neurite then regrew* ***(Fig. 1h; Supplementary Video 3)****. In polarized neurons of cortical slices, by contrast, axons acquired no wave and elongated persistently, with extension times four-fold longer than retraction times* ***(Fig 1j-l; Extended Data Fig. 1g; Supplementary Video 4)***."
2. **Live-cell imaging of Arp3 KO neurons in cortical slices:** We expressed LynTM-FP in E15.5 brains via IUE and analyzed neurite growth dynamics of WT and Arp3 KO neurons. We found that Arp3 KO neurons in cortical slice had reduced growth dynamics as observed in vitro.
   We added the result in **Fig.4n-p** and the text:
   Line 288: "*both Arp3 KO cortical slices and cultured Arp3 KO neurons exhibited a reduction of growth dynamics* ***(Fig. 4n-p; Supplementary Videos 24 and 25)***."
3. **Arp3 KO in few neurons** via *in utero* electroporation of Cre recombinase in Arp3$^{fl/-}$ brain using a neuron specific tubulin-α1 promotor. We found that Arp3 KO neurons failed to form an axon. This clarifies that the axon deficiency of Arp3 KO neurons was caused by defective cell-intrinsic polarization.
   We added the result in **Fig. 4g-h** and the text:
   Line 272: "*To test whether the defect reflects impaired cell-intrinsic polarization, we used tubulin α1 promotor to drive neuron-specific expression of Cre through IUE. As tubulin-α1 promotor starts to express later than the Nestin promoter*[15,16]*, we used Arp3*$^{fl/-}$ *brains to ensure efficient Arp2/3 depletion. The result consistently showed abrogated axon formation (****Fig. 4g-h****).*"

Thus, this shows consistency between our *in vitro* and *in vivo* results further supporting our conclusion on the physiological relevance of our findings.

Of note, the study of *in vivo* neuronal polarization by Namba et al. (2014)[37] showed that the orientation of initial axon formation is independent of the direction of the guided growth *in vivo*. This suggests that, as with the polarization *in vitro*, neuronal polarization in the developing brain is dictated by a cell-intrinsic mechanism, not by the extrinsic input. Otherwise, the orientation of initial axon formation would follow extrinsic cues and align with the direction of the guided axon growth.

2) As environmental factors likely guide polarization in vivo, it seems likely that long-range signals initiated at axon terminals could contribute to polarization. Moreover, previous publications do suggest that paracrine signals released from terminals provide some local excitatory feedback signals that influence distant neurites (Takano et al., 2018). It is not clear how these findings fit with those described here.

We thank reviewer #3 for raising this important point. Indeed, several studies have proposed that growth cone-derived paracrine signals, including autocrine BDNF release, and long-range RhoA signaling ("Discovery of long-range inhibitory signaling to ensure single axon formation" by Takano *et al*.), may influence neurite selection *in vivo* and *in vitro*[60,61]. We agree that environmental cues can orient axon trajectory.

However, multiple lines of evidence indicate that these mechanisms are insufficient to account for neuronal polarization or for the restriction to a single axon:

1. Polarization is intact regardless of growing environment: Conceptually, if polarity were dictated solely by growth cone-derived signals, the pervasive neurotrophins in the developing brain should readily induce multiple axons, yet neurons consistently only form one. Even when two neurites contact growth-promoting substrates simultaneously, only one becomes the axon while the other is actively restrained[38].

2. Polarity is independent of axonal trajectory: The orientation of axon initiation is random and independent of the later trajectory guided by external cues[37], strongly suggesting that neuronal polarity *in vivo* is cell-intrinsically controlled, not pre-determined by the environmental cues that steer the growth cones.

3. Polarization is intact without ligand-receptor signalings: Neurons in knockout mice lacking extrinsic cues or their receptors BDNF/NT-3-TrkB, Sema3A-NP1, and others, polarize normally both *in vivo* and *in vitro*[62–66].

4. Polarization is intact with excessive neurotrophins: Elevating neurotrophin levels (via overexpression or bath application) in cultured neurons does not generate supernumerary axons[67,68].

5. Polarization is intact without RhoA signaling: RhoA had been thought to be a key regulator of the long-range inhibitory signaling. However, it was demonstrated that RhoA knockout does not impair neuronal polarization *in vitro* or *in vivo*[39]. Hence, it contradicts the idea that long-range inhibitory signaling between neurites coordinates axon number.

Our findings help reconcile these observations. We show that neurons undergo oscillatory cycles during early development, and the neurite that transiently escapes these cycles becomes the axon. Crucially, neurite growth is regulated not only by growth cones but also by the soma, where somatic Arp2/3-dependent waves originate and propagate into a single neurite. Furthermore, the global actomyosin network acts as a powerful, cell-intrinsic inhibitor that restricts axon number without requiring long-range extracellular signals.

Thus, while growth cones can interpret extrinsic cues to orient axon trajectory, the Arp2/3-actomyosin activator-inhibitor system provides the intrinsic symmetry-breaking mechanism that determines which neurite becomes the axon, even in the presence of abundant external guidance cues.

To clarify this conceptual point for readers, we added the following text to the Introduction:

Line 55: "*In this growth-cone-centered view, both neuronal polarity and axon formation would be dictated by the distribution of external guidance molecules, raising the possibility that multiple neurites could adopt axon identity under uniform exposure. However, this model is difficult to reconcile with the fact that neurons reliably form only a single axon in the developing brain, an environment rich in growth-promoting factors, and the initial site of axon*

emergence is not dictated by the eventual direction of guided axon extension[37]*. Even when two neurites of a neuron simultaneously contact growth promoting substrates, only one becomes the axon, and the other is restrained*[38]*.*"

3) This in vitro model system has been used for decades to understand neuronal polarization and it does provide useful cell biological information about the dynamic regulation of the cytoskeleton free from confounding variables. In this regard, the most significant conclusion reached by these authors in my opinion is the novel notion that branched networks of actin filaments somehow relax actomyosin contractility. While this is an interesting idea, the authors provide no mechanistic or biophysical explanation how this occurs. This conclusion is reached largely from observations of F-actin, Myo-II and MRLC distributions and correlation with actin waves and axon extension, together with specific manipulations of Arp2/3 and myosin activity.

We thank the reviewer for highlighting that our conclusion that Arp2/3-generated branched actin networks transiently relaxing actomyosin contractility is novel. We now provide a more explicit mechanistic and biophysical explanation grounded in prior literature. Two well-established mechanisms account for the reciprocal antagonism between Arp2/3 and myosin II:

1. Competition for actin filament binding sites. Arp2/3 and myosin II both engage actin filaments, but with different structural preferences. Because actin binding sites are limiting, increased Arp2/3 occupancy reduces myosin II access and thus decreases contractility[21].

2. Geometric incompatibility between branched networks (70 degrees) and anti-parallel bundles (180 degrees). Arp2/3 creates a dendritic network with fixed branch angles, which is structurally incompatible with myosin II-driven anti-parallel filament sliding[25–28]. The transient increase in branching disrupts filament alignment and thereby reduces contractility.

At longer timescales, however, Arp2/3 does not simply oppose myosin II. Multiple studies show that myosin II actively severs actin filaments[54–58]. This enables myosin II to convert branched networks into linear filaments that favor actomyosin bundling[54–58]. Consistent with this notion, Arp2/3 has been shown to facilitate myosin II recruitment in epithelial zonula adherens and a human osteosarcoma cell line U2O2 cells[69,70]. Thus, Arp2/3 can transiently soften the actomyosin network but later feed into its reinforcement.

Our observations fit well within this biophysical framework: We find that neuritic Arp2/3 activation transiently relaxes actomyosin, enabling neurite extension. Without sustained local Arp2/3 activity, the dominant myosin II eventually eliminates the branches and reforms contractile actomyosin bundles that retract neurites. In contrast, at the soma, because of the proximity of myosin II to Arp2/3 patches, the Arp2/3-nucleated actin filaments fuel actomyosin assembly before the antagonistic branched meshworks could form.

We now cite the papers founding the antagonistic mechanism and highlight the location-dependent organization of Arp2/3 and myosin II in the Discussion:

Line 366: "*Biochemical studies and modeling have suggested that Arp2/3-generated branched actin networks antagonize anti-parallel actomyosin bundles*[25–28,71–73]*.*"

Line 373: "*Interestingly, we found that activating Arp2/3 at the soma specifically induced transient retraction before growth resumed. This likely reflects the proximity of myosin II to Arp2/3 patches at the soma, unlike at growth cones, where myosin II is largely absent from the peripheral domain where Arp2/3 operates.*"

4) To reach this conclusion I believe more direct biophysical or molecular measurements would need to be made. For example, if Arp2/3 mediated F-actin waves suppress myosin activity, would one expect to see reduced p-MRLC (active) within traveling branched networks? Better yet, if a live biosensor for myosin-II activity could be used to correlate dynamic distributions with different actin filament networks, this would provide more direct comparisons.

As an additional support that Arp2/3 depletion upregulates myosin II, we monitored total myosin regulatory light chain (MRLC) and its phosphorylated (active) form. Analysis of biochemical neuronal extracts by western blotting showed that Arp3 KO neurons had level of phospho-MRLC more than double of the level in WT neurons. This is consistent with the idea that the depletion of actin branching increases the dominance of myosin II on the actin networks.

We added the result in **Extended Data Fig. 9h-i** and the text:

Line 297: "*Consistently, analysis of biochemical extracts of Arp3 KO neurons showed an increase of phospho-MRLC (**Extended Data Fig. 9h-i**), reporting higher myosin II activity*[74]."

5) The authors did measure retrograde flow, which is an indirect read-out of myosin II activity. However, they did not compare flow rates of retracting versus extending neurites, which should correlate with Arp2/3 content and branched F-actin.

We thank the reviewer for this thoughtful comment. We performed nested time-lapse imaging of neurite growth and actin dynamics at the growth cones. Kymograph analysis in combination with the determination of the growth state (extending or retracting) showed indeed that the extending neurites were associated with higher actin retrograde flow at the growth cones, while the retracting neurites were associated with lower actin retrograde flow.

We added the result in **Extended Data Fig. 3p-q** and the text:

Line 152: "*Consistent with previous work*[3,5,6]*, extending neurites ……, and exhibited faster actin retrograde flow at growth cones than during retraction* ***(Extended Data Fig. 3p-q)***."

6) However, these approaches still would not provide a mechanistic understanding how branched actin networks influence myosin-II activity. Reconstitution of branched actin and myosin II-driven F-actin sliding in a cell-free system may be necessary to conclusively demonstrate that this type of mechanism is involved.

We agree that fully reconstituting branched actin networks and myosin II-driven filament sliding in a cell-free system would be the most direct way to demonstrate the underlying mechanism. While such experiments are beyond the scope of this study, we note that multiple prior reconstituted and computational systems have already addressed exactly this question and converge strongly on the conclusion that Arp2/3-mediated branching antagonizes actomyosin contractility. Specifically:

1. Actin branching slows down contraction: Reymann et al. (2012)[25], Letort et al. (2015)[27] and Ennomani et al. (2016)[26] showed with reconstituted experiments and modeling that the architecture of actin networks determines myosin activity and governs the contraction of network. Increasing the proportion of branched meshworks slowed down the actomyosin contraction rates.

2. Actin branching impedes myosin processivity: Similarly, Muresan et al. (2022)[71] showed with reconstituted experiments that actin branching impedes contraction of skeletal actomyosin networks and prevents accumulation of mechanical tension by inhibiting myosin processivity.

3. Actin branching reduces mechanical stress: Consistently, Liman et al. (2020)[28] showed with modeling that branched actomyosin networks release the mechanical strain slower than unbranched networks.

4. Actin branching dissipates energy: Sakamoto et al. (2024)[73] showed that actin branching promotes futile ATP consumption and diverts energy away from productive myosin II-mediated contraction.

5. Actin branching competes with bundling factors: Graham et al. (2024)[72] used reconstituted condensates of actin networks and showed that by creating a mechanical barrier hindering bundling, Arp2/3 branching inhibited VASP-mediated filament bundling.

Together, these studies show that Arp2/3-generated dendritic networks are structurally incompatible with the contractile anti-parallel architecture of actomyosin networks and provide a strong mechanistic foundation for our observations. Such antagonism also aligns with the phenotypes of diverse cell types losing Arp2/3 activity, where the blebbing and large actin arcs recapitulate upregulation of actomyosin networks[75–83].

To clarify this rationale, we added the following to the revised manuscript:

Line 291: "*We hypothesized that these growth defects arise because, without Arp2/3, contractile actomyosin networks take over the cell. If Arp2/3-driven branching counteracts actomyosin networks*[24,25,71]*, then loss of Arp2/3 should increase myosin II activity*."

Line 366: "*Biochemical studies and modeling have suggested that Arp2/3-generated branched actin networks antagonize anti-parallel actomyosin bundles*[25–28,71–73]*. In line with this concept, both pharmacological inhibition of Arp2/3 and genetic loss of Arp3 produced a striking dominance of contractile actomyosin networks that prevented axon growth. Thus, Arp2/3 and actomyosin operate as opposing cytoskeletal systems whose balance governs neurite dynamics.*"

We would like to finish our letter by thanking the reviewers for their thoughtful and insightful comments that helped us to improve the manuscript.

References

1. Flynn, K. C., Pak, C. W., Shaw, A. E., Bradke, F. & Bamburg, J. R. Growth cone-like waves transport actin and promote axonogenesis and neurite branching. *Dev. Neurobiol.* **69**, 761–779 (2009).

2. Winans, A. M., Collins, S. R. & Meyer, T. Waves of actin and microtubule polymerization drive microtubule-based transport and neurite growth before single axon formation. *eLife* **5**, e12387 (2016).

3. Twelvetrees, A. E. *et al.* The dynamic localization of cytoplasmic dynein in neurons is driven by kinesin-1. *Neuron* **90**, 1000–1015 (2016).

4. Schelski, M. & Bradke, F. Microtubule retrograde flow retains neuronal polarization in a fluctuating state. *Sci. Adv.* **8**, eabo2336 (2022).

5. Jacobson, C., Schnapp, B. & Banker, G. A. A change in the selective translocation of the Kinesin-1 motor domain marks the initial specification of the axon. *Neuron* **49**, 797–804 (2006).

6. Yang, Q., Zhang, X.-F., Pollard, T. D. & Forscher, P. Arp2/3 complex-dependent actin networks constrain myosin II function in driving retrograde actin flow. *J. Cell Biol.* **197**, 939–956 (2012).

7. Bradke, F. & Dotti, C. G. The role of local actin instability in axon formation. *Science* **283**, 1931–1934 (1999).

8. Strasser, G. A., Rahim, N. A., VanderWaal, K. E., Gertler, F. B. & Lanier, L. M. Arp2/3 is a negative regulator of growth cone translocation. *Neuron* **43**, 81–94 (2004).

9. Ikeno, T. & Konishi, Y. Arp2/3 is required for axonal arbor terminal retraction in cerebellar granule neurons. *Neurochem. J.* **14**, 32–36 (2020).

10. Kunda, P., Paglini, G., Quiroga, S., Kosik, K. & Caceres, A. Evidence for the involvement of Tiam1 in axon formation. *J. Neurosci.* **21**, 2361–2372 (2001).

11. Tahirovic, S. *et al.* Rac1 regulates neuronal polarization through the WAVE complex. *J. Neurosci.* **30**, 6930–6943 (2010).

12. Montenegro-Venegas, C. *et al.* MAP1B regulates axonal development by modulating Rho-GTPase Rac1 activity. *Mol. Biol. Cell* **21**, 3518–3528 (2010).

13. San Miguel-Ruiz, J. E. & Letourneau, P. C. The role of Arp2/3 in growth cone actin dynamics and guidance is substrate dependent. *J. Neurosci.* **34**, 5895–5908 (2014).

14. Vinopal, S. *et al.* Centrosomal microtubule nucleation regulates radial migration of projection neurons independently of polarization in the developing brain. *Neuron* **111**, 1241-1263.e16 (2023).

15. Gloster, A. *et al.* The T alpha 1 alpha-tubulin promoter specifies gene expression as a function of neuronal growth and regeneration in transgenic mice. *J. Neurosci.* **14**, 7319–7330 (1994).

16. Dubois, N. C., Hofmann, D., Kaloulis, K., Bishop, J. M. & Trumpp, A. Nestin-Cre transgenic mouse line Nes-Cre1 mediates highly efficient Cre/loxP mediated recombination in the nervous system, kidney, and somite-derived tissues. *Genesis* **44**, 355–360 (2006).

17. Namba, T. *et al.* Extracellular and intracellular signaling for neuronal polarity. *Physiol. Rev.* **95**, 995–1024 (2015).

18. Schelski, M. & Bradke, F. Neuronal polarization: From spatiotemporal signaling to cytoskeletal dynamics. *Mol. Cell. Neurosci.* **84**, 11–28 (2017).

19. Kollins, K. M., Hu, J., Bridgman, P. C., Huang, Y. Q. & Gallo, G. Myosin-II negatively regulates minor process extension and the temporal development of neuronal polarity. *Dev. Neurobiol.* **69**, 279–298 (2009).

20. Takano, T. *et al.* Discovery of long-range inhibitory signaling to ensure single axon formation. *Nat. Commun.* **8**, 33 (2017).

21. Hetrick, B., Han, M. S., Helgeson, L. A. & Nolen, B. J. Small molecules CK-666 and CK-869 inhibit actin-related protein 2/3 complex by blocking an activating conformational change. *Chem. Biol.* **20**, 701–712 (2013).

22. Cao, L., Huang, S., Basant, A., Mladenov, M. & Way, M. CK-666 and CK-869 differentially inhibit Arp2/3 iso-complexes. *EMBO Rep.* **25**, 3221–3239 (2024).

23. Witte, H., Neukirchen, D. & Bradke, F. Microtubule stabilization specifies initial neuronal polarization. *J. Cell Biol.* **180**, 619–632 (2008).

24. Lomakin, A. J. *et al.* Competition for actin between two distinct F-actin networks defines a bistable switch for cell polarization. *Nat. Cell Biol.* **17**, 1435–1445 (2015).

25. Reymann, A.-C. *et al.* Actin network architecture can determine myosin motor activity. *Science* **336**, 1310–1314 (2012).

26. Ennomani, H. *et al.* Architecture and connectivity govern actin network contractility. *Curr. Biol.* **26**, 616–626 (2016).

27. Letort, G. *et al.* Geometrical and mechanical properties control actin filament organization. *PLoS Comput. Biol.* **11**, e1004245 (2015).

28. Liman, J. *et al.* The role of the Arp2/3 complex in shaping the dynamics and structures of branched actomyosin networks. *Proc Natl Acad Sci USA* **117**, 10825–10831 (2020).

29. Suarez, C. *et al.* Profilin regulates F-actin network homeostasis by favoring formin over Arp2/3 complex. *Dev. Cell* **32**, 43–53 (2015).

30. Burke, T. A. *et al.* Homeostatic actin cytoskeleton networks are regulated by assembly factor competition for monomers. *Curr. Biol.* **24**, 579–585 (2014).

31. Bradke, F. & Dotti, C. G. Establishment of neuronal polarity: lessons from cultured hippocampal neurons. *Curr. Opin. Neurobiol.* **10**, 574–581 (2000).

32. Takano, T., Funahashi, Y. & Kaibuchi, K. Neuronal polarity: positive and negative feedback signals. *Front. Cell Dev. Biol.* **7**, 69 (2019).

33. Zou, Y. Breaking symmetry - cell polarity signaling pathways in growth cone guidance and synapse formation. *Curr. Opin. Neurobiol.* **63**, 77–86 (2020).

34. Arimura, N. & Kaibuchi, K. Neuronal polarity: from extracellular signals to intracellular mechanisms. *Nat. Rev. Neurosci.* **8**, 194–205 (2007).

35. da Silva, J. S. & Dotti, C. G. Breaking the neuronal sphere: regulation of the actin cytoskeleton in neuritogenesis. *Nat. Rev. Neurosci.* **3**, 694–704 (2002).

36. Inagaki, N., Toriyama, M. & Sakumura, Y. Systems biology of symmetry breaking during neuronal polarity formation. *Dev. Neurobiol.* **71**, 584–593 (2011).

37. Namba, T. *et al.* Pioneering axons regulate neuronal polarization in the developing cerebral cortex. *Neuron* **81**, 814–829 (2014).

38. Esch, T., Lemmon, V. & Banker, G. Local presentation of substrate molecules directs axon specification by cultured hippocampal neurons. *J. Neurosci.* **19**, 6417–6426 (1999).

39. Dupraz, S. *et al.* Rhoa controls axon extension independent of specification in the developing brain. *Curr. Biol.* **29**, 3874-3886.e9 (2019).

40. Bradke, F. & Dotti, C. G. Differentiated neurons retain the capacity to generate axons from dendrites. *Curr. Biol.* **10**, 1467–1470 (2000).

41. Chia, J. X., Efimova, N. & Svitkina, T. M. Neurite outgrowth is driven by actin polymerization even in the presence of actin polymerization inhibitors. *Mol. Biol. Cell* **27**, 3695–3704 (2016).

42. Santos, T. E. *et al.* Axon growth of CNS neurons in three dimensions is amoeboid and independent of adhesions. *Cell Rep.* **32**, 107907 (2020).

43. Elosegui-Artola, A. *et al.* Mechanical regulation of a molecular clutch defines force transmission and transduction in response to matrix rigidity. *Nat. Cell Biol.* **18**, 540–548 (2016).

44. Wylie, S. R., Wu, P. J., Patel, H. & Chantler, P. D. A conventional myosin motor drives neurite outgrowth. *Proc Natl Acad Sci USA* **95**, 12967–12972 (1998).

45. Kerstein, P. C., Nichol, R. H. & Gomez, T. M. Mechanochemical regulation of growth cone motility. *Front. Cell. Neurosci.* **9**, 244 (2015).

46. Graziano, B. R. & Weiner, O. D. Self-organization of protrusions and polarity during eukaryotic chemotaxis. *Curr. Opin. Cell Biol.* **30**, 60–67 (2014).

47. Shi, C., Huang, C.-H., Devreotes, P. N. & Iglesias, P. A. Interaction of motility, directional sensing, and polarity modules recreates the behaviors of chemotaxing cells. *PLoS Comput. Biol.* **9**, e1003122 (2013).

48. Giannone, G. *et al.* Periodic lamellipodial contractions correlate with rearward actin waves. *Cell* **116**, 431–443 (2004).

49. Ponti, A. *et al.* Periodic patterns of actin turnover in lamellipodia and lamellae of migrating epithelial cells analyzed by quantitative Fluorescent Speckle Microscopy. *Biophys. J.* **89**, 3456–3469 (2005).

50. Carlsson, A. E. Dendritic actin filament nucleation causes traveling waves and patches. *Phys. Rev. Lett.* **104**, 228102 (2010).

51. Meka, D. P. *et al.* Radial somatic F-actin organization affects growth cone dynamics during early neuronal development. *EMBO Rep.* **20**, e47743 (2019).

52. Fritzsche, M., Lewalle, A., Duke, T., Kruse, K. & Charras, G. Analysis of turnover dynamics of the submembranous actin cortex. *Mol. Biol. Cell* **24**, 757–767 (2013).

53. Wu, Y. I. *et al.* A genetically encoded photoactivatable Rac controls the motility of living cells. *Nature* **461**, 104–108 (2009).

54. Haviv, L., Gillo, D., Backouche, F. & Bernheim-Groswasser, A. A cytoskeletal demolition worker: myosin II acts as an actin depolymerization agent. *J. Mol. Biol.* **375**, 325–330 (2008).

55. Ishikawa, R., Sakamoto, T., Ando, T., Higashi-Fujime, S. & Kohama, K. Polarized actin bundles formed by human fascin-1: their sliding and disassembly on myosin II and myosin V in vitro. *J. Neurochem.* **87**, 676–685 (2003).

56. Medeiros, N. A., Burnette, D. T. & Forscher, P. Myosin II functions in actin-bundle turnover in neuronal growth cones. *Nat. Cell Biol.* **8**, 215–226 (2006).

57. Murrell, M. P. & Gardel, M. L. F-actin buckling coordinates contractility and severing in a biomimetic actomyosin cortex. *Proc Natl Acad Sci USA* **109**, 20820–20825 (2012).

58. Wilson, C. A. *et al.* Myosin II contributes to cell-scale actin network treadmilling through network disassembly. *Nature* **465**, 373–377 (2010).

59. Gomis-Rüth, S., Wierenga, C. J. & Bradke, F. Plasticity of polarization: changing dendrites into axons in neurons integrated in neuronal circuits. *Curr. Biol.* **18**, 992–1000 (2008).

60. Cheng, P.-L. *et al.* Self-amplifying autocrine actions of BDNF in axon development. *Proc Natl Acad Sci USA* **108**, 18430–18435 (2011).

61. Nakamuta, S. *et al.* Local application of neurotrophins specifies axons through inositol 1,4,5-trisphosphate, calcium, and Ca2+/calmodulin-dependent protein kinases. *Sci. Signal.* **4**, ra76 (2011).

62. Martínez, A. *et al.* TrkB and TrkC signaling are required for maturation and synaptogenesis of hippocampal connections. *J. Neurosci.* **18**, 7336–7350 (1998).

63. Polleux, F., Giger, R. J., Ginty, D. D., Kolodkin, A. L. & Ghosh, A. Patterning of cortical efferent projections by semaphorin-neuropilin interactions. *Science* **282**, 1904–1906 (1998).

64. Chen, G. *et al.* Semaphorin-3A guides radial migration of cortical neurons during development. *Nat. Neurosci.* **11**, 36–44 (2008).

65. Ma, L. *et al.* Neurotrophin-3 is required for appropriate establishment of thalamocortical connections. *Neuron* **36**, 623–634 (2002).

66. Takizawa, S. *et al.* CRMP2 and its phosphorylation prevent axonal misrouting of the corticospinal tract. *Neurosci. Lett.* **855**, 138231 (2025).

67. Wang, L. *et al.* Autocrine action of BDNF on dendrite development of adult-born hippocampal neurons. *J. Neurosci.* **35**, 8384–8393 (2015).

68. Labelle, C. & Leclerc, N. Exogenous BDNF, NT-3 and NT-4 differentially regulate neurite outgrowth in cultured hippocampal neurons. *Brain Res. Dev. Brain Res.* **123**, 1–11 (2000).

69. Verma, S. *et al.* A WAVE2-Arp2/3 actin nucleator apparatus supports junctional tension at the epithelial zonula adherens. *Mol. Biol. Cell* **23**, 4601–4610 (2012).

70. Tojkander, S. *et al.* A molecular pathway for myosin II recruitment to stress fibers. *Curr. Biol.* **21**, 539–550 (2011).

71. Muresan, C. G. *et al.* F-actin architecture determines constraints on myosin thick filament motion. *Nat. Commun.* **13**, 7008 (2022).

72. Graham, K. *et al.* Liquid-like condensates mediate competition between actin branching and bundling. *Proc Natl Acad Sci USA* **121**, e2309152121 (2024).

73. Sakamoto, R. & Murrell, M. P. F-actin architecture determines the conversion of chemical energy into mechanical work. *Nat. Commun.* **15**, 3444 (2024).

74. Watanabe, T., Hosoya, H. & Yonemura, S. Regulation of myosin II dynamics by phosphorylation and dephosphorylation of its light chain in epithelial cells. *Mol. Biol. Cell* **18**, 605–616 (2007).

75. Bergert, M., Chandradoss, S. D., Desai, R. A. & Paluch, E. Cell mechanics control rapid transitions between blebs and lamellipodia during migration. *Proc Natl Acad Sci USA* **109**, 14434–14439 (2012).

76. Chikina, A. S., Svitkina, T. M. & Alexandrova, A. Y. Time-resolved ultrastructure of the cortical actin cytoskeleton in dynamic membrane blebs. *J. Cell Biol.* **218**, 445–454 (2019).

77. Dey, S. K. *et al.* Differential role of nonmuscle myosin II isoforms during blebbing of MCF-7 cells. *Mol. Biol. Cell* **28**, 1034–1042 (2017).

78. Mills, J. C., Stone, N. L., Erhardt, J. & Pittman, R. N. Apoptotic membrane blebbing is regulated by myosin light chain phosphorylation. *J. Cell Biol.* **140**, 627–636 (1998).

79. Rotty, J. D. *et al.* Arp2/3 complex is required for macrophage integrin functions but is dispensable for fcr phagocytosis and in vivo motility. *Dev. Cell* **42**, 498-513.e6 (2017).

80. Langridge, P. D. & Kay, R. R. Blebbing of Dictyostelium cells in response to chemoattractant. *Exp. Cell Res.* **312**, 2009–2017 (2006).

81. Suraneni, P. *et al.* A mechanism of leading-edge protrusion in the absence of Arp2/3 complex. *Mol. Biol. Cell* **26**, 901–912 (2015).

82. Rotty, J. D. *et al.* Profilin-1 serves as a gatekeeper for actin assembly by Arp2/3-dependent and -independent pathways. *Dev. Cell* **32**, 54–67 (2015).

83. Suraneni, P. *et al.* The Arp2/3 complex is required for lamellipodia extension and directional fibroblast cell migration. *J. Cell Biol.* **197**, 239–251 (2012).

Preliminary response to the reviewer #3's comments on Nature 2023-11-21494A-Z:

"An intrinsic cytoskeletal oscillator establishes neuronal polarity"

We thank reviewer #3 for this additional round of constructive critique. In the following, we address these comments point-by-point.

The reviewer's comments are in blue font, our responses are in black font, and new text added to the manuscript is in black italic font.

Reviewer #3

I have re-reviewed the paper by Lin et al., and I am afraid my opinion has not changed. This is a very nice cell biological study that describes details of neuron polarization processes. The most impactful conclusion according to authors description is that a "soma-initiated oscillatory program primes axon selection", which contrasts with the growth cone centric held view previously regarding polarization. This conclusion is based on extensive analysis of live cell behaviors together with pharmacological and molecular manipulations. While this study is extremely detailed, its overall impact, combined with several minor concerns, reduces my enthusiasm for publication in Nature. I will list a few concerns below.

We appreciate the reviewer's recognition of our study and for pointing out "several minor concerns", which has helped us further improve the manuscript as described point-by-point below. While this reviewer considers our work as "a very nice cell biological study", we want to use this opportunity to clarify the overall impact of our work.

Neuronal polarization is not a niche phenomenon; it is the structural basis of directional information flow in the brain. It underlies how the neuron establishes a single axon, a long-standing and fundamental question in cell biology, development, and neurobiology.

For decades, this process has been largely interpreted through a growth cone-centric framework. As reviewer #3 acknowledged, we overturn this view and identify a soma-initiated oscillatory mechanism, which redefines how we understand this principled feature of circuit architecture.

The impact of our study is further strengthened by uncovering a cytoskeletal activator-inhibitor mechanism that translates these oscillations into neuronal polarization. This conceptual advance brings a broader principle to developmental and cell biology — spontaneous symmetry breaking through an oscillatory cytoskeletal mechanism. This framework provides a mechanistic explanation for how neurons reliably form a single axon even in the presence of abundant growth-promoting signals. The discovered symmetry-breaking mechanism underpins the key concept that neuronal polarization is cell-intrinsic rather than external cue-dependent. Together, these findings establish a generalizable principle with implications across neuroscience, cell biology, and developmental biology.

1) It is stated on Line 101 "Strikingly, we found that upon wave formation, adjacent neurites rapidly retracted". I do not see any change of adjacent neurites after wave enters primary neurite in figure or movie.

   We will revise the presentation to make the changes in the minor neurites more identifiable. Specifically, we will add asterisks in **Fig. 1h** and **Supplementary Video 3** to mark the beginning tip position as the reference to better contrast the ending tip position after retraction.

2) Moreover, the "waves" in cell bodies appear subtle (some appear to be blebs without increased lifeact intensity). It is not clear how these are subjectively scored as waves.

   The definition of actin waves through the manuscript and in other studies is growth-cone-like F-actin structures that translocate toward and along a neurite[1,2]. In the live-cell imaging of neurons expressing Lifeact, the actin wave and its translocation can be reliably identified based on coordinated changes in

local morphology and F-actin intensity (e.g. **Fig. 2a, 2g** and **Extended Data Fig. 2h**; **Supplementary Videos 5, 6** and **8**).

A technical note: In live-cell imaging of cortical slices, where the neurons are embedded in 3D environment, the nascent actin waves at the soma are partially captured as thin veils with lower Lifeact intensity. In contrast, in the 2D cultured neurons, actin waves cannot go beyond the glass bottom and thus the full wave structure is detected with high Lifeact intensity. Thereby, waves show higher actin intensities in 2D than in 3D environments. Accordingly, in slice preparations, we rely on both morphological dynamics and F-actin signal to identify actin waves.

We can rule out that the waves at the soma shown in **Fig. 1h** and **Supplementary Video 3** are blebbing events for the following reasons: First, cellular blebs can expand and retract but, to our knowledge, do not exhibit directional translocation along neurites as actin waves do[3,4]. Second, we showed that in Arp3 KO neurons, actin waves were abolished (**Extended Data Fig. 9c; Supplementary Video 24**) and actomyosin was upregulated (**Fig. 4l-m; Extended Data Fig. 9h**). Consistently, inhibiting myosin II with blebbistatin increased the occurrence of waves (**Extended Data Fig. 12j**)[1]. In contrast, cellular blebs are induced by upregulated actomyosin but suppressed by blebbistatin[5,6]. And third, blebbing is reported to be independent of Arp2/3 activity[7,8], further distinguishing it from the actin waves described here.

3) Moreover and most importantly, often waves appear to form first in proximal processes near the cell body (Fig 2a). Are these also considered cell soma waves?

We agree that the actin wave formation in **Fig. 2a** appears at the soma–proximal neurite interface rather than strictly within the soma. Indeed, actin waves can also emerge at or near this interface, which is consistent with our model. We established the temporal and quantitative relationship between somatic actin patch intensities and wave emergence as shown in **Fig. 2c, 2d** and **Extended Data Fig. 2a**: actin patch intensity builds up at the soma until a wave is triggered, after which patch intensity drops and gradually rebuilds. Importantly, this temporal sequence indicates that the initiating event is somatic, even when the visible wavefront appears at the proximal neurite base.

In addition, to address the central debate in neuronal polarization, the mechanistically relevant comparison is between the perisomatic region and the distal growth cones; finer sub-regional distinctions (e.g., soma versus proximal base) do not alter this key conceptual distinction.

And technically, as the cell body enlarges during wave formation, often extending over the proximal base of adjacent neurites, the soma-neurite boundary can be blurred. Therefore, a strict segmentation between soma and proximal neurite would be artificial and not biologically meaningful for this analysis.

To reflect the location of wave formation more precisely, we will revise the text to state that waves emerge "at the perisomatic region."

Page 5, line 100: "*unpolarized neurons in cortical slices repeatedly formed actin waves at the <u>perisomatic region</u> (**Fig. 1i**).*"

Page 6, line 129: *"unpolarized neurons transiently polarize when one neurite acquires the wave from the <u>perisomatic region</u> followed by extension, which then retracts again."*

4) All examples shown were of developing neurons that already had minor processes. Prior to any minor process formation, do neuronal cell bodies show many waves that initiate minor processes?

In neurons at stage 1, prior to the formation of minor processes, we observed broad, circumferential wave-like F-actin structures at the soma. These wave-like structures dynamically fluctuate around the cell perimeter and give rise to transient micro-processes. Over time, wave activity becomes preferentially localized to a subset of these micro-processes, some of which subsequently stabilize and develop into neurites. We will include a representative stage-1 video to illustrate this behavior and revise the text to explicitly describe this early-stage dynamic.

It is noteworthy that the initial formation of neurites depends on Actin depolymerizing factor (ADF)/Cofilin, as we previously showed: neurons lacking ADF/Cofilin fail to form neurites at all[1]. Such a phenotype is mechanistically distinct from the loss-of-Arp3 phenotype: Arp3 KO neurons form neurites but fail to polarize. This distinction underscores that our study addresses the key step of neuronal polarization when neurites had formed. We will make this distinction explicit in the revised text.

5) Figure 2 correlates sub-cellular actin puncta (patches) with wave formation. Small puncta appear all over the cell body with no clear bias toward where a wave forms. Also, in figure 2a, waves initiate within the proximal neurite, so it is not clear how this correlates with actin patches.

We agree with reviewer #3 that actin patches are broadly distributed throughout the soma without an obvious spatial bias toward the eventual site of wave emergence. We did not intend to claim that the location of wave formation at the perisomatic region is correlated with the patch density in that region. Rather, our data demonstrate a temporal and quantitative relationship between somatic actin patch intensity and wave emergence (**Fig. 2c, 2d** and **Extended Data Fig. 2a**): actin patch intensity increases over time at the soma until a wave is triggered, after which patch intensity rapidly decreases and then gradually rebuilds. Of note, actin patches can be observed also along the neurite shafts, but actin waves rarely emerge there.

Thus, somatic actin patches and actin waves are functionally linked actin structures regulated by Arp2/3, reflecting a cell-intrinsic buildup-and-release dynamic. Conceptually, this represents a transition from a non-polarized state (gradual accumulation of actin patches at the soma) to a transiently polarized state (wave formation and selective propagation into a single neurite).

6) Figure 3 shows that Arp2/3 is responsible for F-actin waves, which is expected as Arp2/3 is known to promote branched actin networks.

While Arp2/3 is well established as a nucleator of branched F-actin networks, the findings in **Figure 3** extend substantially beyond this canonical role.

The conceptual advance from **Figure 3** is that Arp2/3 operates as a spatiotemporally regulated, location-dependent activator that gives neurons an intrinsic bias toward polarization.

The key findings of **Figure 3** include:

First, the increase of Arp3-actin patches precedes neurite extension, indicating a growth-priming role of Arp2/3 activity (**Fig. 3a-c**).

Second, global pharmacological CK-666 treatment (**Fig. 3d-i**) shows that Arp2/3 controls neurite growth dynamics prior to polarization, and that this dependence becomes asymmetric following polarization. Upon polarization, axons progressively become resistant to CK-666-induced retraction whereas minor neurites remain sensitive.

Third, local activation of Arp2/3 using photo-activatable (PA)-Rac1 (**Fig. 3j-n**) demonstrates that Arp2/3 activity exerts location-dependent effects, supporting a direct coupling between actin wave translocation and oscillatory neurite growth.

Fourth, local CK-666 perfusion (**Fig. 3o-r**) shows that wave initiation requires local Arp2/3 activity: neurites within the perfused region retract, whereas neurites outside the perfused region can still initiate retrograde waves from their growth cones.

Thus, **Figure 3** redefines Arp2/3 not simply as a structural actin nucleator for wave formation, but as a spatiotemporal organizer that links cytoskeletal dynamics to the emergence of neuronal polarity.

7) Figure 4 is nice, as it shows neuronal polarization is disrupted in Arp3 mutants in vivo, although this is not unexpected.

We appreciate this reviewer's positive comment. However, we respectfully disagree that this finding is expected. Previous studies in cultured neurons have reported conflicting conclusions regarding the role of Arp2/3 in neurite and axon growth, which has hampered the field for many years. Some reports found that Arp2/3 inhibits neurite or axon extension[9,10] whereas other studies concluded that Arp2/3 promotes neurite/axon growth[11–13]. Importantly, these studies relied on molecular perturbations or global pharmacological approaches, limiting insight into the physiological role of Arp2/3 in intact systems.

Therefore, the physiological role of Arp2/3 in neuronal polarization has remained unresolved. **Figure 4** addresses this gap directly by demonstrating that Arp2/3 depletion abolishes cell-autonomous polarization in sparsely targeted cortical neurons *in vivo*. This phenotype was consistently reproduced across Arp3 KO cortical slices and cultured Arp3 KO neurons, providing convergent evidence across experimental systems.

Taken together, these results establish Arp2/3 as a necessary and positive physiological regulator of neuronal polarization *in vivo*. We therefore view **Figure 4** as a central result that resolves prior discrepancies and anchors our mechanistic model in a physiological context.

To make this clearer, we will stress this even more in the motivation of using a genetic knockout mouse model in the manuscript:

Page 12, line 259:

Before revision: "*Arp2/3 has been reported to both promote and inhibit axon growth in vitro*[9,10,14]*, but these studies relied on molecular manipulations of Arp2/3 that may have altered the complex's localization.*"

After revision: "*Arp2/3 has been reported to both promote and inhibit axon growth in vitro*[9,10,14]*, but these studies relied on molecular manipulations of Arp2/3 that may have altered the complex's localization<u>, limiting insight into the physiological role of Arp2/3</u>.*"

8) On line 174 the authors state that "the original polarity was largely preserved upon recovery" after washout of CK666. How does the wave model account for this finding?

A central concept of our model is that the establishment of polarity and the maintenance of polarity are mediated by distinct, though interconnected, mechanisms. The resistance of mature axon to CK-666-induced retraction, the decrease of waves in the established axon, and the preservation of original polarity after CK-666 washout all indicate that Arp2/3 activity is not required for the maintenance, or "memory", of polarity once it has been established.

Our study shows that the Arp2/3–actomyosin oscillator biases neurite selection during the fluctuating pre-polarization stage, after which polarity is consolidated downstream, involving microtubule stabilization[15,16]. This interpretation is consistent with the washout result that most neurons re-established the original axon. We will revise the Discussion to more explicitly distinguish between these two phases of polarity establishment and maintenance.

Page 18, line 396:

Before revision: *"Polarization is then consolidated through mechanisms that stabilize microtubules."*

After revision: *"<u>Once the polarity is established through oscillation</u>, polarization is then consolidated through mechanisms that stabilize microtubules."*

9) Finally, the authors make the blanket statement that "Indeed, growth cones of embryonic CNS neurons do not pull on 3-dimensional soft matrices to move forward". This may be true for some CNS neurons in some circumstances, but it is highly likely that some CNS neurons (eg retinal ganglion cells and spinal commissural neurons) extend by traditional mesenchymal based cell migration) by pulling on soft substrates (see papers by David Odde (eg Traction Dynamics of Filopodia on Compliant Substrates) and others).

We thank the reviewer for this comment. We agree that this statement was unnecessarily broad and did not sufficiently reflect neuron type-specific differences. As the reviewer suggests, we will revise the sentence by specifying the neuronal populations examined in our study. The sentence will read as follows:

Page 18, line 400:

Before revision: *"Indeed, growth cones of embryonic CNS neurons do not pull on 3-dimensional soft matrices to move forward."*

After revision: *"Indeed, growth cones of <u>embryonic cortical and hippocampal neurons</u> do not pull on 3-dimensional soft matrices to move forward."*

We will also clarify in the Discussion that other neuron types may employ traction-based mechanisms under certain conditions, as highlighted by the reviewer.

References

1. Flynn, K. C., Pak, C. W., Shaw, A. E., Bradke, F. & Bamburg, J. R. Growth cone-like waves transport actin and promote axonogenesis and neurite branching. *Dev. Neurobiol.* **69**, 761–779 (2009).

2. Winans, A. M., Collins, S. R. & Meyer, T. Waves of actin and microtubule polymerization drive microtubule-based transport and neurite growth before single axon formation. *eLife* **5**, e12387 (2016).

3. Charras, G. T. A short history of blebbing. *J. Microsc.* **231**, 466–478 (2008).

4. García-Arcos, J. M., Jha, A., Waterman, C. M. & Piel, M. Blebology: principles of bleb-based migration. *Trends Cell Biol.* (2024) doi:10.1016/j.tcb.2024.02.009.

5. Ghosh, I., Singh, R. K., Mishra, M., Kapoor, S. & Jana, S. S. Switching between blebbing and lamellipodia depends on the degree of non-muscle myosin II activity. *J. Cell Sci.* **134**, (2021).

6. Bergert, M., Chandradoss, S. D., Desai, R. A. & Paluch, E. Cell mechanics control rapid transitions between blebs and lamellipodia during migration. *Proc Natl Acad Sci USA* **109**, 14434–14439 (2012).

7. Fackler, O. T. & Grosse, R. Cell motility through plasma membrane blebbing. *J. Cell Biol.* **181**, 879–884 (2008).

8. Glaser, K. M. *et al.* Arp2/3 complex and the pentose phosphate pathway regulate late phases of neutrophil swarming. *iScience* **27**, 108656 (2024).

9. Strasser, G. A., Rahim, N. A., VanderWaal, K. E., Gertler, F. B. & Lanier, L. M. Arp2/3 is a negative regulator of growth cone translocation. *Neuron* **43**, 81–94 (2004).

10. Ikeno, T. & Konishi, Y. Arp2/3 is required for axonal arbor terminal retraction in cerebellar granule neurons. *Neurochem. J.* **14**, 32–36 (2020).

11. Kunda, P., Paglini, G., Quiroga, S., Kosik, K. & Caceres, A. Evidence for the involvement of Tiam1 in axon formation. *J. Neurosci.* **21**, 2361–2372 (2001).

12. Tahirovic, S. *et al.* Rac1 regulates neuronal polarization through the WAVE complex. *J. Neurosci.* **30**, 6930–6943 (2010).

13. Montenegro-Venegas, C. *et al.* MAP1B regulates axonal development by modulating Rho-GTPase Rac1 activity. *Mol. Biol. Cell* **21**, 3518–3528 (2010).

14. San Miguel-Ruiz, J. E. & Letourneau, P. C. The role of Arp2/3 in growth cone actin dynamics and guidance is substrate dependent. *J. Neurosci.* **34**, 5895–5908 (2014).

15. Witte, H., Neukirchen, D. & Bradke, F. Microtubule stabilization specifies initial neuronal polarization. *J. Cell Biol.* **180**, 619–632 (2008).

16. Gomis-Rüth, S., Wierenga, C. J. & Bradke, F. Plasticity of polarization: changing dendrites into axons in neurons integrated in neuronal circuits. *Curr. Biol.* **18**, 992–1000 (2008).

Response to the reviewers' comments on Nature 2023-11-21494A-Z:

"An intrinsic cytoskeletal oscillator establishes neuronal polarity"

We thank the reviewers for this additional round of constructive critique. In the following, we address these comments point-by-point.

The reviewer's comments are in blue font, our responses are in black font, and new text added to the manuscript is in black italic font.

Reviewer #1

In their revised manuscript, the authors have addressed all my initial concerns and added an impressive amount of new data. The new results on the local activation/inhibition of Arp2/3 are quite convincing and support their model.

I would suggest to say on the opening frame of each movie, to which figure panel it is related and also provide the movie number. It took me quite some time to find out.

We thank reviewer #1 for the positive assessment of our revised manuscript and for the advice on the supplementary videos. We have now added a label on the opening frame of each Supplementary Video indicating the related figure panel and the video number.

Reviewer #2

The revised manuscript by Lin and colleagues have been significantly improved with additional experiments and careful editing. Extensive sets of new data provide strong support for the claim that cell-intrinsic oscillatory actin cytoskeletal dynamics underlie the formation of a single axon in a developing neuron with further mechanistic insights, and validation in cortical brain slice cultures also adds strength. The authors have suitably addressed my prior concerns in the revision. The main findings would be of broad interest.

We thank reviewer #2 for the positive assessment of our revised manuscript and for the constructive suggestions, which we have addressed as follows.

Minor comments:

1) Figure 1d is redundant with panel b. It would be more informative to show an inset of a representative time window with an expanded time scale to highlight the sequential extension and retraction events by the labelled neurites.

   We have replaced **Fig. 1d** with a graph of selected time window and representative frames, allowing the sequential extension and retraction events of the labelled neurites to be more clearly appreciated.

   Original: Revised:

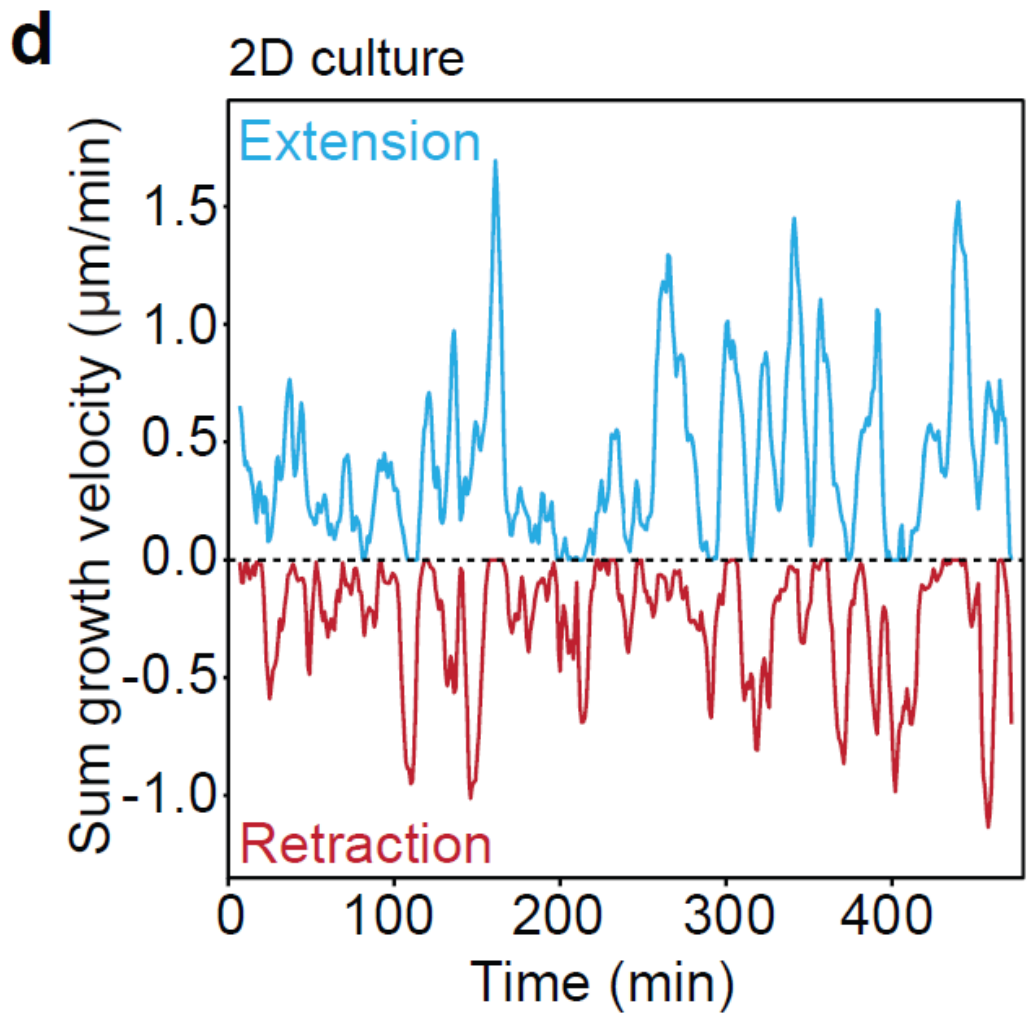

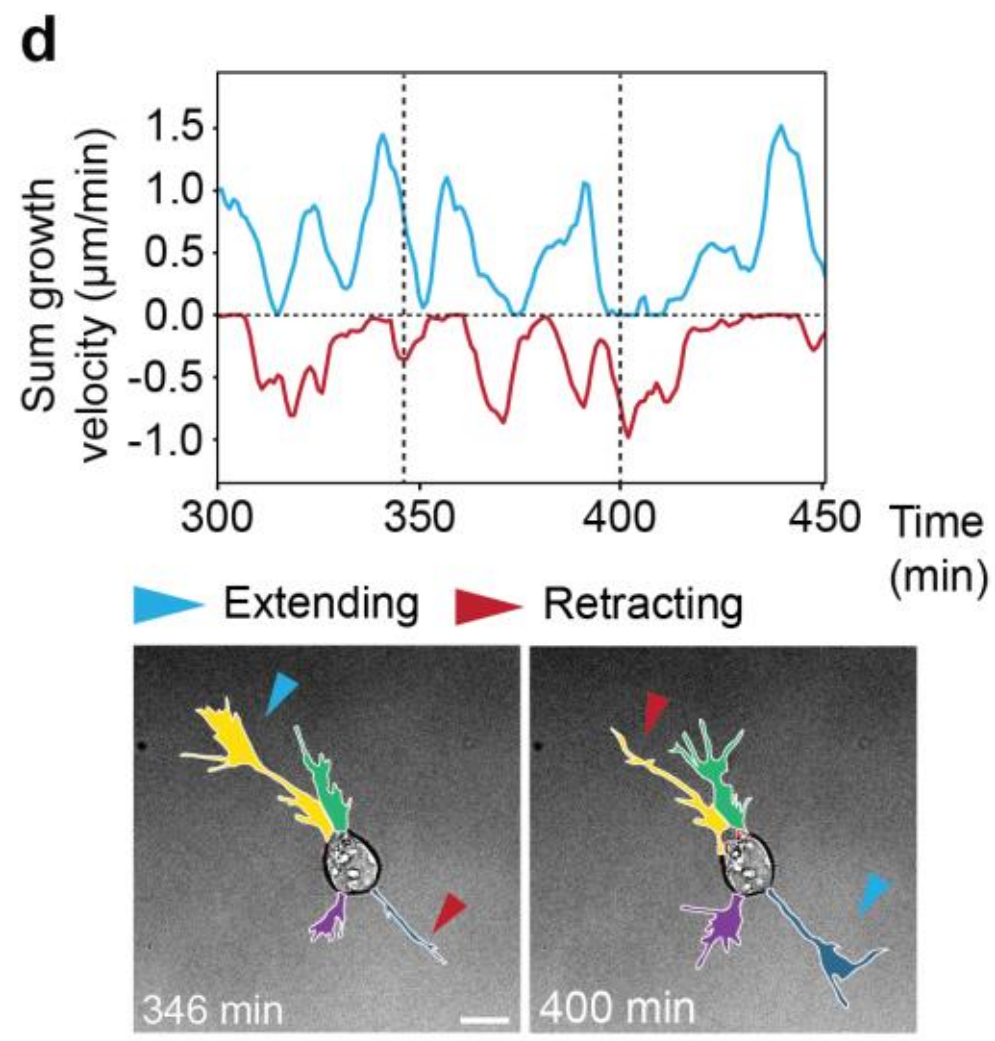

2) Line 98. When introducing the use of cortical slices, it should be made clear that the preparation refers to organotypic culture and/or that slices have been cultured for 30 h. The clarification is not only important in interpreting the data, but also in avoiding the confusion since the authors also use acutely sliced preparation (if I understood it correctly) referred to as transections in other experiments (e.g. Fig 4e-h).

We thank the reviewer for prompting us to clarify the slice preparation. The organotypic cortical slices shown in **Fig. 1c** and **1h** were imaged on the same day of slicing; the slices were cultured for 4-8 h before imaging, not 30 h. We have clarified this in the Methods section and have revised the text:

Page 5, line 98: "*……by time-lapse imaging of these neurons in acute cortical slice.*"

Page 30, line 638: "*Serial snapshots of unpolarized neurons expressing Lifeact-mNeonGreen in E15.5 acute cortical slice*"

Page 45, line 1023: "*After incubating at 35ºC and 5% CO_2 for 4-8 hours, slices were imaged with……*"

Reviewer #3

I have re-reviewed the paper by Lin et al., and I am afraid my opinion has not changed. This is a very nice cell biological study that describes details of neuron polarization processes. The most impactful conclusion according to authors description is that a "soma-initiated oscillatory program primes axon selection", which contrasts with the growth cone centric held view previously regarding polarization. This conclusion is based on extensive analysis of live cell behaviors together with pharmacological and molecular manipulations. While this study is extremely detailed, its overall impact, combined with several minor concerns, reduces my enthusiasm for publication in Nature. I will list a few concerns below.

We appreciate the reviewer's recognition of our study and for pointing out "several minor concerns", which has helped us further improve the manuscript as described point-by-point below. While this reviewer considers our work as "a very nice cell biological study", we want to use this opportunity to clarify the overall impact of our work.

Neuronal polarization is not a niche phenomenon; it is the structural basis of directional information flow in the brain. It underlies how the neuron establishes a single axon, a long-standing and fundamental question in cell biology, development, and neurobiology.

For decades, this process has been largely interpreted through a growth cone-centric framework. As reviewer #3 acknowledged, we overturn this view and identify a soma-initiated oscillatory mechanism, which redefines how we understand this principled feature of circuit architecture.

The impact of our study is further strengthened by uncovering a cytoskeletal activator-inhibitor mechanism that translates these oscillations into neuronal polarization. This conceptual advance brings a broader principle to developmental and cell biology — spontaneous symmetry breaking through an oscillatory cytoskeletal mechanism. This framework provides a mechanistic explanation for how neurons reliably form a single axon even in the presence of abundant growth-promoting signals. The discovered symmetry-breaking mechanism underpins the key concept that neuronal polarization is cell-intrinsic rather than external cue-dependent. Together, these findings establish a generalizable principle with implications across neuroscience, cell biology, and developmental biology.

1) It is stated on Line 101 "Strikingly, we found that upon wave formation, adjacent neurites rapidly retracted". I do not see any change of adjacent neurites after wave enters primary neurite in figure or movie.

   We thank the reviewer for prompting us to clarify this point. In **Fig. 1h** and **Supplementary Video 3**, the neurite adjacent to the wave formation site at the soma is the same one that received the wave: at 70 min, the neurite started to retract during wave formation at the soma; at 76 min, the wave entered the same neurite and initiated regrowth; at 82 min, the neurite retracted again during wave formation at the soma.

   Therefore, what we observed is that the retraction of adjacent neurites is associated with wave formation at the soma, not with the wave entry into the receiving neurite or with the subsequent extension. In other words, the neurite retraction and the wave formation at the soma are temporally overlapping events.

   In 2D cultures, we were able to resolve the event order with cross-correlation analysis: the increase of somatic F-actin (pre-wave formation) precedes the acceleration of neurite retraction by 4 min (**Fig. 2e**). The correlation between velocities of extending and retracting neurites (**Fig. 1e-f**) also showed that neurite retraction precedes, not coincides with, extension (by ~20 min in 2D culture and ~45 min in cortical slices).

   To clarify this, we added reference markers in **Fig. 1h** and **Supplementary Video 3** to indicate initial tip positions and revise the text:

   Page 5, line 101: "*We found wave formation at the soma was associated with retraction of the neurite adjacent to the wave-forming site.*"

2) Moreover, the "waves" in cell bodies appear subtle (some appear to be blebs without increased lifeact intensity). It is not clear how these are subjectively scored as waves.

   The definition of actin waves through the manuscript and in other studies is growth-cone-like F-actin structures that translocate toward and along a neurite[1,2]. In the live-cell imaging of neurons expressing Lifeact, the actin wave and its translocation can be reliably identified based on coordinated changes in local morphology and F-actin intensity (e.g. **Fig. 2a, 2g** and **Extended Data Fig. 2h**; **Supplementary Videos 5, 6** and **8**).

   A technical note: In live-cell imaging of cortical slices, where the neurons are embedded in 3D environment, the nascent actin waves at the soma are partially captured as thin veils with lower Lifeact intensity. In contrast, in the 2D cultured neurons, actin waves cannot go beyond the glass bottom and thus the full wave structure is detected with high Lifeact intensity. Thereby, waves show higher actin intensities in 2D than in 3D environments. Accordingly, in slice preparations, we rely on both morphological dynamics and F-actin signal to identify actin waves.

   We can rule out that the waves at the soma shown in **Fig. 1h** and **Supplementary Video 3** are blebbing events for the following reasons: First, cellular blebs can expand and retract but, to our knowledge, do not exhibit directional translocation along neurites as actin waves do[3,4]. Second, we showed that in Arp3 KO neurons, actin waves were abolished (**Extended Data Fig. 9c; Supplementary Video 24**) and actomyosin was upregulated (**Fig. 4l-m; Extended Data Fig. 9h**). Consistently, inhibiting myosin

II with blebbistatin increased the occurrence of waves (**Extended Data Fig. 12j**)[1]. In contrast, cellular blebs are induced by upregulated actomyosin but suppressed by blebbistatin[5,6]. And third, blebbing is reported to be independent of Arp2/3 activity[7,8], further distinguishing it from the actin waves described here.

3) Moreover and most importantly, often waves appear to form first in proximal processes near the cell body (Fig 2a). Are these also considered cell soma waves?

We agree that the actin wave formation in **Fig. 2a** appears at the soma–proximal neurite interface rather than strictly within the soma. Indeed, actin waves can also emerge at or near this interface, which is consistent with our model. We established the temporal and quantitative relationship between somatic actin patch intensities and wave emergence as shown in **Fig. 2c, 2d** and **Extended Data Fig. 2a**: actin patch intensity builds up at the soma until a wave is triggered, after which patch intensity drops and gradually rebuilds. Importantly, this temporal sequence indicates that the initiating event is somatic, even when the visible wavefront appears at the proximal neurite base.

In addition, to address the central debate in neuronal polarization, the mechanistically relevant comparison is between the perisomatic region and the distal growth cones; finer sub-regional distinctions (e.g., soma versus proximal base) do not alter this key conceptual distinction.

And technically, as the cell body enlarges during wave formation, often extending over the proximal base of adjacent neurites, the soma-neurite boundary can be blurred. Therefore, a strict segmentation between soma and proximal neurite would be artificial and not biologically meaningful for this analysis.

To reflect the location of wave formation more precisely, we revised the text to state that waves emerge "at the perisomatic region."

Page 5, line 100: "*unpolarized neurons in cortical slices repeatedly formed actin waves at the perisomatic region* ***(Fig. 1i)***."

Page 6, line 130: *"unpolarized neurons transiently polarize when one neurite acquires the wave from the perisomatic region followed by extension, which then retracts again."*

4) All examples shown were of developing neurons that already had minor processes. Prior to any minor process formation, do neuronal cell bodies show many waves that initiate minor processes?

In neurons at stage 1, prior to the formation of minor processes, we observed broad, circumferential wave-like F-actin structures at the soma. These wave-like structures dynamically fluctuate around the cell perimeter and give rise to transient micro-processes. Over time, wave activity becomes preferentially localized to a subset of these micro-processes, some of which subsequently stabilize and develop into neurites.

It is noteworthy that the initial formation of neurites depends on Actin depolymerizing factor (ADF)/Cofilin, as we previously showed: neurons lacking ADF/Cofilin fail to form neurites at all[1]. Such a phenotype is mechanistically distinct from the loss-of-Arp3 phenotype: Arp3 KO neurons form neurites but fail to polarize. This distinction underscores that our study addresses the key step of neuronal polarization when neurites had formed. We made this distinction explicit in the revised text.

Page 13, line 281: "*Notably, neurons lacking actin depolymerizing factor (ADF)/cofilin fail to form neurites at all[63], a phenotype distinct from Arp3 KO neurons. Thus, Arp2/3 is specifically required for polarization, not neurite initiation.*"

Page 18, line 387: "*This process is distinct from neurite initiation itself, which depends on ADF/cofilin-mediated actin turnover[63].*"

5) Figure 2 correlates sub-cellular actin puncta (patches) with wave formation. Small puncta appear all over the cell body with no clear bias toward where a wave forms. Also, in figure 2a, waves initiate within the proximal neurite, so it is not clear how this correlates with actin patches.

We agree with reviewer #3 that actin patches are broadly distributed throughout the soma without an obvious spatial bias toward the eventual site of wave emergence. We did not intend to claim that the location of wave formation at the perisomatic region is correlated with the patch density in that region. Rather, our data demonstrate a temporal and quantitative relationship between somatic actin patch intensity and wave emergence (**Fig. 2c, 2d** and **Extended Data Fig. 2a**): actin patch intensity increases over time at the soma until a wave is triggered, after which patch intensity rapidly decreases and then gradually rebuilds. Of note, actin patches can be observed also along the neurite shafts, but actin waves rarely emerge there.

Thus, somatic actin patches and actin waves are functionally linked actin structures regulated by Arp2/3, reflecting a cell-intrinsic buildup-and-release dynamic. Conceptually, this represents a transition from a non-polarized state (gradual accumulation of actin patches at the soma) to a transiently polarized state (wave formation and selective propagation into a single neurite).

6) Figure 3 shows that Arp2/3 is responsible for F-actin waves, which is expected as Arp2/3 is known to promote branched actin networks.

While Arp2/3 is well established as a nucleator of branched F-actin networks, the findings in **Figure 3** extend substantially beyond this canonical role.

The conceptual advance from **Figure 3** is that Arp2/3 operates as a spatiotemporally regulated, location-dependent activator that gives neurons an intrinsic bias toward polarization.

The key findings of **Figure 3** include:

First, the increase of Arp3-actin patches precedes neurite extension, indicating a growth-priming role of Arp2/3 activity (**Fig. 3a-c**).

Second, global pharmacological CK-666 treatment (**Fig. 3d-i**) shows that Arp2/3 controls neurite growth dynamics prior to polarization, and that this dependence becomes asymmetric following polarization. Upon polarization, axons progressively become resistant to CK-666-induced retraction whereas minor neurites remain sensitive.

Third, local activation of Arp2/3 using photo-activatable (PA)-Rac1 (**Fig. 3j-n**) demonstrates that Arp2/3 activity exerts location-dependent effects, supporting a direct coupling between actin wave translocation and oscillatory neurite growth.

Fourth, local CK-666 perfusion (**Fig. 3o-r**) shows that wave initiation requires local Arp2/3 activity: neurites within the perfused region retract, whereas neurites outside the perfused region can still initiate retrograde waves from their growth cones.

Thus, **Figure 3** redefines Arp2/3 not simply as a structural actin nucleator for wave formation, but as a spatiotemporal organizer that links cytoskeletal dynamics to the emergence of neuronal polarity.

7) Figure 4 is nice, as it shows neuronal polarization is disrupted in Arp3 mutants in vivo, although this is not unexpected.

We appreciate this reviewer's positive comment. However, we respectfully disagree that this finding is expected. Previous studies in cultured neurons have reported conflicting conclusions regarding the role of Arp2/3 in neurite and axon growth, which has hampered the field for many years. Some reports found that Arp2/3 inhibits neurite or axon extension[9,10] whereas other studies concluded that Arp2/3 promotes neurite/axon growth[11–13]. Importantly, these studies relied on molecular perturbations or global pharmacological approaches, limiting insight into the physiological role of Arp2/3 in intact systems.

Therefore, the physiological role of Arp2/3 in neuronal polarization has remained unresolved. **Figure 4** addresses this gap directly by demonstrating that Arp2/3 depletion abolishes cell-autonomous polarization in sparsely targeted cortical neurons *in vivo*. This phenotype was consistently reproduced across Arp3 KO cortical slices and cultured Arp3 KO neurons, providing convergent evidence across experimental systems.

Taken together, these results establish Arp2/3 as a necessary and positive physiological regulator of neuronal polarization *in vivo*. We therefore view **Figure 4** as a central result that resolves prior discrepancies and anchors our mechanistic model in a physiological context.

To make this clearer, we now stress this even more in the motivation of using a genetic knockout mouse model in the manuscript:

Page 12, line 260:

Before revision: "*Arp2/3 has been reported to both promote and inhibit axon growth in vitro*[9,10,14]*, but these studies relied on molecular manipulations of Arp2/3 that may have altered the complex's localization.*"

After revision: "*Arp2/3 has been reported to both promote and inhibit axon growth in vitro*[9,10,14]*, but these studies relied on molecular manipulations of Arp2/3 that may have altered the complex's localization, limiting insight into the physiological role of Arp2/3.*"

8) On line 174 the authors state that "the original polarity was largely preserved upon recovery" after washout of CK666. How does the wave model account for this finding?

A central concept of our model is that the establishment of polarity and the maintenance of polarity are mediated by distinct, though interconnected, mechanisms. The resistance of mature axon to CK-666-induced retraction, the decrease of waves in the established axon, and the preservation of original polarity after CK-666 washout all indicate that Arp2/3 activity is not required for the maintenance, or "memory", of polarity once it has been established.

Our study shows that the Arp2/3–actomyosin oscillator biases neurite selection during the fluctuating pre-polarization stage, after which polarity is consolidated downstream, involving microtubule stabilization[15,16]. This interpretation is consistent with the washout result that most neurons re-established the original axon. We revised the Discussion to more explicitly distinguish between these two phases of polarity establishment and maintenance.

Page 18, line 400:

Before revision: *"Polarization is then consolidated through mechanisms that stabilize microtubules."*

After revision: *"Once the polarity is established through oscillation, polarization is then consolidated through mechanisms that stabilize microtubules."*

9) Finally, the authors make the blanket statement that "Indeed, growth cones of embryonic CNS neurons do not pull on 3-dimensional soft matrices to move forward". This may be true for some CNS neurons in some circumstances, but it is highly likely that some CNS neurons (eg retinal ganglion cells and spinal commissural neurons) extend by traditional mesenchymal based cell migration) by pulling on soft substrates (see papers by David Odde (eg Traction Dynamics of Filopodia on Compliant Substrates) and others).

We thank the reviewer for this comment. We agree that this statement was unnecessarily broad and did not sufficiently reflect neuron type-specific differences. As the reviewer suggests, we revised the sentence by specifying the neuronal populations examined in our study.

Page 19, line 404:

Before revision: *"Indeed, growth cones of embryonic CNS neurons do not pull on 3-dimensional soft matrices to move forward."*

After revision: *"Indeed, growth cones of embryonic cortical and hippocampal neurons do not pull on 3-dimensional soft matrices to move forward."*

We also clarified in the Discussion that other neuron types may employ traction-based mechanisms under certain conditions, as highlighted by the reviewer.

References

1. Flynn, K. C., Pak, C. W., Shaw, A. E., Bradke, F. & Bamburg, J. R. Growth cone-like waves transport actin and promote axonogenesis and neurite branching. *Dev. Neurobiol.* **69**, 761–779 (2009).

2. Winans, A. M., Collins, S. R. & Meyer, T. Waves of actin and microtubule polymerization drive microtubule-based transport and neurite growth before single axon formation. *eLife* **5**, e12387 (2016).

3. Charras, G. T. A short history of blebbing. *J. Microsc.* **231**, 466–478 (2008).

4. García-Arcos, J. M., Jha, A., Waterman, C. M. & Piel, M. Blebology: principles of bleb-based migration. *Trends Cell Biol.* (2024) doi:10.1016/j.tcb.2024.02.009.

5. Ghosh, I., Singh, R. K., Mishra, M., Kapoor, S. & Jana, S. S. Switching between blebbing and lamellipodia depends on the degree of non-muscle myosin II activity. *J. Cell Sci.* **134**, (2021).

6. Bergert, M., Chandradoss, S. D., Desai, R. A. & Paluch, E. Cell mechanics control rapid transitions between blebs and lamellipodia during migration. *Proc Natl Acad Sci USA* **109**, 14434–14439 (2012).

7. Fackler, O. T. & Grosse, R. Cell motility through plasma membrane blebbing. *J. Cell Biol.* **181**, 879–884 (2008).

8. Glaser, K. M. *et al.* Arp2/3 complex and the pentose phosphate pathway regulate late phases of neutrophil swarming. *iScience* **27**, 108656 (2024).

9. Strasser, G. A., Rahim, N. A., VanderWaal, K. E., Gertler, F. B. & Lanier, L. M. Arp2/3 is a negative regulator of growth cone translocation. *Neuron* **43**, 81–94 (2004).

10. Ikeno, T. & Konishi, Y. Arp2/3 is required for axonal arbor terminal retraction in cerebellar granule neurons. *Neurochem. J.* **14**, 32–36 (2020).

11. Kunda, P., Paglini, G., Quiroga, S., Kosik, K. & Caceres, A. Evidence for the involvement of Tiam1 in axon formation. *J. Neurosci.* **21**, 2361–2372 (2001).

12. Tahirovic, S. *et al.* Rac1 regulates neuronal polarization through the WAVE complex. *J. Neurosci.* **30**, 6930–6943 (2010).

13. Montenegro-Venegas, C. *et al.* MAP1B regulates axonal development by modulating Rho-GTPase Rac1 activity. *Mol. Biol. Cell* **21**, 3518–3528 (2010).

14. San Miguel-Ruiz, J. E. & Letourneau, P. C. The role of Arp2/3 in growth cone actin dynamics and guidance is substrate dependent. *J. Neurosci.* **34**, 5895–5908 (2014).

15. Witte, H., Neukirchen, D. & Bradke, F. Microtubule stabilization specifies initial neuronal polarization. *J. Cell Biol.* **180**, 619–632 (2008).

16. Gomis-Rüth, S., Wierenga, C. J. & Bradke, F. Plasticity of polarization: changing dendrites into axons in neurons integrated in neuronal circuits. *Curr. Biol.* **18**, 992–1000 (2008).
