# Supplementary material for: An intrinsic cytoskeletal oscillator establishes neuronal polarity
Source: Nature. 2026 Jul 8;657(8130):213–25. doi: 10.1038/s41586-026-10755-6 (PMC13538037; doi:10.1038/s41586-026-10755-6)
Supplement: Supplementary file 4 [file 41586_2026_10755_MOESM3_ESM.docx]

**Supplementary Table 1. Primary antibodies used in this study and validation evidence.**

All primary antibodies used in this study were validated for the species and application in which they were used. Validation evidence includes manufacturer datasheets, peer-reviewed citations (CiteAb antibody database, *https://www.citeab.com/*), and where applicable, in-house genetic validation by loss-of-function in this study. Antibodies against the Arp2/3 complex subunits (Arp2, ARPC1A, ARPC2, ARPC3, ARPC4, ARPC5) were validated in-house by reduction of signal in *Actr3* conditional knockout cortical lysates (Fig. 4b, c).

| **Target** | **Catalog #** | **Supplier** | **Host / Clonality** | **Species reactivity** | **Application in this study** | **Validation evidence** | **Reference figures** |
| --- | --- | --- | --- | --- | --- | --- | --- |
| **Arp3** | 07-272 | Millipore (Upstate) | Rabbit polyclonal | Human, mouse, rat | WB, ICC | Manufacturer datasheet: WB validated, single band at ~47 kDa.  Knockout-validated in this study by loss of signal in Actr3 conditional KO cortical lysates and neurons. Cited in 9 peer-reviewed publications (CiteAb). | Fig. 3a;  Fig. 4b, c;  Ext. Data Fig. 9a |
| **Arp3** | A5979 | Sigma-Aldrich | Mouse monoclonal (FMS338) | Human, mouse, rat | WB | Manufacturer datasheet: validated by WB and ICC.  Used in this study as orthogonal Arp3 antibody for ICC; complemented Millipore 07-272 used for WB. | Ext. Data Fig. 9h |
| **Arp2** | 5614S | Cell Signaling Technology | Rabbit monoclonal (D85D5, XP) | Human, mouse, rat | WB | Manufacturer datasheet: rigorously validated in-house by WB; recognizes endogenous Arp2; does not cross-react with Arp3.  Specificity confirmed in this study by reduction of signal in Actr3 conditional KO lysates (consistent with co-destabilization of Arp2/3 complex subunits upon Arp3 loss). | Fig. 4b, c |
| **ARPC1A** | HPA004334 | Sigma-Aldrich (Atlas / Prestige) | Rabbit polyclonal | Human (validated); cross-reactivity inferred for mouse | WB | Human Protein Atlas Prestige antibody, validated by WB, ICC and IHC against the human proteome.  Specificity in this study supported by reduction of signal in Actr3 conditional KO lysates (Arp2/3 subunit co-destabilization). | Fig. 4c |
| **ARPC2** | 07-227 | Millipore (Upstate) | Rabbit polyclonal | Human, mouse, rat | WB | Manufacturer datasheet: validated by WB, ICC, IHC. Cited in 90+ peer-reviewed publications (CiteAb).  Specificity in this study supported by reduction of signal in Actr3 conditional KO lysates (Arp2/3 subunit co-destabilization). | Fig. 4b, c |
| **ARPC4** | sc-68394 | Santa Cruz Biotechnology | Mouse monoclonal | Mouse, rat, human | WB | Manufacturer datasheet: validated by WB.  Specificity in this study supported by reduction of signal in Actr3 conditional KO lysates (Arp2/3 subunit co-destabilization). | Fig. 4c |
| **ARPC5** | sc-166760 | Santa Cruz Biotechnology | Mouse monoclonal (C-3) | Mouse, rat, human | WB | Manufacturer datasheet: validated by WB; cited in published studies (CiteAb).  Specificity in this study supported by reduction of signal in Actr3 conditional KO lysates. | Fig. 4b, c |
| **Tau-1** | MAB3420 | Millipore (Chemicon) | Mouse monoclonal (PC1C6, IgG2a) | Bovine, human, rat (validated); mouse (used in this study) | ICC, IHC (axon marker) | Manufacturer datasheet: validated by WB and IHC; recognizes dephosphorylated Tau (Ser195/198/199/202). Cited in 65+ publications (CiteAb).  Established axon marker in neuronal polarization research, consistent with seminal use by Dotti & Banker. | Fig. 3f;  Fig. 4e, i;  Fig. 5a, i;  Ext. Data Fig. 4g;  Ext. Data Fig. 8a, l, o;  Ext. Data Fig. 12e, k |
| **β-tubulin III (TUJ1)** | T2200 | Sigma-Aldrich | Rabbit polyclonal | Human, mouse, rat | ICC (neurite/microtubule marker) | Manufacturer datasheet: validated by WB, ICC, IHC, IP. Cited in 580+ publications (CiteAb). Single band at ~50 kDa; specificity confirmed against neuronal lysates. | Fig. 3f;  Fig. 4i;  Fig. 5a, i;  Ext. Data Fig. 4g;  Ext. Data Fig. 8l, o;  Ext. Data Fig. 11a;  Ext. Data Fig. 12e, k |
| **Non-muscle myosin IIb (MYH10)** | 8824 | Cell Signaling Technology | Rabbit monoclonal (D8H8, XP) | Human, mouse, rat | ICC | Manufacturer datasheet: validated in-house by WB and IF; recognizes endogenous total myosin IIb; does not cross-react with myosin IIa or IIc isoforms. Confirmed isoform specificity per Cell Signaling validation pipeline. | Fig. 4l, m;  Ext. Data Fig. 6f, g |
| **Phospho-MRLC (Ser19)** | ab2480 | Abcam | Rabbit polyclonal | Human, mouse, rat, C. elegans, Xenopus | WB | Manufacturer datasheet: validated by WB, IP, IHC-P, ICC/IF; <1% cross-reactivity with non-phosphorylated MRLC by ELISA. Cited in 140+ publications (CiteAb).  Specificity in this study supported by Western blot at expected MW with WT/KO comparison. | Ext. Data Fig. 9h, i |
| **Phospho-MRLC (Ser19)** | 3675 | Cell Signaling Technology | Mouse monoclonal | Human, mouse, rat | WB | Manufacturer datasheets: rigorously validated in-house by WB and IF; specific for Ser19-phosphorylated MRLC.  Used as orthogonal antibodies to ab2480 to confirm phospho-MRLC localization. | Ext. Data Fig. 9h, i |
| **MRLC (total)** | PA5-17624 | Thermo Fisher (Invitrogen) | Rabbit polyclonal | Human, mouse, rat | WB | Manufacturer datasheet: validated by WB. Used to assess total MRLC levels alongside phospho-MRLC for normalization. | Ext. Data Fig. 9h, i |
| **MRLC (total)** | 3672 | Cell Signaling Technology | Rabbit polyclonal | Human, mouse, rat | WB | Manufacturer datasheet: rigorously validated in-house by WB and IF; recognizes endogenous total MRLC. Used as orthogonal antibody to PA5-17624. | Ext. Data Fig. 9h, i |
| **GFP** | ab13970 | Abcam | Chicken polyclonal (IgY) | Recognizes GFP (Aequorea victoria) and derivatives | ICC (signal amplification of GFP-tagged constructs) | Manufacturer datasheet: validated by ICC/IF and WB; specifically detects GFP (UniProt P42212, ~27 kDa). Cited in 5,400+ publications since 2004 (CiteAb).  Specificity confirmed by absence of staining in non-GFP-transfected cells. | Ext. Data Fig. 8o |
| **GAPDH** | Acris | ACR001P | Mouse monoclonal | Human, mouse, rat | WB (loading control) | Manufacturer datasheet: validated by ICC/IF and WB. Cited in 38 publications (CiteAb). | Ext. Data Fig. 9h, i |

**Secondary antibodies.** All secondary antibodies (Alexa Fluor-conjugated and HRP-conjugated; Thermo Fisher and Jackson ImmunoResearch) were affinity-purified and cross-adsorbed against IgG of other species to minimize cross-reactivity. Each was validated by the manufacturer for the host species and application combination used.

***Abbreviations:*** *WB, western blot; ICC, immunocytochemistry; IF, immunofluorescence; IHC, immunohistochemistry; IP, immunoprecipitation; KO, knockout.*
