# Supplementary material for: An intrinsic cytoskeletal oscillator establishes neuronal polarity
Source: Nature. 2026 Jul 8;657(8130):213–25. doi: 10.1038/s41586-026-10755-6 (PMC13538037; doi:10.1038/s41586-026-10755-6)
Supplement: Supplementary file 5 [file 41586_2026_10755_MOESM5_ESM.zip › 2023-11-21494B-sv1-30/Supplementary Videos/Supplementary_Video_legends.docx]

**Supplementary Video 1.**

**Neurites of an unpolarized neuron exhibit extension-retraction oscillation**

DIV-1 mouse hippocampal neuron with segmentation of neurites. Time, hh:mm:ss; scale bar, 10 μm. Related to Fig. 1b and 1d.

**Supplementary Video 2.**

**Neurites of an unpolarized neuron exhibit extension-retraction oscillation**

Unpolarized cortical neuron expressing LynTM-mNeonGreen in cortical slice. Time, hh:mm:ss; scale bar, 10 μm. Related to Fig. 1c.

**Supplementary Video 3.**

**Neurite retraction is associated with actin wave formation at the soma**

Unpolarized cortical neuron in cortical slice expressing Lifeact-mNeonGreen. Time, hh:mm:ss; scale bar, 10 μm. Related to Fig. 1h.

**Supplementary Video 4.**

**Axons persistently grow without actin wave**

Axonal growth in cortical slice visualized with Lifeact-mNeonGreen. Time, hh:mm:ss; scale bar, 50 μm. Related to Fig. 1j.

**Supplementary Video 5.**

**Axon formation is associated with fewer actin waves during polarization**

DIV-1 mouse hippocampal neuron expressing Lifeact-mScarlet in polarization. Time, hh:mm:ss; scale bar, 20 μm. Related to Fig. 2a and 2g.

**Supplementary Video 6.**

**Axon persistently grows without actin waves after polarization**

DIV-3 mouse hippocampal neuron expressing Lifeact-mScarlet after polarization. Time, hh:mm:ss; scale bar, 20 μm. Related to Extended Data Fig. 2h.

**Supplementary Video 7.**

**Arp3 colocalizes with growth cone and actin patches**

DIV-1 mouse hippocampal neuron expressing Lifeact-mScarlet and Arp3-SNAP-SiR (high-temporal resolution). Time, hh:mm:ss; scale bar, 10 μm. Related to Fig. 3a.

**Supplementary Video 8.**

**Local increase of Arp3-actin patches precedes neurite extension**

DIV-1 mouse hippocampal neuron expressing Lifeact-mScarlet and Arp3-SNAP-SiR (long-term imaging). Time, hh:mm:ss; scale bar, 10 μm. The same cell of Supplementary Video 7. Related to Fig. 3b.

**Supplementary Video 9.**

**Inhibiting Arp2/3 with CK-666 abolishes growth cone and actin patches followed by neurite retraction**

DIV-1 unpolarized WT mouse hippocampal neurons expressing Lifeact-mScarlet treated with 150 µM CK-666. Time, hh:mm:ss; scale bar, 10 μm. Related to Fig. 3d and Extended Data Fig. 4d.

**Supplementary Video 10.**

**DIV-3 axon resists CK-666-induced retraction**

DIV-3 polarized WT mouse hippocampal neurons expressing Lifeact-mScarlet treated with 150 µM CK-666. Time, hh:mm:ss; scale bar, 20 μm. Related to Extended Data Fig. 4d.

**Supplementary Video 11.**

**CK-666 induces retraction of DIV-2 neurites, washout regrows the original neurites**

DIV-2 unpolarized WT mouse hippocampal neurons expressing Lifeact-mScarlet treated with 150 µM CK-666 and after removal of CK-666. Time, hh:mm:ss; scale bar, 20 μm. Related to Extended Data Fig. 4i.

**Supplementary Video 12.**

**CK-666 induces retraction of DIV-2 axon, washout regrows the original axon**

DIV-2 polarized WT mouse hippocampal neurons expressing Lifeact-mScarlet treated with 150 µM CK-666 and after removal of CK-666. Time, hh:mm:ss; scale bar, 20 μm. Related to Extended Data Fig. 4i.

**Supplementary Video 13.**

**PA-Rac1 photoactivation at a neurite tip induces active growth cone and neurite extension**

PA-Rac1 photoactivation at a growth cone of a mouse hippocampal neuron expressing Lifeact-mScarlet and PA-Rac1. Time, hh:mm:ss; scale bar, 10 μm. Related to Fig. 3k.

**Supplementary Video 14.**

**Suppressing Arp2/3 with PA-Rac1-T17N photoactivation at a growth cone induces growth cone collapse and retraction**

PA-Rac1-T17N photoactivation at a growth cone of a WT mouse hippocampal neuron expressing Lifeact-mScarlet and PA-Rac1. Time, hh:mm:ss; scale bar, 10 μm. Related to Extended Data Fig. 5c.

**Supplementary Video 15.**

**PA-Rac1 activation at multiple neurite tips induces limited number of growth cones**

PA-Rac1 photoactivation at multiple growth cones of a WT mouse hippocampal neurons expressing Lifeact-mScarlet and PA-Rac1. Time, hh:mm:ss; scale bar, 10 μm. Related to Extended Data Fig. 5d.

**Supplementary Video 16.**

**Intensities of actin patches and lamellipodium are inversely correlated over cycles of PA-Rac1 activation at the soma**

Unpolarized WT mouse hippocampal neurons expressing Lifeact-mScarlet and PA-Rac1. Example of actin patch-lamellipodium conversion. Time, hh:mm:ss; scale bar, 10 μm. Related to Extended Data Fig. 5g.

**Supplementary Video 17.**

**PA-Rac1 photoactivation at soma cortex induces wave formation and neurite retraction**

PA-Rac1 photoactivation at the soma of a polarized mouse hippocampal neuron expressing Lifeact-mScarlet and PA-Rac1. Time, hh:mm:ss; scale bar, 10 μm. Related to Fig. 3m.

**Supplementary Video 18.**

**Local perfusion of 150 μM CK-666 at the soma induces retrograde moving actin waves from the growth cone of axon**

Local perfusion of 150 μM CK-666 at the soma of a DIV-2 polarized neuron. Time, hh:mm:ss; scale bar, 20 μm. Related to Fig. 3p.

**Supplementary Video 19.**

**Arp3 and MRLC are localized as non-overlapping patch-like structures throughout the neuron**

Mouse hippocampal neurons expressing Lifeact-mScarlet, MRLC-mNeonGreen and Arp3-SNAP-SiR. Time, hh:mm:ss; scale bar, 10 μm. Related to Extended Data Fig. 6a and 6j.

**Supplementary Video 20.**

**Arp2/3 antagonizes actomyosin networks at the growth cone and drives extension**

Mouse hippocampal neurons expressing Lifeact-mScarlet, MRLC- SNAP-SiR and Arp3-mNeonGreen. Time, hh:mm:ss; scale bar, 10 μm.

**Supplementary Video 21.**

**Actomyosin networks disperse in the extending neurite**

Mouse hippocampal neurons expressing Lifeact-mScarlet, MRLC- mNeonGreen. Time, hh:mm:ss; scale bar, 10 μm. Related to Extended Data Fig. 6k.

**Supplementary Video 22.**

**PA-Rac1 activation transiently disassembles actomyosin arcs**

WT mouse hippocampal neurons expressing Lifeact-mScarlet, MRLC-SNAP-SiR and PA-Rac1 Time, hh:mm:ss; scale bar, 10 μm. Related to Extended Data Fig. 7g.

**Supplementary Video 23.**

**Inhibiting Arp2/3 with CK-666 induces contraction of actomyosin networks and neurite retraction**

WT mouse hippocampal neurons expressing Lifeact-mScarlet and MRLC-SNAP-SiR treated with 150 μM CK-666. Time, hh:mm:ss; scale bar, 10 μm. Related to Extended Data Fig. 7j.

**Supplementary Video 24.**

**Arp3 KO neurons lose actin patches, waves and growth cones and show reduced growth dynamics**

DIV-1 Arp3 KO mouse hippocampal neurons expressing Lifeact-mScarlet. Time, hh:mm:ss; scale bar, 10 μm.

**Supplementary Video 25.**

**Arp3 KO neurons have low neurite growth dynamics and no actin wave**

Arp3 KO cortical neuron in cortical slice expressing LynTM-mNeonGreen. Time, hh:mm:ss; scale bar, 20 μm. Related to Fig. 4n.

**Supplementary Video 26.**

**Electron tomograms of negative-stained actin network at neurite tips**

Electron tomograms of negative-stained actin filaments in WT and Arp3 KO neurite tips. Scale bar, 200 nm. Related to Fig. 4q.

**Supplementary Video 27.**

**Inhibiting Arp2/3 with CK-666 compresses microtubule bundles**

DIV-1 unpolarized WT mouse hippocampal neurons expressing Lifeact-mScarlet and EB3-SNAP-SiR treated with 150 µM CK-666. Time, hh:mm:ss; scale bar, 10 μm. Related to Fig. 5e and Extended Data Fig. 11d.

**Supplementary Video 28.**

**Inhibiting Arp2/3 with CK-666 induces microtubule kinks in the minor neurites but not in the axon**

DIV-3 polarized WT mouse hippocampal neurons expressing Lifeact-mScarlet and EB3-SNAP-SiR treated with 150 µM CK-666. Time, hh:mm:ss; scale bar, 20 μm. Related to Fig. 5e.

**Supplementary Video 29.**

**Removing CK-666 releases the compressed microtubules and recovers neurite growth**

DIV-1 unpolarized WT mouse hippocampal neurons expressing Lifeact-mScarlet and EB3-SNAP-SiR, after removal of CK-666. Time, hh:mm:ss; scale bar, 10 μm. The same cell of Supplementary Video 27. Related to Extended Data Fig. 11h.

**Supplementary Video 30.**

**Inhibiting myosin II with 20 µM blebbistatin promotes persistent neurite growth**

Live-cell video of WT and Arp3 KO mouse hippocampal neurons treated with 20 µM blebbistatin. Time, hh:mm:ss; scale bar, 10 μm. Related to Fig. 5l and 5m.
